# Nanometre-precision terahertz interferometry for battery electrode metrology

**DOI:** 10.1038/s41467-026-74193-8

**Published:** 2026-06-10

**Authors:** Guseon Kang, Jaeyoon Kim, Mee-Ree Kim, Younggeun Lee, Dong-Chel Shin, Jinwoo Jeon, Hyeonwoo Kim, Dae Hee Kim, Joohyung Lee, Sangbaek Park, Young-Jin Kim

**Affiliations:** 1 Department of Mechanical Engineering, Korea Advanced Institute of Science and Technology (KAIST), Daejeon, Republic of Korea; 2 Autonomous Manufacturing & Process R&D Department, Korea Institute of Industrial Technology (KITECH), Ansan, Republic of Korea; 3 Department of Materials Science and Engineering, Chungnam National University (CNU), Daejeon, Republic of Korea; 4 Department of Mechanical System Design Engineering, Seoul National University of Science and Technology (SEOULTECH), Seoul, Republic of Korea

**Keywords:** Terahertz optics, Sub-wavelength optics, Frequency combs, Characterization and analytical techniques, Batteries

## Abstract

High-precision, non-destructive thickness measurement is essential for lithium-ion battery (LIB) manufacturing. Existing approaches, such as X-rays, acoustic waves, and optical lasers, are limited by speed, resolution, or penetration into conductive materials. Here, we demonstrate nanometre-precision thickness measurements of LIB electrodes using terahertz (THz) Fabry–Pérot (FP) interferometry referenced to a photonic frequency comb. Combining the comb’s SI-traceable frequency accuracy with THz radiation’s immunity to scattering and absorption, our system directly detects FP modes with sub-10 MHz precision at sweep rates exceeding 12 THz/s, while spectral analysis simultaneously yields the complex refractive index. Electrode thicknesses of 50–150 μm are measured with nanometre precision: 70.1 nm (anode) and 465.5 nm (cathode) at 0.2 s, improving to 7.8 nm and 25.2 nm at 25.6 s, representing a one-to-two orders of magnitude improvement over temporal-analysis methods. The system further supports 3D profiling and dynamic thickness monitoring, enabling a unified, calibration-free platform for next-generation LIB metrology.

## Abstract (web summary)

This study showcases a breakthrough in the field of lithium-ion battery (LIB) metrology, demonstrating over a two-orders-of-magnitude improvement in precision for noncontact thickness measurements, and achieving nanometre-precision using frequency-comb referenced terahertz (THz) interferometry.

## Introduction

**Fig. 1:**
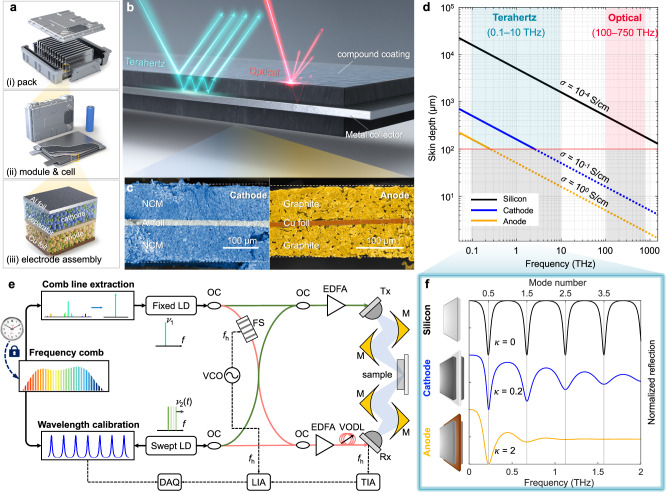
THz metrology for precision thickness measurements of LIB electrodes. **a** Structural hierarchy of a LIB system, showing the configuration from packs to the electrode assembly. **b** Schematic comparison of THz and optical beam behaviour during electrode inspection. THz waves exhibit deep penetration and low scattering, enabling multiple internal reflections within the electrode. In contrast, optical waves experience strong surface scattering and shallow penetration due to high absorption, limiting their ability to probe subsurface structures. **c** Cross-sectional SEM images of double-side-coated LIB cathode (left) and anode (right). **d** Calculated skin depth as a function of frequency for three different conductivities (silicon: ~10^−4 ^S/cm; LIB cathode: ~10^−1 ^S/cm; LIB anode: ~10^0 ^S/cm). Sample transparency (solid line: transparent, dotted line: opaque) is determined by comparing skin depth with sample thickness of 100 µm (horizontal red line). **e** System configuration of comb-based THz FP interferometry. **f** Simulated THz reflection spectra for three different conductivities. Each conductivity σ is scaled into the extinction coefficient *κ*, assuming refractive index *n* = 3.416 and sample thickness *t* = 100 µm. Normalised reflection signals are offset for clarity. LD laser diode, OC optical coupler, FS frequency shifter, VCO voltage-controlled oscillator, EDFA erbium-doped fibre amplifier, VODL variable optical delay line, Tx THz emitter, Rx THz receiver, M off-axis parabolic mirror, TIA trans-impedance amplifier, LIA lock-in amplifier, DAQ data acquisition.

Lithium-ion batteries (LIBs) are indispensable in electric vehicles (EVs), energy storage systems (ESSs), and portable electronics, where increasing energy density, longevity, and compactness demand tighter manufacturing tolerances^1^. Even minor structural non-uniformities—such as electrode thickness variation—can escalate into severe safety hazards of thermal runaway^2^. Each LIB electrode assembly consists of a cathode, anode, separator, and electrolyte (Fig. 1a), with material choices and design parameters such as thickness, porosity, and edge geometry critically affecting performance, safety, and thermal stability. However, the diversity of material compositions and structures presents a challenge for metrology, particularly in achieving high-resolution, high-speed, and non-destructive inspection. Moreover, the industry’s transition toward solid-state batteries^3,4^ increases the importance of identifying internal defects like delamination and voids. These trends underscore the need for a technique capable of simultaneously probing structure and material properties^5^, without compromising throughput or damaging the electrodes. Conventional techniques using X-rays, acoustic waves, and lasers have provided valuable structural insights for LIB electrode inspection^6–9^, but remain fundamentally limited by trade-offs among resolution, speed, and compatibility with conductive or heterogeneous materials. X-ray computed tomography (XCT) offers deep penetration and sub-micrometre voxel sizes capable of resolving internal structures and defects such as voids or misalignments, but its slow acquisition speed of minutes and high cost hinder practical deployment in high-throughput production lines^7^. Scanning acoustic microscopy (SAM) enables non-destructive subsurface probing by detecting internal acoustic reflections, but its lateral resolution is limited to tens of micrometres by long acoustic wavelengths, thickness precision typically remains at the micrometre level^6,8^. Laser displacement sensors (LDSs), which rely on visible^10^ (VIS, 0.4–0.8 µm) or near-infrared^11^ (NIR, 0.8–2.5 µm) reflections, are widely adopted for rapid surface profiling and thickness monitoring^9^. However, conductive and porous LIB electrodes exhibit strong optical absorption and scattering^12^, degrading accuracy and limiting penetration depth (Fig. 1b). In addition, because these sensors depend solely on surface-reflected signals, they cannot resolve individual layer thicknesses or detect internal defects (Fig. 1c). These limitations collectively highlight the need for alternative techniques that offer deeper penetration, resilience to scattering and surface roughness, and nanometre-level precision—key requirements for real-time, high-precision quality control in advanced battery manufacturing environments.

Terahertz (THz) radiation, with wavelengths ranging from 30 to 3000 µm (0.1–10 THz), offers a compelling solution for inspecting LIB electrodes, as its long wavelength minimises sensitivity to micrometre-scale surface roughness and enables deep penetration into porous^13^ or conductive^14^ materials; furthermore, THz spectroscopy allows extraction of material properties by analysing transmission *T*(*f*) or reflection *R*(*f*) spectra, which can be converted into complex refractive index, permittivity, and conductivity^15^—making it particularly suitable for battery electrodes (Fig. 1b). However, conventional THz systems—including time-domain spectroscopy (TDS) and frequency-domain spectroscopy (FDS)—have demonstrated thickness measurement capabilities. In TDS systems^16^, femtosecond lasers have been widely used to generate broadband THz pulses, with recent advances achieving ~30–40 nm precision^17,18^. Swept-laser-based FDS systems have also achieved sub-micrometre precision^19^, and sparse-frequency FDS methods have demonstrated fast acquisition times down to sub-milliseconds or ~60 nm precision with averaging^20^. However, for battery electrodes, reported precision remains at the hundreds of nanometres level^21^, and reliable industrial deployment demands SI-traceable frequency accuracy with long-term reproducibility. Stable and accurate THz sources offer a promising pathway to address these requirements, providing absolute frequency accuracy that enables consistent measurement reliability. A significant advancement has been the use of OFCs as photonic frequency dividers^22,23^, enabling the generation of ultra-stable THz radiation. Notably, direct optical heterodyning has demonstrated highly stable THz synthesis, where a pair of optical frequencies is either phase-locked^24,25^ or directly extracted^26^ from an OFC. Such approaches have achieved frequency stability at the 10^−15^ level and enabled linewidths as narrow as 2 mHz when stabilised to ultra-low-expansion optical cavities^26^. Despite these advances, challenges remain, including limited tuning range and measurement speed for high-precision thickness applications.

In this study, we present a frequency-comb-referenced THz FP interferometry for nanometre-precision, non-contact thickness measurement of LIB electrodes. By leveraging the SI-traceable absolute frequency accuracy of a photonic frequency comb and the strong scattering immunity of THz radiation, our system enables direct detection of Fabry–Pérot (FP) mode frequencies with sub-10 MHz precision at a high sweep rate exceeding 12 THz/s. This allows sub-micrometre thickness measurements across a wide dynamic range (50–150 μm) with high speed and accuracy—for instance, 70.1 nm and 465.5 nm precision at 0.2 s integration for a 103-μm-thick anode and a 105-μm-thick cathode, respectively, improving to 7.8 nm and 25.2 nm with 25.6 s integration—demonstrating an order-of-magnitude enhancement in single-shot measurements and up to two orders of magnitude with averaging compared to conventional temporal-analysis-based methods for battery electrode metrology. Additionally, our approach supports 3D surface profiling and real-time tracking of micrometre-scale thickness changes, enabling fast, in-line process monitoring. Importantly, through spectral fitting against transfer function simulations, our approach also retrieves material properties represented by complex refractive index without requiring prior calibration. This combination of nanometre-precision metrology, dynamic monitoring, and material characterisation establishes a unified, calibration-free platform for high-throughput, high-precision LIB electrode metrology, directly addressing the emerging demands of battery manufacturing.

## Results

### THz transparency of battery electrodes

Battery electrodes are generally considered optically opaque due to strong absorption of the VIS to NIR wavelengths. However, in the THz regime, they exhibit relatively lower absorption, making them partially transparent. This contrast in transparency is governed by skin depth δ, which determines how deeply an electromagnetic wave penetrates a conductive material before its amplitude decreases to 1/e. The skin depth is defined as δ  1/α = (πµσf)^-1/2^, where µ is the permeability, σ is the conductivity, and *f* is the frequency of the incident light^27^. Figure 1d presents the calculated skin depth as a function of frequency across the THz (0.1–10 THz) and optical (100–750 THz) domains for three different conductivities: 10^−4^, 10^−1^, and 10^0 ^S/cm, corresponding to silicon, LIB cathode, and LIB anode, respectively. Since skin depth determines material transparency, it provides a clear explanation for why battery electrodes behave differently in the THz and optical domains. Note that these conductivity values are order-of-magnitude estimates rather than exact values, based on DC conductivity measured by the four-point probe method. The grey-shaded region in Fig. 1d represents the opacity of the incident light source, determined by comparing skin depth with sample thickness (100 µm, red horizontal line). A material is considered transparent at a given frequency if its skin depth exceeds this threshold. This threshold increases twofold in a normal-incidence reflection setup and even further for oblique incidence. Two key factors explain this difference in transparency: First, THz waves have frequencies 2–3 orders of magnitude lower than optical sources, allowing them to penetrate more deeply. Second, in the THz regime, skin depth is primarily determined by material conductivity, which influences how much THz radiation can pass through a given material. For example, silicon (black line in Fig. 1d) has low conductivity, resulting in a millimetre-scale skin depth across the entire THz range. This makes silicon highly transparent, which is why silicon is commonly used for THz windows, lenses, and beam splitters. In contrast, the LIB cathode (blue line in Fig. 1d) has a sub-millimetre-scale skin depth in the 0.1–2 THz range, making it partially transparent in the THz domain and enabling the observation of FP interference signals from multiple internal reflections. For the LIB anode (yellow line in Fig. 1d), strong absorption results in a skin depth below 100 µm only at frequencies below ~0.3 THz, significantly limiting THz penetration at higher frequencies. Consequently, reliable thickness estimation in such highly absorptive materials strongly relies on low-frequency components for FP interferometry, as high-frequency signals become significantly attenuated for accurate analysis.

### Comb-based THz Fabry-Perot interferometry

Figure 1e shows a system configuration of frequency-sweeping THz interferometer referenced to an optical frequency comb (OFC). In this setup, an Er-doped fibre femtosecond laser provides a comb of optical frequencies, with both the repetition rate *f*_rep,_ and the carrier-envelope offset frequency *f*_ceo_ stabilised to an SI-traceable Rb atomic clock (see Methods for details). Two lasers—a fixed laser *v*_1_ and a swept laser *v*_2_(*t*)—are referenced to the comb to generate a frequency-sweeping THz wave *v*_2_(*t*)-*v*_1_ via optical-to-THz down-conversion. The optical beatnote *v*_2_(*t*)-*v*_1_ (green line in Fig. 1e) is delivered to a photomixer to generate a frequency-sweeping THz wave. This THz radiation illuminates the sample, where multiple internal reflections between front and back surfaces create FP interference fringes in the THz spectrum, sensitive to the sample thickness and refractive index^28^. The reflected THz wave is collected by a receiver photomixer, where it is heterodyned with another optical beatnote *v*_2_(*t*)-*v*_1_+*f*_h_ (red line in Fig. 1e), shifted by an intermediate frequency *f*_h_, enabling coherent detection of the amplitude and phase spectra (see Methods for details). In FP interferometry, the sample thickness *t* is determined from the resonance condition of the interference fringes. The resonance frequencies *f*_*m*_ of the *m*-th FP modes are given by:

1 $${f}_{m}={mc}/({2{ntcos}\theta }_{t})$$

where *m* is the mode number, *c* is the speed of light, *n* is the refractive index, *t* is the sample thickness, and *θ*_t_ is the angle the light travels through the sample. The mode number *m* is an integer *q* for constructive interference and *q* + 0.5 for destructive interference. Measurement of absolute resonance frequencies *f*_*m*_ directly yields the sample thickness *t*:

2 $$t={mc}/(2n{f}_{m}{\cos \theta }_{t})$$

Unlike traditional broadband approaches requiring wide spectral bandwidths for fringe counting^18,29^, our method leverages absolute frequency measurement of a single FP peak/dip or a few FP peaks/dips, enabling high-resolution thickness determination even over narrow spectral spans. This is particularly advantageous in the THz range, where broad high-SNR spectra are challenging due to low optical-to-THz conversion efficiencies.

Figure 1f presents simulated reflection spectra for three representative materials—silicon, LIB cathode, and LIB anode—characterised by extinction coefficients *κ* = 0, 0.2, and 2, respectively. To provide a comparative analysis of FP interference behaviours, simulations were performed using a finite-difference time-domain (FDTD) solver (Lumerical, Ansys), assuming a refractive index *n* = 3.416 and thickness *t* = 100 µm, over the 0–2 THz range. Silicon (black line in Fig. 1f), with minimal loss, reveals four well-resolved destructive FP modes (*f*_*m*=0.5, 1.5, 2.5, 3.5_), confirming that our system can measure ultra-thin wafers thinner than 50 µm, such as those used in high-bandwidth memory^30^ (HBM) and three-dimensional integrated circuits^31^ (3D ICs). The LIB cathode (blue line in Fig. 1f) also exhibits multiple FP modes, though the increased absorption reduces signal visibility at higher frequencies, emphasising the importance of detecting low-frequency FP modes (*f*_*m*=0.5, 1.5_) for improved SNR. For the LIB anode (yellow line in Fig. 1f), strong absorption suppresses interference above ~1 THz, leaving only low-frequency FP modes (*f*_*m*=0.5, 1.5_) observable, which are essential for reliable thickness estimation in highly absorptive materials.

### Frequency-sweeping THz nonlinearity compensation

**Fig. 2:**
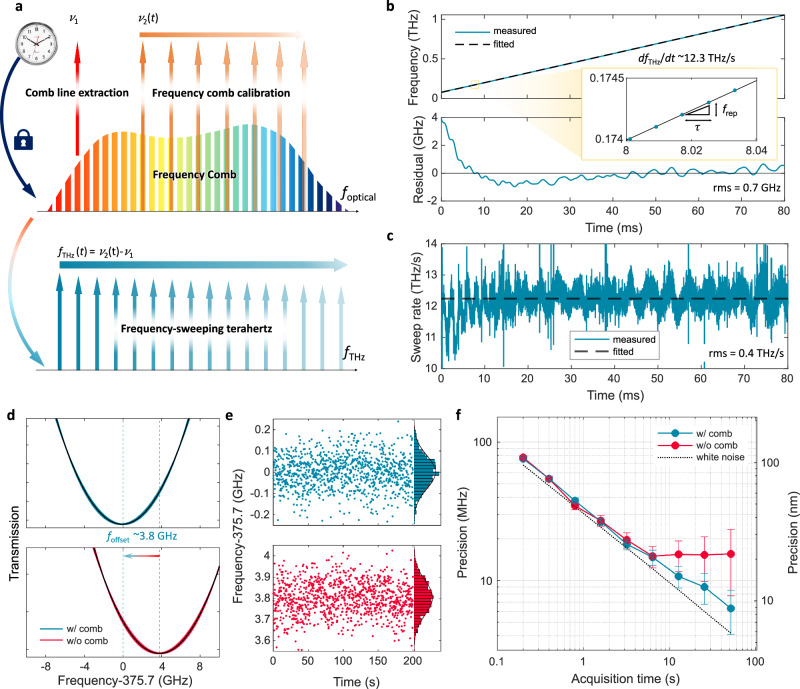
Frequency-sweeping THz spectroscopy with frequency comb calibration. **a** System schematic showing an optical frequency comb acting as a precision frequency ruler for two lasers in the optical domain (top), and an absolutely referenced frequency-sweeping THz signal through optical-to-THz down-conversion (bottom). **b** Measured THz frequency evolution during the sweep (top) and the frequency difference between the measured data and fitted curve (bottom). Inset, magnified view of measured data and fitted curve around 8 ms. **c** Instantaneous (blue) and nominal (black) sweep rate extracted from the data in (**b**). Comparison of FP mode measurements without (red) and with (blue) comb calibration: **d** Transmission amplitude spectra of a 525-µm-thick silicon wafer at normal incidence (*θ*_t_ = 0°), around *f*_*m*=4.5_ (375.7 GHz), **e** Frequency deviation of the FP mode with a histogram fitted to a Gaussian distribution, and **f** Allan deviation of the FP mode, with the white noise limit shown in black. Error bars represent ±1σ confidence intervals.

Figure 2a illustrates the concept of a comb-based calibration scheme employed to ensure absolute frequency accuracy and precision in the THz spectroscopy. An Er-doped fibre frequency comb, referenced to an SI-traceable Rb atomic clock, provides the absolute frequency grid for calibration. The fixed laser *v*_1_ is set at 1565 nm, while the swept laser *v*_2_(*t*) sweeps from 1564 to 1556 nm over time. The frequency difference *v*_2_(*t*)-*v*_1_ generates a continuously sweeping THz output *f*_THz_(*t*) in the range of 0.05–1.05 THz within 0.2 s via optical-to-THz down-conversion. The measurement bandwidth of our system can be flexibly tailored by adjusting the sweep range of *v*_2_(*t*). For instance, extending the sweep from 1564 to 1540 nm expands the THz bandwidth to 0.05–2.50 THz with an acquisition time of 0.5 s. Conversely, targeting a narrower bandwidth—for example, sweeping only 1564–1563 nm—enables faster measurements with an acquisition time as short as 0.02 s. This tunability of the THz-FDS bandwidth and update rate is achieved without sacrificing frequency accuracy, owing to the comb referencing technique that anchors all frequencies to SI-traceable standards. Frequency stability in this process is affected by two dominant factors: the stability of the fixed laser and the linearity of the swept laser’s tuning. To address these, both lasers are precisely referenced to the frequency comb (see Methods for details). The fixed laser *v*_1_ is stabilised by injection locking method to a specific comb mode^32^, ensuring that its absolute frequency remains fixed relative to the comb grid. Meanwhile, the swept laser *v*_2_(*t*) is calibrated by real-time heterodyne beat monitoring against the comb^33^, allowing the high-precision identification and correction of sweep nonlinearities. As a result, the collected THz spectrum is precisely frequency-calibrated, with each frequency point tied to the SI-traceable comb modes.

To evaluate the frequency nonlinearity inherent in swept-laser-based THz generation, we analysed the instantaneous frequency evolution during a sweep from 0.05 to 1.05 THz, as shown in top panel of Fig. 2b. A linear fit over a ~0.5 GHz segment within a ~40 µs interval reveals an average sweep rate of ~12.2 THz/s (inset of Fig. 2b). The frequency resolution of 0.1 GHz, defined by the comb’s mode spacing, is maintained by its high stability, ensured via locking to a Rb atomic clock. The bottom panel of Fig. 2b shows the residuals between the measured frequency trajectory and the linear fit, with deviations ranging from −1 to 4 GHz and a root-mean-square (rms) error of ~0.7 GHz, attributed to temporal nonuniformity. This nonlinearity, subject to environmental and instrumental fluctuations, directly impacts spectral accuracy and thus thickness precision. A more intuitive depiction is provided in Fig. 2c, which plots the instantaneous sweep rate *df*_THz_/*dt* = *f*_rep_/τ, where *f*_rep_ is the comb spacing and τ is the time interval between comb markers. The sweep rate exhibits a 0.4 THz/s rms fluctuation around the nominal 12.2 THz/s, highlighting the need for comb-based calibration to ensure high-fidelity frequency referencing. To evaluate the effectiveness of comb calibration in compensating sweep nonlinearity, we performed repeated measurements of well-defined FP modes in a silicon wafer, both without (red) and with (blue) comb calibration, and compared the spectral positions and frequency precisions (Fig. 2d–f). Comb calibration offers two significant benefits: it eliminates centre frequency offsets in FP modes for accurate thickness determination and significantly improves long-term precision by suppressing drift and noise accumulation.

Figure 2d shows the zoomed-in transmission spectra of 525-µm-thick silicon at normal incidence (*θ*_t_ = 0°) around the FP mode at *f*_*m*=4.5_ (375.7 GHz). Without calibration (red), the spectral position is offset compared to the calibrated case (blue). A centre frequency offset of ~3.8 GHz between the two template traces, corresponding to a thickness error of ~5.3 µm based on the relation *Δt* = *t*×(*f*_*m*_/*Δf*_*m*_), was successfully corrected by comb calibration. Figure 2e illustrates the frequency deviation of individual traces (blue and red traces in Fig. 2d) from the template (black curves in Fig. 2d). The effective line centre shift for each trace is determined by identifying the frequency shift that maximises its cross-correlation with the template. The centre frequencies of the FP modes were retrieved via Lorentzian fitting, and the corresponding thickness was calculated using Eq. (2). The right insets of Fig. 2e show histograms of the frequency deviations, which follow a Gaussian distribution, indicating that random noise is the dominant error source and suggesting that the averaging effect can improve precision. Figure 2f presents Allan deviation analysis to evaluate long-term measurement precision. For acquisition times shorter than 6.4 s, both free-running (red) and comb-calibrated (blue) systems show similar precision, closely following the theoretical white noise limit (black dotted line). However, beyond 6.4 s, the blue trace continues to improve, reaching several-MHz precision, whereas the red trace flattens out at a noise floor of ~15.6 MHz; in other words, comb calibration enables a thickness precision of 8.8 nm at 51.6-s integration, in sharp contrast to the uncalibrated case, where the best achievable precision is limited to 21.4 nm. This demonstrates the improved long-term stability and precision provided by the OFC stabilised to an Rb atomic clock, enabling nanometre-scale thickness precision in transparent materials such as silicon^34^. Furthermore, such comb-referenced reproducibility^23^, achieved without periodic recalibration, is essential for establishing reliable process-thickness correlations in industrial process development.

### Thickness measurements of battery electrodes

**Fig. 3:**
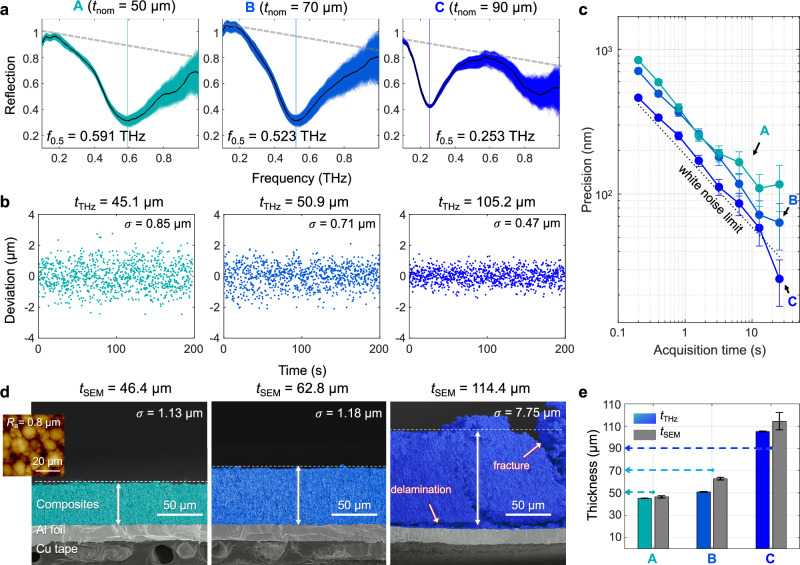
Measurement precision analysis of LIB cathodes. **a** Measured reflection amplitude spectra for three nominal thicknesses (case A, 50 µm; case B, 70 µm; case C, 90 µm), acquired with a period of 0.2 s over 200 s, with the FP mode *f*_*m*=0.5_ indicated by vertical lines. The grey dotted lines show a decreasing trend in amplitude due to skin effect. **b** Deviations of thickness *t*_THz_ retrieved from the centre frequency of the measured FP mode *f*_*m*=0.5_. **c** Thickness precision evaluated for each case based on the data in (**b**) with theoretical white noise limit; the error bars represent the ±1σ confidence intervals. **d** Cross-sectional SEM images used to determine *t*_SEM_. **e** Comparison of thicknesses measured by THz interferometry and those obtained from cross-sectional SEM imaging. Error bars indicate the measurement precision for *t*_THz_ and the spatial non-uniformity for *t*_SEM_, respectively.

In order to demonstrate the nanometre-precision and robustness of our approach against scattering and absorption, we measured the thickness of LIB cathodes with three nominal thicknesses *t*_nom_ (Fig. 3; case A: 50 µm, case B: 70 µm, case C: 90 µm), adjusted by varying the casting thickness in doctor-blade film coating (see Methods for details). Figure 3a presents the measured reflection amplitude spectra of LIB cathodes across 0.1–1 THz, with the centre frequency of the first destructive FP mode *f*_*m*=0.5_ indicated by vertical blue lines. The data represent 1000 traces, each set with a period of 0.2 s, collected over 200 s. As the frequency increases, two distinct spectral features become apparent: first, the amplitude fluctuations grow more pronounced, primarily due to the reduced SNR of the THz source at higher frequencies^35^ (Fig. 2a); second, a gradual decrease in the overall amplitude (grey dotted lines) is observed, which is attributed to the skin effect, resulting in increased attenuation in the higher-frequency region (Fig. 1f). As expected from Eq. (1), the FP mode *f*_*m*=0.5_ shifts toward lower frequencies with increasing thickness (cases A–C). While only a single FP mode is observed in cases A and B, case C exhibits an additional mode at *f*_*m*=1.5_, due to its increased optical path length. The thicknesses *t*_THz_ were retrieved using Eq. (2), assuming a complex refractive index of *n* + *iκ* = 2.9 + 0.5*i*, determined by comparing measured spectra and numerical simulations (see Fig. 5).

Using the same analysis procedure established in Fig. 2d—cross-correlation-based alignment to a template and Lorentzian fitting of the FP mode—we extracted the centre frequencies for each trace and converted them to thickness values via Eq. (2). Figure 3b shows the resulting deviations in retrieved thickness *t*_THz_, demonstrating sub-micrometre-level precision—defined as single-point measurement repeatability—for all three cases. The standard deviation σ decreases with increasing thickness, reflecting enhanced frequency stability at lower FP mode frequencies (see Methods for details). This trend is primarily attributed to the higher SNR of the electric field in the lower-frequency region (Fig. 2a), with measured SNRs at *f*_*m*=0.5_ of 42 dB, 30 dB, and 26 dB for cases A, B, and C, respectively. Figure 3c provides the thickness measurement precision, in terms of Allan deviations, as a function of acquisition times. The precision improves with increasing acquisition time, following the slope predicted by the theoretical white noise limit (black dotted line). For thinner samples, however, the precision performance degrades, primarily due to two factors: the higher frequency and the broader linewidth of the FP mode. Specifically, as the sample becomes thinner, the spectral linewidth of the FP mode increases, making it more challenging to accurately determine its centre frequency. Consequently, case A fails to achieve sub-100 nm precision, instead reaching a precision of 109.5 nm at 12.8 s acquisition time. This limitation highlights the need for enhanced fringe visibility in ultra-thin samples thinner than 50 µm, which could be addressed by introducing additional reflecting layers—such as a well-defined silicon substrate and air gap above the thin sample—to generate supplementary spectral content and strengthen interference contrast, a strategy we plan to explore in our forthcoming investigations. Importantly, cases B and C successfully achieved sub-100 nm precision at 8.4 s and 3.8 s, respectively, and further improved to 63.4 nm and 25.2 nm at 25.6 s integration—demonstrating capability as a reference-grade metrology tool for initial process optimisation—while sub-micrometre precision at 0.2 s supports applications requiring higher throughput. These results confirm the robustness of the system for monitoring the thickness of LIB electrodes.

To validate the accuracy of our THz interferometric approach, cross-sectional SEM imaging was performed on ion-milled LIB cathodes (see Methods). Figure 3d shows the cross-sectional SEM images for each case. While cases A and B display clean cross-sections, case C reveals localised fractures within the coating layer and delamination at the coating–substrate interface. This is attributed to non-optimised ion milling conditions, which were uniformly applied across all samples. Such structural damage introduces additional uncertainty in SEM-based thickness measurements, underscoring the need for carefully optimised sample preparation. The inset of Fig. 3d shows a topography image of the electrode’s surface obtained by atomic force microscopy (AFM; XE-100, Park Systems), revealing a surface roughness *R*_a_ = 0.8 µm. This value exceeds the roughness typically observed on the ground side (~0.5 µm) of a single-side polished silicon wafer, resulting in significant surface scattering that impairs the measurement capability of optical-domain sources. This necessity highlights the practical limitations of destructive methods and underscores the value of our non-destructive THz interferometric approach, which enables nanometre-level precision without the need for invasive sample preparation—such as iterative optimisation of ion-milling conditions for each electrode type— and is inherently immune to surface scattering. Figure 3e presents the comparative analysis of three types of thickness: nominal thickness *t*_nom_, THz-measured thickness *t*_THz_, and SEM-measured thickness *t*_SEM_. The measured thicknesses (*t*_THz_ and *t*_SEM_) deviate from nominal values (*t*_nom_) by several to tens of micrometres, demonstrating that empirical assignment alone cannot ensure the accuracy required for advanced electrode metrology. The error bars indicate the measurement precision for *t*_THz_ and the spatial non-uniformity for *t*_SEM_, respectively. Both *t*_THz_ and *t*_SEM_ represent the total coating thickness including porosity, as *t*_THz_ inherently accounts for pore space through the effective refractive index. The *t*_SEM_ values were determined by averaging the thickness across the entire cross-sectional SEM images after binarization processing. A detailed uncertainty analysis (Fig. S4) reveals three key uncertainty sources: sample geometry (~3.8 µm from surface roughness, flatness, and edge rounding), refractive index variation for THz interferometry, and ion milling artifacts for SEM imaging. The overall measurement uncertainties are estimated to be ~0.1 µm for THz interferometry—dominated by variations in the complex refractive index—and ~4.3 µm for cross-sectional SEM imaging—primarily due to sample damage and delamination during ion milling (see Methods for details).

**Fig. 4:**
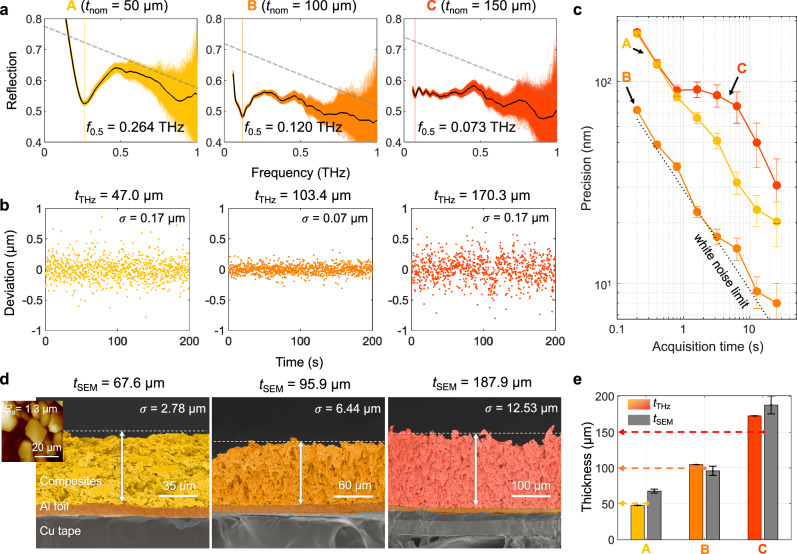
Measurement precision analysis of LIB anodes. **a** Measured reflection amplitude spectra for three nominal thicknesses (case A, 50 µm; case B, 100 µm; case C, 150 µm), acquired with a period of 0.2 s over 200 s, with the FP mode *f*_*m*=0.5_ indicated by vertical lines. **b** Deviations of thickness *t*_THz_ retrieved from the centre frequency of the measured FP mode. **c** Thickness precision evaluated for each case based on the data in (**b**). **d** Cross-sectional SEM images used to determine *t*_SEM_. **e** Comparison of thicknesses measured by THz interferometry and those obtained from cross-sectional SEM imaging. Error bars indicate the measurement precision for *t*_THz_ and the spatial non-uniformity for *t*_SEM_, respectively.

The LIB cathode measurements shown in Fig. 3 confirm that our THz FP interferometry achieves sub-micrometre precision and good agreement with nominal and SEM-derived thickness values, despite the presence of significant absorption and surface roughness. To further assess the robustness and general applicability of our method, we conducted equivalent measurements on LIB anodes (Fig. 4). Figure 4a shows the measured THz reflection amplitude spectra for all three samples, with nominal thicknesses *t*_nom_ of 50 µm, 100 µm, and 150 µm for cases A, B, and C, respectively. A pronounced extinction mode at *f*_*m*=0.5_ is marked by vertical lines, serving as the primary indicator for thickness extraction. Unlike cathodes, the anodes exhibit a higher content of conductive fillers and significant porosity, leading to stronger THz absorption and more rapid amplitude decay in higher-frequency regions (grey dashed lines). For all cases, the FP resonance frequencies shifted lower as the film thickness increased, consistent with an increased optical path length. Figure 4b presents the variations in retrieved thickness *t*_THz_ based on the centre frequency of the measured FP mode. For case A (*t*_nom_ = 50 µm), the single-measurement precision was ~172 nm. In case B (*t*_nom_ = 100 µm), the FP mode lies in a lower frequency region with improved SNR, leading to a markedly enhanced precision of ~70 nm. This improvement is also evident in the averaging-time-based precision analysis shown in Fig. 4c, where case B achieved 70 nm precision at 0.2 s acquisition, further improving to sub-10-nm precision at 25.6 s, following the theoretical white-noise trend. In contrast, case A improved only to ~21 nm under the same conditions due to its initially higher noise floor. Case C (*t*_nom_ = 150 µm) showed an initial precision of ~175 nm and similar short-term precision to case B. However, beyond an averaging time of ~1.6 s, the precision deviated from the white-noise limit and even deteriorated. This deterioration is attributed to two factors: First, the FP mode for case C lies at ~0.073 THz (corresponding to a wavelength of ~4.1 mm), a very low frequency where longer THz wavelengths pass less efficiently through the 4 mm aperture, reducing optical power and SNR. Second, the laser frequency sweep accelerates in this low-frequency region, and although frequency comb calibration is applied, residual nonlinearity can adversely affect the accuracy of FP mode detection (see Methods for details).

Figure 4d provides cross-sectional SEM images used to determine the reference thickness *t*_SEM_, showing that anodes have a rougher surface (*R*a ~1.3 µm) than cathodes; however, the longer THz wavelengths reduce scattering effects that would otherwise impact VIS or NIR-based measurements. Cross-sectional SEM analysis via ion milling revealed no evidence of delamination or cracking. Figure 4e compares the thicknesses obtained via THz interferometry with those from SEM imaging. Both methods yielded closely matching estimates relative to the nominal thicknesses, supporting the high accuracy of the THz approach. However, SEM imaging still exhibits larger error bars due to the inherent spatial non-uniformity of the electrode coating, as well as artifacts introduced during the ion milling process and the mismatch in spatial sampling between the millimetre-scale THz spot and the micrometre-width SEM cross-section. (see Methods for details).

Overall, the thickness of LIB electrodes was measured with nanometre precision by monitoring the first destructive FP mode *f*_*m*=0.5_. The precision of the thickness values retrieved from *f*_*m*=0.5_ was found to be influenced by two main factors. First, higher measurement precision was achieved when the FP mode appeared at lower frequencies, where absorption is minimal, and the SNR is higher. This trend indicates that thicker samples allow for more precise measurements. However, at very low frequencies below 0.1 THz, a reduction in performance can occur due to limitations in photomixing efficiency and decreased optical power transmission through the aperture, as observed in case C of Fig. 4. Second, materials with higher refractive indices *n* exhibited reduced thickness deviations, leading to improved measurement stability. This effect was evident in the improved performance observed for LIB anodes, which possess higher absorption and refractive indices, compared to LIB cathodes. These findings collectively confirm the robustness and versatility of our THz FP interferometry for nanometre-scale thickness measurement across different LIB electrode types. Such precision, combined with the long-term reproducibility of comb-referenced measurements, enables reliable characterisation of process-thickness correlations essential for electrode manufacturing optimisation. A quantitative comparison with existing THz metrology techniques is provided in the Methods section.

### Material characterisation of battery electrodes

**Fig. 5:**
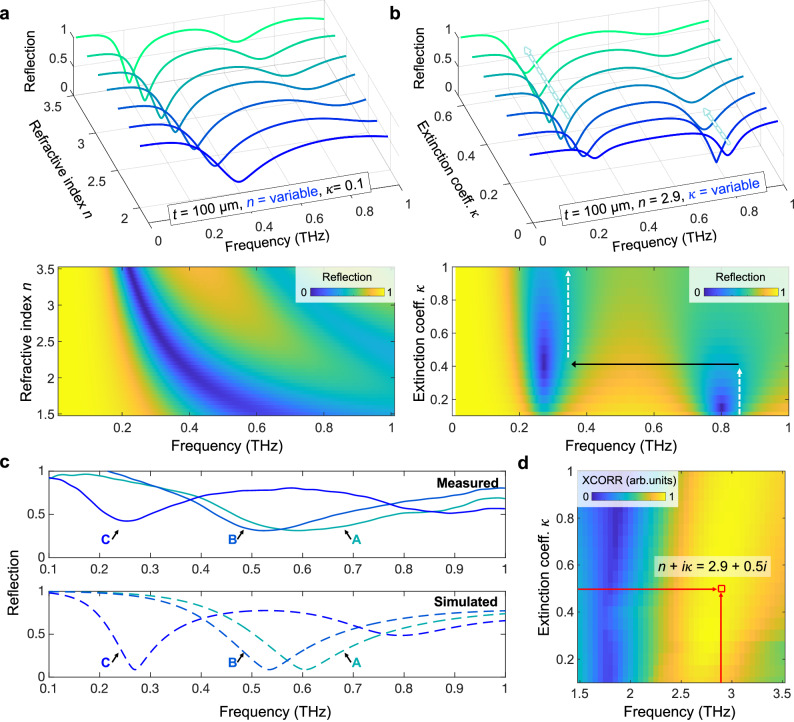
Material characterisation of LIB cathodes. Simulated reflection amplitude spectra for a 100-µm-thick sample showing the effect of varying the real part of the refractive index *n* (**a**) and the extinction coefficient *κ* (**b**), respectively; the top panel shows a 3D spectral profile, while the bottom panel presents a scaled 2D image for enhanced visibility. In **b**, the white dotted lines track the shift of dominant FP modes, showing a deeper dip, which is useful for determining the complex refractive index of arbitrary LIB cathodes. **c** Measured (top) and simulated (bottom) reflection amplitude spectra of LIB cathodes shown in Fig. 3, with the simulation based on the complex refractive index extracted via cross-correlation. **d** Calculated cross correlation coefficient for determining complex refractive index *n* + *iκ*.

Accurate determination of the complex refractive index *n* + *iκ* is critical for precise thickness measurements of LIB electrodes, yet remains challenging in the THz regime due to material heterogeneity and composite microstructure. To investigate its spectral influence, FDTD simulations were performed for a 100-μm-thick sample by varying either the real part *n* or the imaginary part *κ*. As *n* increases, systematic redshifts in FP modes are observed (Fig. 5a). In contrast, increasing *κ* causes the dominant (deepest) dip to transition from *f*_*m*=1.5_ at *κ* < 0.3 to *f*_*m*=0.5_ at *κ* ≥ 0.3 (Fig. 5b). This transition, highlighted by white dotted lines in Fig. 5b, serves as a useful spectral feature for constraining the complex refractive index of arbitrary LIB cathodes through model-based fitting.

Figure 5c, d present the comparison between measured and simulated reflection amplitude spectra and the corresponding cross-correlation coefficients (XCORR), respectively. Samples with multiple FP modes (i.e., case C of Fig. 5c) improved the accuracy of the XCORR calculation by providing stronger constraints for the cross-correlation fitting. Consequently, the complex refractive index for the cathode was estimated to be approximately 2.9 + 0.5*i*, and simulations based on this value reproduced the measured spectra with reasonable agreement, as seen in Fig. 5c. For the anode, the estimated complex refractive index was *n* + *iκ* = 6.0 + 3.4*i*, derived from case A which provides the broadest effective spectral bandwidth among the anode samples, with corresponding simulation results provided in Fig. S1. For reliable material characterisation, lower-absorption materials such as cathodes benefit from thicker samples that generate multiple FP modes, while higher-absorption materials such as anodes benefit from thinner coatings that enable broader spectral observation. This estimation of complex refractive indices enables monitoring and control of variations in electrode manufacturing conditions and composite material compositions. However, XCORR values remain broadly high across a range—for example, from 2.5 + 0.2*i* to 3.3 + 1.0*i*—indicating limitations in resolving unique optical constants (Fig. 5d). Since the complex refractive index *n* + *iκ* varies with electrode composition, porosity, and manufacturing process, in situ characterisation offers significant advantages over relying on pre-established values. In particular, porosity directly influences the effective refractive index through dielectric mixing, where increased porosity reduces *n*; however, because the FP resonance condition yields the optical thickness *nt*, independently separating *n* and *t*—and thus extracting porosity—requires additional measurement constraints. To address this, polarisation-resolved reflection spectroscopy can be employed. Oblique incidence (e.g., at a 45° angle) induces different Fresnel transmission and reflection coefficients^36^ for s- and p-polarised light, which manifest as distinct features in the polarisation-resolved spectra. Incorporating polarisation data is thus expected to enhance the precision of optical constant determination and will be further explored in future work. It is also important to note that, while the cross-correlation approach effectively estimates complex refractive indices, it is computationally intensive due to the need for exhaustive spectral simulations. Implementing deep-learning-based interpolation techniques^37^ could reduce computational cost and enable rapid material characterisation by generating high-resolution predictive spectra from lower-resolution datasets. This capability to simultaneously determine thickness and material properties positions the system as a stand-alone, reference-grade metrology tool for initial process development, where establishing optimal electrode coating conditions requires precise characterisation as functions of process parameters such as slurry composition, coating speed, and drying temperature—without the throughput constraints imposed by production-line conveyor speeds.

### Thickness profiling of 3D structures based on FP mode monitoring

The roll-to-roll (R2R) manufacturing process^38^ inherently leads to significant thickness variations at the edges of electrode coatings, making precise profiling of edge thickness crucial for ensuring cell assembly quality, performance, safety, and production costs. Sharp thickness transitions at the coating edges can induce defects such as wrinkles, tearing, or curling during curing, resulting in reduced manufacturing yield. Furthermore, non-uniformity introduced during stacking processes can create localised stress concentrations within the cell, which may lead to pouch swelling and compromise structural integrity^39^. To address these issues, THz spectral thickness profiling was explored as a potential solution for battery electrode applications. A 3D-printed sample made from polylactic acid (PLA) was selected as a test structure due to its THz absorption^40^ and ease of fabrication. PLA’s intrinsic absorption in the THz range makes it a suitable surrogate for electrode materials, while 3D printing enables precise fabrication of complex geometries to evaluate imaging performance. The sample was printed using a 3D printer (K13D, Creality), featuring a base thickness of 880 µm and a K-shaped protrusion reaching 1100 µm. The refractive index *n* = 1.57 was used for converting FP mode frequencies into thickness values via Eq. (2).

**Fig. 6:**
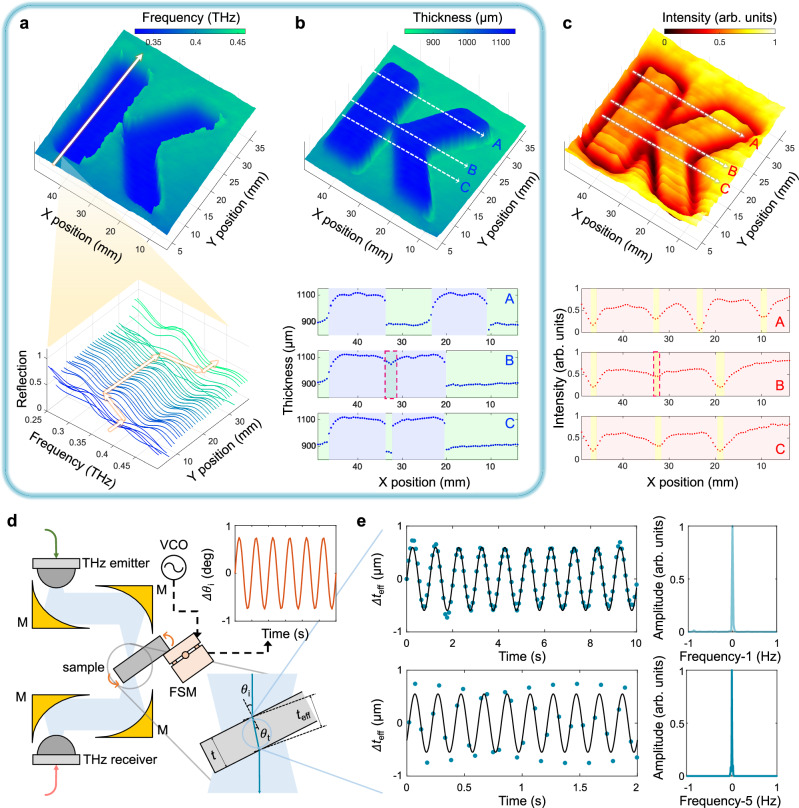
THz FP mode monitoring for thickness profiling and dynamic measurements. **a** 3D mapping of FP mode frequencies (top) for 3D-printed blocks with heights of 880 µm and 1100 µm, and variation of reflection spectra as the Y position is scanned while the X position is fixed at 45 mm (bottom); white arrow indicates the continuous shift of the FP mode at *f*_*m*=3.5_, used for thickness monitoring. **b** 3D thickness map (top) retrieved from the FP mode frequencies shown in (**a**), and thickness variations along the X direction while the Y position is fixed. Three cases are displayed: case A (*Y *= 29 mm), case B (*Y* = 19 mm), and case C (*Y* = 18 mm). **c** 3D intensity map (top) and intensity variations along the X direction with the Y position fixed. **d** Schematic of the dynamic thickness measurement setup, with the right-top inset illustrating angle modulation of the fast steering mirror for effective optical path length modulation. **e** Measured effective thickness modulations fitted with a sinusoidal curve, along with its corresponding Fourier transform. M off-axis parabolic mirror, FSM fast-steering mirror, VCO voltage-controlled oscillator.

Thickness profiling was performed by FP mode monitoring across the sample. Measurements covered a 50 mm × 50 mm area, scanned in linear steps of 0.5 mm. At each scanning point, a single-shot spectrum spanning 0.1–1.0 THz was acquired within 0.2 s. The top panel of Fig. 6a displays a 3D map of the FP mode frequency, focusing on a specific FP mode *f*_*m*=3.5_ located in the 0.32–0.46 THz range, which corresponds to wavelengths of 0.7–0.9 mm. The continuous shifts in FP mode frequency indicate localised variations in sample thickness. The bottom panel of Fig. 6a shows changes in the reflection spectra along the Y-direction at a fixed X-position of 45 mm. Figure 6b presents the corresponding 3D thickness map, derived by translating the measured FP mode frequencies using the defined complex refractive index. The resulting thickness distribution closely matches the nominal geometry of the printed sample. Cross-sectional profiles extracted along three reference lines (cases A, B, and C) through the K-shaped protrusion enabled the assessment of lateral resolution in the imaging system. In case C, a lateral resolution of ~2 mm was achieved, consistent with the theoretical Rayleigh resolution limit^41^ *d* = 0.61λ/*NA*. Given an optical configuration with a focal length *f* = 50.8 mm and an aperture diameter *D* = 101.6 mm, the numerical aperture *NA* was determined to be ~0.24, leading to a theoretical lateral resolution between 1.77 and 2.27 mm. The experimentally measured resolution agreed well with these predictions. Due to the THz beam diameter being several millimetres wide, the measured thickness reflects a spatially averaged value across each spot, which explains localised observations such as the thinner region found near *X* = 19 mm in case B.

Figure 6c shows a 3D intensity map (top) and intensity variations along the X-direction at a fixed Y-position (bottom). Intensity imaging clearly detects the edges of the K-shaped structure but struggles to resolve individual thickness levels with a 220 µm step height. This limitation arises because it depends on local reflectance variations, making it vulnerable to noise and environmental disturbances such as temperature fluctuations, vibrations, and alignment errors^42^. While calibration can relate intensity to thickness, such relationships are highly sample-specific and difficult to generalise. Moreover, the thickness resolution of intensity imaging is constrained by the detector’s dynamic range and often saturates at a few micrometres^43^. In contrast, frequency-monitoring-based spectral imaging offers greater precision and is largely immune to environmental factors, enabling nanometre precision and micrometre resolution for thickness measurements. This approach also demonstrated lateral resolution of ~2 mm, consistent with the Rayleigh diffraction limit. This resolution is well-suited for characterising coating edge profiles, where multi-layer thickness transitions are encoded in spectral FP modes. However, detection of micrometre-scale lateral defects remains challenging due to spatial averaging over the millimetre-scale beam diameter. Future work may explore deep-learning-based super-resolution techniques, which have demonstrated sub-diffraction-limit imaging^44,45^ (~λ/4), as well as structured illumination approaches using THz-compatible spatial light modulators^46^, to surpass this limit and extend applicability to micro-defect inspection.

### Misalignment-tolerant, dynamic thickness measurements

One of the notable strengths of our THz FP interferometry is its high tolerance to sample misalignment, as evidenced by stable FP mode detection even when the sample is tilted by 45° ± 1.5°. This robustness ensures reliable thickness measurements despite significant angular deviations, making the technique suitable for integration into high-speed manufacturing environments where perfect alignment cannot always be maintained. Our frequency-comb-referenced THz source can be combined with various optical configurations, with Eqs. (1) and (2) remaining universally applicable. To demonstrate the fast and precise dynamic measurement capability, we employed a transmission geometry with a tilted quartz wafer as the simplest experimental configuration. We implemented a setup using a fast-steering mirror (FSM; OIM102, Optics in Motion) to periodically modulate the incidence angle *θ*_i_, as depicted in Fig. 6d. The effective thickness *t*_eff_ (i.e., optical path length) varies according to the nominal thickness *t* and the propagation angle *θ*_t_, calculated via Snell’s law as *θ*_t_ = sin^-1^(sin *θ*_i_/*n*). By modulating the incidence angle Δ*θ*ᵢ within ±1.5°, we achieved thickness variations Δ*t*_eff_ of approximately ±0.8 μm, inducing corresponding FP mode frequency shifts Δ*f*_*m*_ of about ±0.9 GHz, consistent with Δ*f*_*m*_ = Δ*t*_eff_ × (*f*_*m*_/*t*_eff_). For fast dynamic measurements, we targeted a narrower bandwidth of 0.2 THz in the 0.1–0.3 THz range, focusing on FP modes around *f*_*m*_ = 200.5 ± 0.9 GHz. Figure 6e shows the measured effective thickness variations Δ*t*_eff_ at modulation frequencies of 1 Hz (upper panel) and 5 Hz (lower panel), exhibiting peak-to-peak amplitudes of ~2 μm, in good agreement with theoretical predictions. The slightly larger amplitude deviation at 5 Hz is attributed to additional mechanical vibrations induced by the FSM-mounted sample configuration at higher modulation frequencies. These results highlight the capability of our THz FP interferometry to perform precise, real-time thickness measurements even under rapid, dynamic conditions. Combined with the Rb-clock-stabilised frequency comb ensuring long-term measurement reproducibility without periodic recalibration, and the inherent robustness of long THz wavelengths and common-path configuration against mechanical vibrations and angular misalignments, our system reduces the need for elaborate vibration isolation setups and precise optical alignment typically required in NIR-based interferometric systems^47^. These features collectively fulfil key requirements for next-generation solid-state battery manufacturing and position our THz FP interferometry as a practical solution for future in-line quality control.

## Discussion

The THz FP interferometry presented in this work demonstrates nanometre-scale thickness measurements of LIB electrodes, achieving a precision of 70.1 nm within 0.2 s for a 103-μm-thick anode, which improves to 7.8 nm with 25.6 s averaging. Comparable performance was observed for a 105-μm-thick cathode, with precision reaching 465.5 nm at 0.2 s and refining to 25.2 nm upon longer integration. These results represent a substantial advancement over conventional non-destructive inspection techniques, such as XCT, SAM, and LDS, which are limited by lower spatial resolution and sensitivity to surface roughness or material absorption. Beyond structural measurements, our method offers the capability to simultaneously determine complex-valued material properties, such as refractive index and conductivity, without the need for prior thickness calibration. This dual capability of structural and material characterisation positions our system as a versatile tool for adaptive, calibration-free metrology in the rapidly evolving battery industry. Additionally, we demonstrated 3D surface profiling that resolves millimetre-scale surface features with sub-micrometre depth precision, overcoming the limitations of conventional THz imaging that rely solely on single-frequency intensity. Furthermore, the system exhibits high tolerance to angular misalignment, maintaining stable FP mode detection even when the sample is tilted by 45° ± 1.5°, and supports real-time tracking of dynamic thickness variations occurring at 5 Hz. This resilience under dynamic conditions addresses critical challenges in high-speed battery manufacturing lines where precise alignment cannot always be ensured. The combination of nanometre precision, high measurement speed, and misalignment resilience achieved by our THz FP interferometry paves the way for transformative advances in quality control, process optimisation, and defect detection in next-generation LIB and solid-state battery manufacturing. Moreover, the presented approach may serve as a foundation for establishing industrial standards for battery metrology, similar to the role played by optical frequency combs in precision spectroscopy and photonic integration.

## Methods

### Frequency comb stabilisation and referencing

The OFC used as the source comb in Fig. 1e is based on an Er-doped fibre femtosecond laser (C-fibre, Menlo Systems), providing a spectral bandwidth of 60 nm centred at 1550 nm, equivalent to a 4 THz frequency span. The comb mode has a mode spacing of 0.1 GHz, determining the repetition rate *f*_rep_. Both *f*_rep_ and the carrier envelope offset frequency *f*_ceo_ are fully stabilised to a reference clock. Consequently, each comb line is precisely defined at *v*_n_ = *f*_ceo_+*nf*_rep_ where *n* is an integer. The stabilised OFC serves a dual purpose by splitting its output into two branches: one for extracting a comb line to use as a stable fixed-frequency laser *v*_1_, and the other for calibrating the frequency nonlinearity of the fast-sweeping laser *v*_2_ (Fig. 2a).

For comb line extraction, the OFC output is set to a total power of 20 mW, with the power of each individual comb line ~200 nW. To isolate a single comb mode, a narrow-band spectral filter with a full-width at half maximum (FWHM) of 100 MHz is employed. This filter is realised by cascading a fibre Fabry–Pérot (FFP) filter with a fibre Bragg grating (FBG) filter. The extracted comb line is then injection-locked to a distributed feedback (DFB) laser diode, resulting in optical power amplification by a factor of 40–50 dB. Notably, this amplification does not introduce significant frequency shifts or linewidth broadening, thus preserving the spectral purity of the comb line. The fixed laser *ν*_1_ is set at 1565 nm.

The OFC also calibrates the frequency nonlinearity of the fast-sweeping laser (Phoenix 1400, Luna) with a tuning range of ~50 nm around 1550 nm via heterodyne beat detection (Fig. S2). As the swept laser crosses successive comb lines, it generates two beatnotes: one near *f*_b_ and the other near *f*_rep_–*f*_b_. These are filtered by a band-pass filter (BPF; cutoff frequency, 130 kHz–5 MHz), and the resulting signals are processed through a mixer-based RF envelope detector. The detected peaks indicate the exact times when the swept laser crosses individual comb modes, enabling precise time-to-frequency mapping. The output is then routed to an arbitrary waveform generator (AWG) to produce synchronised trigger pulses for a DAQ-based real-time measurement system. These comb markers reveal temporal variations in the sweep rate (Fig. 2b, c), highlighting the need for comb-based calibration to maintain spectral accuracy and achieve nanometre-precision measurements.

### Coherent detection of frequency-sweeping THz radiation

To enable coherent detection of THz amplitude and phase spectra, a heterodyne frequency *f*_h_ = 80 kHz is introduced using a frequency shifter (FS) comprising a pair of acousto-optic modulators (AOMs), with their RF drive frequencies referenced to the Rb atomic clock, which shift the fixed laser frequency *v*_1_ by *f*_a1_ = 40.04 MHz and *f*_a2_ = 39.96 MHz, respectively, such that *f*_h_ = *f*_a1_-*f*_a2_. The resulting shifted frequencies are combined with a rapidly swept laser *v*_2_(*t*), producing two optical beatnotes: *v*_2_(*t*)-*v*_1_ (green line in Fig. 1e) directed to the emitter (PCA-FD-1550-100-TX-1, Toptica), and *v*_2_(*t*)-(*v*_1_ + *f*_h_) (red line in Fig. 1e) directed to the receiver (PCA-FD-1550-130-RX-1, Toptica). The first beatnote drives THz generation via photomixing, while the second yields a heterodyne photocurrent at *f*_h_ upon mixing with the received THz field. This photocurrent corresponds to a complex current *J*(*t*) = |*E*_THz_(*t*)| |*I*_opt_(*t*)|e^*iϕ*(*t*)^ = *J*_0_e^*iϕ*(*t*)^, where *ϕ*(*t*) is the phase difference. The phase is expressed as *ϕ*(*t*) = 2π*f*_h_*t* + *Td*ω_B_(*t*), where *T* is the optical delay between the THz field and the optical beatnote, and ω_B_(*t*) = 2π(*v*_2_(*t*)-*v*_1_) is the instantaneous angular frequency of the beatnote. Differentiating yields *dϕ*(*t*)/*dt* = 2π*f*_h_+*Tv*_s_, where the sweep rate is defined as *v*_s_ = *d*ω_B_(*t*)/*dt*. This can be rewritten as *dϕ*(*t*)/*dt* = 2π(*f*_h_ + *f*_0_), with *f*_0_ = *Tv*_s_/(2π) representing the intermediate frequency arising from the sweep. The delay *T* is determined by path lengths: *T* = (*nL*_Tx_ + *L*_THz_-*nL*_Rx_)/*c*, where *n* is the refractive index of the fibre, *L*_Tx_ and *L*_Rx_ are the optical fibre lengths in the transmitter and receiver arms, *L*_THz_ is the free-space THz path length, and *c* is the speed of light. To suppress *f*_0_ and mitigate nonlinearities from the sweep, the variable optical delay line (VODL) in the receiver arm is adjusted to minimise *T* (see Fig. 1e). When *T* ≈ 0, the phase simplifies to *ϕ*(*t*) = 2π*f*_h_*t* + *ϕ*_0_, with *ϕ*_0_ being a constant offset. The resulting photocurrent is amplified by a transimpedance amplifier (DHPCA-300, Femto; 10^5 ^V/A), and demodulated into amplitude and phase using a lock-in amplifier (LIA; SR830, SRS) referenced to an 80 kHz local oscillator. The outputs are digitised by a 12-bit, 500 kSa/s DAQ (USB-6363, NI). The THz spectral resolution is governed by the DAQ sampling rate and sweep speed, giving ~25 MHz under current settings. Resolution can be improved to sub-kHz either by reducing sweep speed or using faster digitisers^48^ (>300 MSa/s), enabling more detailed spectral analysis. In order to minimize spectral artifacts from water vapour absorption lines, all measurements were performed inside a dry-air purge enclosure maintained at a relative humidity below 5%.

### Precision analysis of each FP mode

To evaluate the precision characteristics of each FP mode, we analysed the frequency and thickness precision as a function of mode number (i.e., frequency position) based on measurements of a silicon wafer. Figure S3a shows the transmission amplitude spectrum of a 525-μm thick silicon wafer, where eleven destructive FP modes (*f*_*m*=1.5_ to *f*_*m*=11.5_) are observed across the 0.1–1.0 THz range. As frequency increases, the SNR decreases, leading to degraded frequency precision (Fig. S3b).

The left panel of Fig. S3c presents the frequency precision in terms of Allan deviation for different FP mode numbers. The frequency precision generally improves as the acquisition time increases, following the theoretical white noise limit (black dotted line). Increasing mode number (i.e., higher frequencies) shifts the precision curves upward, indicating degraded precision. The dashed lines (*f*_*m*=1.5_ = 125.1 GHz and *f*_*m*=6.5_ = 551.6 GHz) indicate outliers that deviate from this trend. The first outlier (*f*_*m*=1.5_ = 125.1 GHz) is attributed to the large acceleration of the swept laser in this frequency region, as shown in the 0–10 ms interval of Fig. 2b, c. This results in degraded stability at this frequency, causing the frequency precision of *f*_*m*=1.5_ to initially appear favourable but deviate from the theoretical line in long-term stability. The second outlier (*f*_*m*=6.5_ = 551.6 GHz) arises from coupling between the FP mode position and the water absorption line at ~557 GHz. Since this experiment was conducted without humidity control, the overlap between the FP mode and the water absorption line acted as a disturbance, leading to degradation in both short-term and long-term stability. This indicates that humidity control is essential for precision measurements, particularly when FP modes couple with water absorption lines.

The right panel of Fig. S3c shows the thickness precision, which exhibits a different trend compared to frequency precision. This can be explained by the frequency-to-thickness precision conversion derived from differentiating Eq. (2). Assuming normal incidence (*θ*_t_ = 0°) and using the relation *f*_*m*_ = *m*·*f*_FSR_, the thickness precision *δt* is expressed as: *δt* = (*c*/2*nf*_*FSR*_^2^) × (1/*m*) × *δf*_*m*_. This relation reveals a division effect: while the thickness precision is proportional to the frequency precision *δf*_*m*_, it is inversely proportional to the mode number *m*. Consequently, even though frequency precision degrades at higher-order modes, the division factor (1/*m*) partially compensates for this degradation, resulting in a different behaviour of thickness precision as shown in Fig. S3d.

### Battery electrodes fabrications

Both LIB cathode and anode were fabricated via a blade-casting method. For the cathode, LiNi_0.6_Co_0.2_Mn_0.2_O_2_ (NCM; MTI) powder, Super P (TIMCAL), and PVDF were mixed in a weight ratio of 8:1:1 in N-methyl-2-pyrrolidone (NMP, 99.5%, SAMCHUN). For the anode, artificial graphite powder (AG; MTI) was used instead of NCM, while maintaining the same mixing ratio and binder system. The mixtures were homogenised using a ball miller to prepare uniform slurries. The resulting slurries were cast onto current collectors—carbon-coated Al foil for the cathode and Cu foil for the anode—using a doctor blade, with the wet film thicknesses adjusted to achieve target dry thicknesses, followed by drying at 110 °C under vacuum for 10 h.

### Ion-milling for cross-sectional SEM imaging

To obtain precise cross-sections of the electrodes, Ar ion milling was conducted using an ion milling system (ArBlade 5000, Hitachi). Direct milling of the cathode/anode films posed a high risk of sample damage due to the high-energy Ar ions. To mitigate this, additional processing steps were implemented to reduce the ion beam energy and improve sample stability during milling. The cathode/anode films deposited on a metal substrate were cut into rectangular pieces (10 × 5 mm^2^). Each sample was then mounted onto a 25 μm thick undoped silicon wafer using double-sided conductive copper tape. This configuration not only provided mechanical support but also helped distribute the ion beam impact more uniformly across the sample during milling. The assembled sample was mounted in the ion milling system with the silicon side facing the ion source. The total milling time was 2 h under reduced ion energy to minimize thermal and mechanical damage.

### Comparative uncertainty analysis of thickness measurements

To evaluate the reliability of thickness measurements, uncertainty sources were analysed for the sample, THz interferometry, and cross-sectional SEM imaging. Sample-induced uncertainties were primarily attributed to micrometre-scale surface roughness (~1.5 μm), deviations in flatness and parallelism, and edge rounding (~3.5 μm) of the electrode structure (Fig. S4a). These geometric imperfections were estimated to contribute an overall uncertainty of ~3.8 μm, regardless of the measurement method. Although such uncertainties can be minimised using calibration-grade specimens with well-defined geometries, LIB electrodes are not standardised by national metrology institutes (NMIs), making them particularly prone to sample-induced errors.

In the THz interferometry setup, system-related uncertainties were evaluated, with particular focus on the light source and interferometer (Fig. S4b). Frequency instability of the THz source—referenced to an optical frequency comb—was estimated as *t* × 10^−8^, where *t* is the nominal sample thickness, and considered negligible. Beam alignment errors due to angular deviations were estimated as *t* × 10^−5^ and were also minor. The dominant uncertainty was found to originate from variations in the complex refractive index of LIB electrodes, affected by material composition and layer heterogeneity. Considering all contributions, the overall uncertainty of the THz method was estimated to be ~0.1 μm. In contrast, uncertainty in cross-sectional SEM imaging was largely governed by the sample preparation process (Fig. S4c). While imaging-related factors such as resolution, magnification, and beam interaction were confined to the nanometre scale and deemed negligible, significant errors arose from the ion milling process. Due to material inhomogeneity and differential sputtering rates, artifacts such as the curtain effect^49^ and deviations from perpendicular cross-sectioning introduced substantial uncertainties, estimated as *t* × 10^−2^, leading to an overall measurement uncertainty of ~4.3 μm.

### Precision benchmarking with terahertz metrology

To benchmark our comb-referenced THz FP interferometry against existing techniques, we compiled thickness precision data from state-of-the-art THz metrology systems reported in the literature (Fig. S5). Precision values were categorised into single-shot measurements and averaged measurements where available. For battery electrodes (Fig. S5a), our system achieves 70.1-nm precision at 0.2-s acquisition for anodes, improving to 7.8-nm with 25.6-s integration—representing approximately one order of magnitude improvement in single-shot and two orders of magnitude with averaging compared to swept-laser-based FDS approaches^21^. For low-loss dielectrics^17–20^ and semiconductors^50^ (Fig. S5b), TDS systems have demonstrated single-shot precision down to several tens of nanometres^17^, while swept-laser-based FDS^19^ and sparse-frequency FDS^20^ systems achieve comparable precision at rapid acquisition times down to sub-milliseconds. Our comb-FDS system matches this single-shot performance while surpassing these results through extended averaging, reaching sub-10 nm precision—a regime previously unexplored in THz metrology. A key advantage of our comb-referenced approach is its SI-traceable frequency accuracy, which suppresses long-term drift and enables continued precision improvement following the theoretical white-noise limit even with extended integration. This inherent frequency accuracy eliminates the need for periodic recalibration or look-up table maintenance, providing consistent and reproducible measurements over extended operation periods—a critical requirement for industrial deployment.

## Supplementary information

Supplementary Information

Peer Review file

## Data Availability

The data that support the findings of this study are available from the corresponding author upon request.
