# Supplementary material for: Nanometre-precision terahertz interferometry for battery electrode metrology
Source: Nat Commun. 2026 Jun 10;17:7382. doi: 10.1038/s41467-026-74193-8 (PMC13402754; doi:10.1038/s41467-026-74193-8)
Supplement: Supplementary file 1 — Supplementary Information [file 41467_2026_74193_MOESM1_ESM.pdf]

Supplementary Information for

Nanometre-precision terahertz interferometry for battery electrode metrology

Guseon Kang[1,2], Jaeyoon Kim[1], Mee-Ree Kim[3], Younggeun Lee[1], Dong-Chel Shin[1], Jinwoo Jeon[1], Hyeonwoo Kim[1], Dae Hee Kim[1], Joohyung Lee[4], Sangbaek Park[3], and Young-Jin Kim[1,*]

[1]Department of Mechanical Engineering, Korea Advanced Institute of Science and Technology (KAIST), Daejeon 34141, Republic of Korea.

[2]Autonomous Manufacturing & Process R&D Department, Korea Institute of Industrial Technology (KITECH), Ansan 15588, Republic of Korea.

[3]Department of Materials Science and Engineering, Chungnam National University (CNU), Daejeon 34134, Republic of Korea.

[4]Department of Mechanical System Design Engineering, Seoul National University of Science and Technology (SEOULTECH), Seoul 01811, Republic of Korea.

*Corresponding author: yj.kim@kaist.ac.kr (Y.-J, Kim)

This file includes:

Supplementary Figures 1 to 5

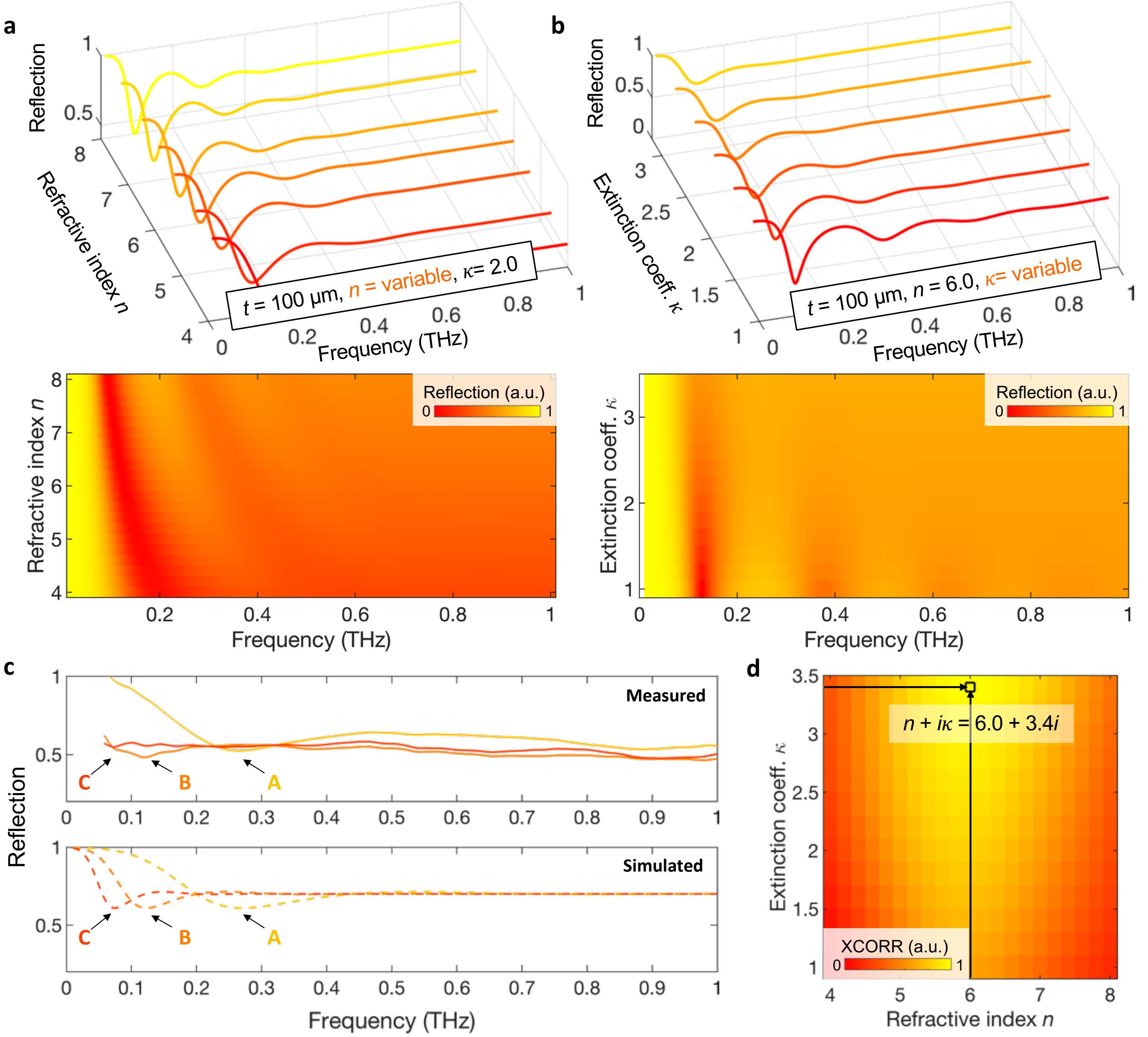

Fig. S1. Material characterization of LIB anodes. a, b Simulated reflection amplitude spectra for a 100-µm-thick sample showing the effect of varying the real part of the refractive index *n* (**a**) and the extinction coefficient *κ* (**b**), respectively; the top panel shows a 3D spectral profile, while the bottom panel presents a scaled 2D image for enhanced visibility. **c** Measured (top) and simulated (bottom) reflection amplitude spectra of LIB anodes shown in **Fig. 4**, with the simulation based on the complex refractive index extracted via cross-correlation. **d** Calculated cross-correlation coefficient for determining the complex refractive index $n+i\kappa$.

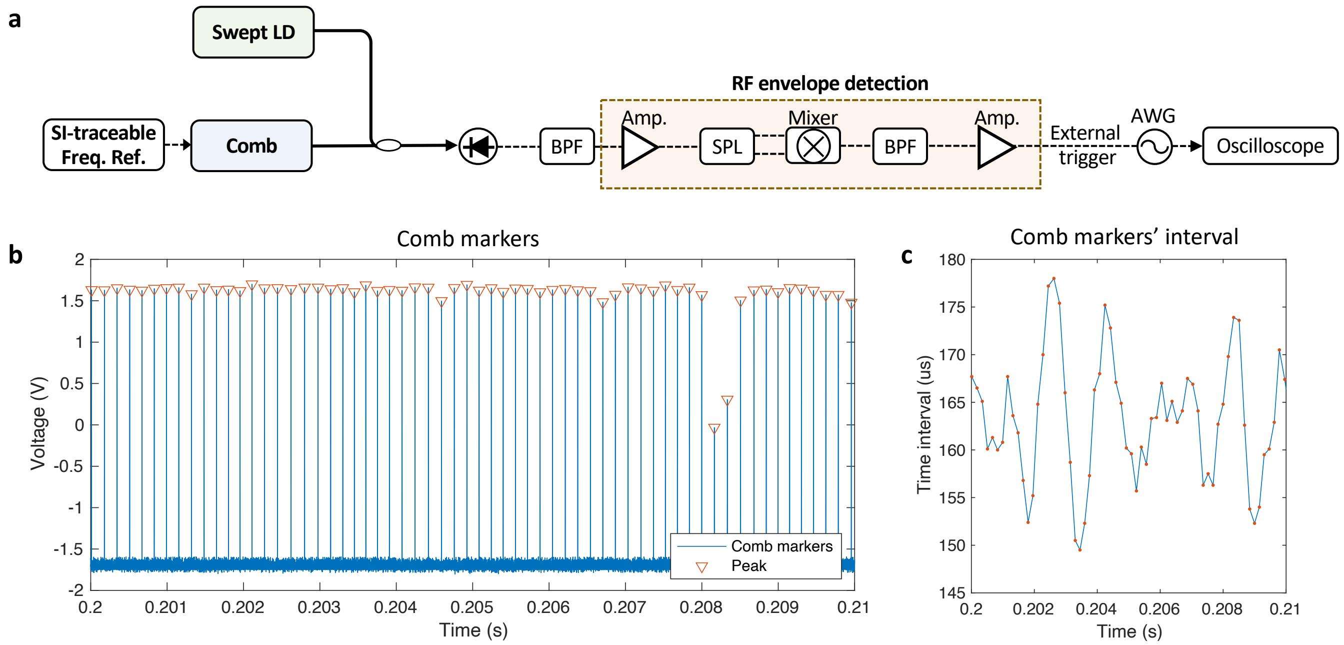

Fig. S2. Comb-based calibration of swept laser frequency. a Experimental setup for compensating the frequency nonlinearity of the swept laser using an optical frequency comb (OFC). **b** Comb calibration markers recorded on an oscilloscope. Each calibration tick is spaced by 100 MHz and traceable to the stabilized OFC, enabling absolute frequency calibration. **c** Measured time intervals τ between adjacent comb markers, with an average value of ~165 µs, illustrating the nonlinearity of the frequency sweep at a rate of ~6.1 THz/s. LD: laser diode, BPF: band-pass filter, Amp: RF amplifier, SPL: splitter, AWG: arbitrary waveform generator.

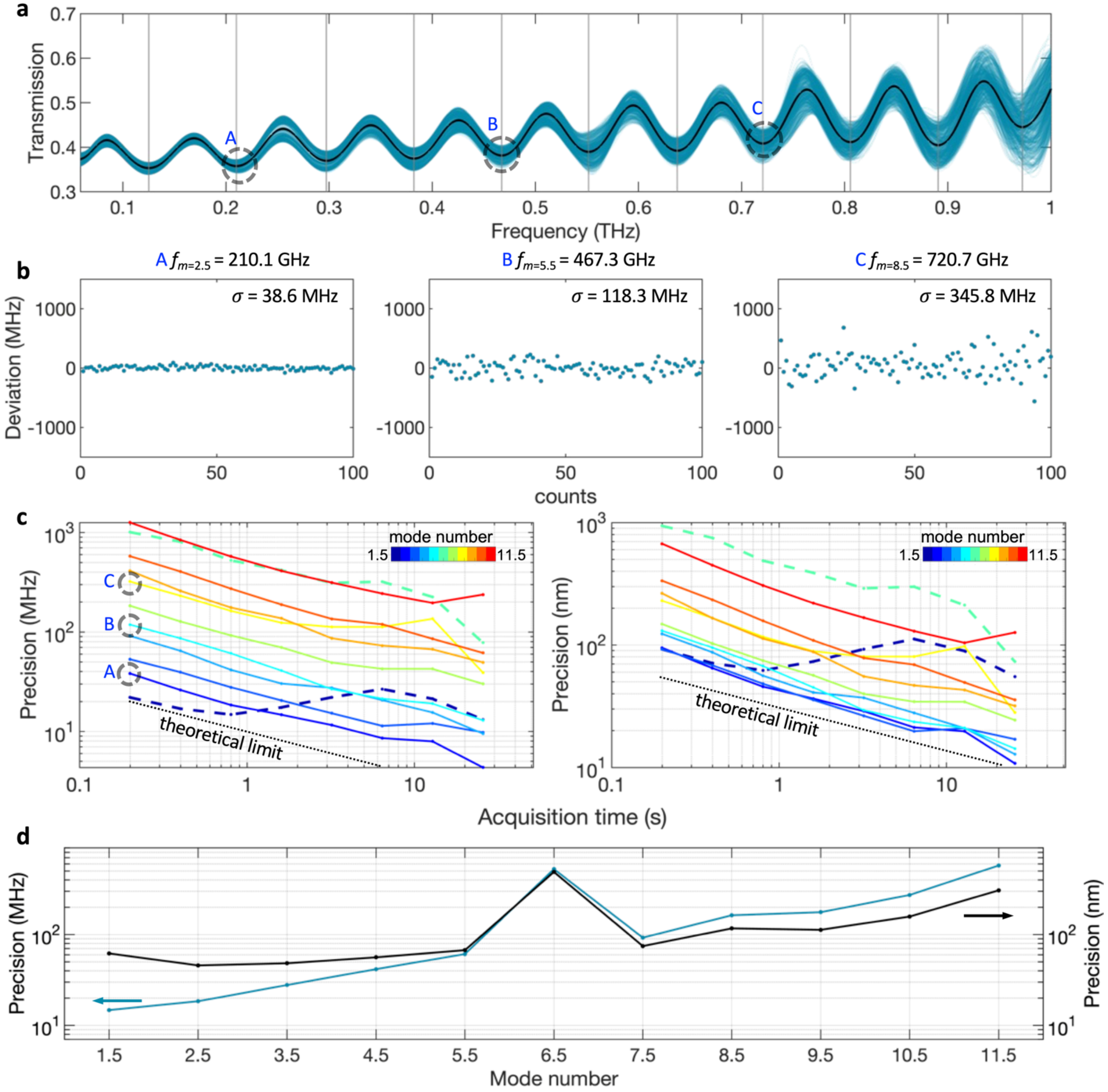

Fig. S3. Precision analysis of frequency and thickness measurements as a function of FP mode number. a Transmission amplitude spectra of a 525-µm thick silicon wafer measured over 1,000 repeated acquisitions across the 0.1–1.0 THz range. The black solid line represents the averaged spectrum, and grey vertical lines indicate the centre positions of destructive FP modes. **b** Deviation of FP mode centre frequencies for three different FP modes (Case A: $f_{m=2.5}$ = 210.1 GHz, Case B: $f_{m=5.5}$ = 467.3 GHz, Case C: $f_{m=8.5}$ = 720.7 GHz). **c** Allan deviation of FP mode frequency precision (left panel) and the retrieved thickness precision (right panel). **d** Frequency precision (blue, left y-axis) and thickness precision (black, right y-axis) as a function of mode number at 0.8-s acquisition time. The thickness precision shows a different behaviour compared to frequency precision due to the inverse dependence on mode number *m*.

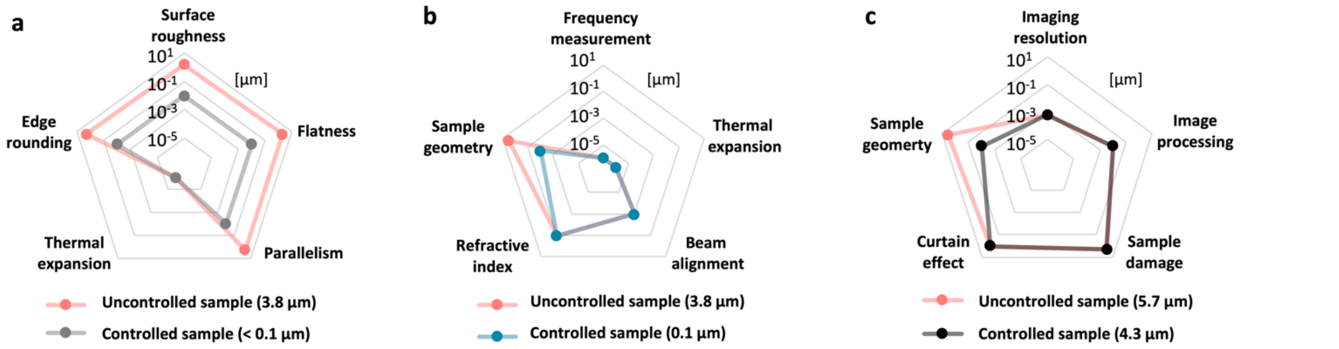

d

		Uncertainty errors	Value	Value for t = 100 µm
Sample		Uncertainty of surface roughness	1.5 µm	1.5 µm
		Uncertainty of flatness, parallelism, and edge rounding<br>Uncertainty of flatness = 1.5 µm<br>Uncertainty of parallelism = 1.5 µm<br>Uncertainty of edge rounding = 1.5 µm	3.5 µm	3.5 µm
		Uncertainty related to Thermal expansion of electrodes = $t \times 10^{-8}$<br>Thermal expansion coefficient of electrodes = 1×10^{-6} K^{-1}<br>Surface temperature = 15×10^{-3} K	$t \times 10^{-8}$	1×10^{-6} µm
Combined standard uncertainty (k=1)			$\{(t \times 10^{-8})^2+(3.8\ \mu m)^2\}^{1/2}$	3.8 µm
THz interferometer	Light source	Uncertainty related to frequency = $t \times 10^{-8}$<br>Repetition rate = 10^{-12}<br>Carrier-envelope offset frequency = 10^{-13}<br>Beat frequency between swept laser and comb = 10^{-8}	$t \times 10^{-8}$	1×10^{-6} µm
	Interferometer	Uncertainty related to beam alignment = $t \times 10^{-5}$<br>Uncertainty of sample incidence angle = 1°	$t \times 10^{-5}$	0.001 µm
		Uncertainty related to thermal expansion of optical fiber = $t \times 10^{-9}$<br>Thermal expansion coefficient of optical fiber = 5.0×10^{-7} K^{-1}<br>Surface temperature = 15×10^{-3} K	$t \times 10^{-9}$	1×10^{-7} µm
		Uncertainty related to refractive index of sample = $t \times 10^{-3}$<br>Uncertainty of refractive index = 10^{-3}	$t \times 10^{-3}$	0.1 µm
Combined standard uncertainty (k=1)			$t \times 10^{-3}$	0.1 µm
SEM imaging	Imaging system	Uncertainty related to imaging resolution = 0.0006 µm	0.0006 µm	6×10^{-4} µm
		Uncertainty related to image processing and analysis<br>Uncertainty of magnification calibration = 1%	$t \times 10^{-4}$	0.01 µm
	Cross-sectioning	Uncertainty related to sample damage and delamination<br>Uncertainty of sample damage = 3.0 µm<br>Uncertainty of delamination = 3.0 µm	4.2 µm	4.2 µm
		Uncertainty related to curtain effect of multilayer sample = $t \times 10^{-2}$<br>Uncertainty of sample perpendicularity = 5°	$t \times 10^{-2}$	1 µm
Combined standard uncertainty (k=1)			$\{(t \times 10^{-2})^2+(4.2\ \mu m)^2\}^{1/2}$	4.3 µm

Fig. S4. Uncertainty analysis in LIB electrode thickness measurements. a Sample-related uncertainties, where surface roughness, flatness, parallelism, and edge rounding contribute micrometre-scale errors, while thermal expansion is negligible. Sample-related uncertainties can be minimized using calibration-grade specimens, but LIB electrodes are not standardized by NMIs. Panels **b** and **c** show comparisons between uncontrolled and controlled samples (uncertainty < 0.1 µm). **b** THz interferometry uncertainties, dominated by variations in the sample's refractive index; errors from the light source and interferometer are minimal. **c** Cross-sectional SEM imaging, where uncertainty is primarily due to cross-sectioning accuracy, while imaging system noise is negligible. **d** Summary of uncertainty sources associated with the sample (**a**), THz interferometer (**b**), and cross-sectional SEM imaging (**c**).

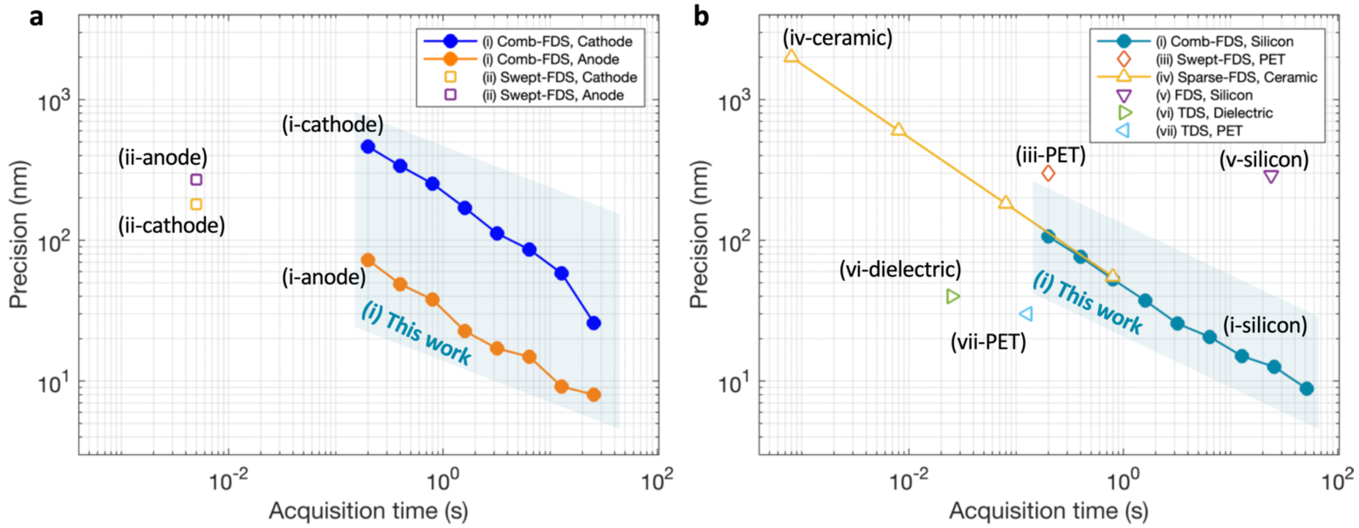

Fig. S5. Performance comparison with state-of-the-art terahertz thickness metrology in terms of thickness precision vs. acquisition time. a Battery electrode measurements; (i) this work, comb-referenced FDS (cathode and anode), (ii) swept-laser-based FDS (cathode and anode)[21]. This work (shaded region) achieves 70.1-nm precision at 0.2-s for anodes, improving to sub-10 nm at 25.6-s integration—demonstrating one-to-two orders of magnitude improvement over swept-laser-based FDS for battery electrode metrology. **b** Low-loss dielectric and semiconductor measurements; (i) this work, comb-referenced FDS (silicon), (iii) swept-laser-based FDS (PET)[19], (iv) sparse-frequency FDS (ceramic)[20], (v) FDS (silicon)[50], (vi) TDS (dielectric coating)[17], (vii) TDS (PET)[18]. Our system achieves comparable precision of several tens of nanometres in single-shot measurements, while reaching sub-10 nm precision with averaging—a regime previously unexplored in terahertz metrology.
