# Supplementary material for: Nanometre-precision terahertz interferometry for battery electrode metrology
Source: Nat Commun. 2026 Jun 10;17:7382. doi: 10.1038/s41467-026-74193-8 (PMC13402754; doi:10.1038/s41467-026-74193-8)
Supplement: Supplementary file 2 — Peer Review file [file 41467_2026_74193_MOESM2_ESM.pdf]

nature portfolio

Peer Review File

Nanometre-precision terahertz interferometry for battery electrode metrology

Corresponding Author: Professor Young-Jin Kim

This file contains all reviewer reports in order by version, followed by all author rebuttals in order by version.

Version 0:

Reviewer comments:

Reviewer #1

(Remarks to the Author)
This manuscript presents a novel and highly impactful approach to non-contact, nanometre-precision thickness measurement of lithium-ion battery (LIB) electrodes using frequency-comb-referenced terahertz (THz) Fabry-Perot (FP) interferometry. The work is timely, well-executed, and addresses a critical metrology gap in advanced battery manufacturing, particularly for solid-state and high-throughput production lines. The integration of an optical frequency comb for SI-traceable frequency calibration, combined with the inherent penetration and scattering resistance of THz radiation, enables unprecedented precision (e.g., 7.8 nm for anodes at 25.6 s integration) and high measurement speeds (>12 THz/s). The study is thorough, with systematic validation across different electrode materials, thicknesses, 3D profiling, and dynamic measurements. The manuscript is well-organized, the figures are clear, and the writing is generally fluent.

I recommend acceptance after minor revisions.

While the manuscript is strong, the following points could enhance its clarity, depth, and impact:

1.Material property generalizability:
The complex refractive indices used for cathodes (2.9+0.5i) and anodes (6.0+2.0i) are derived from specific samples. Please comment on how these values may vary with electrode composition, porosity, or manufacturing process, and how such variations could affect measurement accuracy in industrial settings.

2.Performance limits for ultra-thin electrodes (<50 μm):
The study notes reduced precision for thinner samples due to broader FP modes. Could the authors briefly suggest potential strategies to improve fringe visibility in such cases (e.g., enhanced reflection layers, multi-mode analysis) or define the practical lower thickness limit of the method?

3.Lateral resolution in imaging applications:
The lateral resolution is limited to ~2 mm due to the long THz wavelength. Although deep-learning super-resolution is mentioned as future work, a short discussion on the implications of this limitation for in-line inspection of fine electrode features (e.g., coating edges, micro-defects) would be helpful.

4.Clarity in uncertainty reporting:
The uncertainty analysis is detailed in Supplementary Fig. 3 but only briefly summarized in the main text. I recommend adding a concise summary of the key uncertainty sources (sample geometry, refractive index variation, SEM preparation artifacts) and their magnitudes in the Results or Methods section.

5.Comparative table with existing techniques:
A concise table comparing the proposed method with XCT, SAM, LDS, and conventional THz-TDS/FDS in terms of resolution, speed, penetration, material compatibility, and cost would provide readers with a quick overview of the advantages. The conclusion states that the precision is improved by two orders of magnitude compared with similar methods relying on temporal analysis, but the following are not clarified. The specific type of "traditional method" used for comparison (e.g., a certain brand of laser displacement sensor, a specific X-ray system) and its experimental conditions (e.g., integration time, sample thickness).

6. Verification in Actual Electrode Production Environments:
While the manuscript compellingly demonstrates the technical capabilities of THz FP interferometry in a controlled laboratory setting, its practical impact would be significantly strengthened by addressing real-world manufacturing challenges more directly (e.g., continuous operation stability and environmental factors (vibration, dust, humidity)). It is recommended to include a dedicated section discussing the path toward industrial deployment.

Minor editorial suggestions:

1.P. 5-6, line 110-111: The transition “This capability establishes…” feels slightly abrupt. Consider a smoother link to the preceding sentence.

2.Some figure references in the text (e.g., description of Fig. 2a) could be more precisely aligned with the visual elements.

3.Ensure consistent use of “LIB” vs. “Li-ion battery” throughout.

Reviewer #2

(Remarks to the Author)
The authors report on an optical-frequency-comb stabilized THz measurement technique to determine the layer thickness of battery electrodes with a precision down into the few nanometer range. This increased precision is reached due to the frequency stabilization which allows for the exact calculation of the layer thickness using the absolute position of the Fabry-Perot fringes generated due to multiple reflections inside the layer. Thus, this method works reliably only if the materials have high enough transparency in comparison to the skin depth. For the battery materials under investigation this is true for the low THz frequency range up to 1 THz.

While in TDS systems interferometric stabilization has already been introduced some time ago, using a frequency comb for stabilization is a novel approach, especially allowing for very fast frequency sweeps as demonstrated in this manuscript. The precision reached for 0.2s integration time is 70.1 nm for the anode, which is usually the more challenging electrode to be characterized. Increasing the integration time to 25.6 s, the precision is reported ot fall down to 7.8 nm - a truly impressive value compared to the commonly reached precision of a few 100 nm using commercial TDS systems.

A few points need further clarification prior to a possible publication:

1. For industrial inspection, 25.6 s integration time per measurement point will be prohibitive, as fabrication speeds of battery electrodes reach meters/second but even 70 nm are perfectly in range of the industrial requirements. What is the reason that the anode data surpass the cathode data although the anode usually is more absorbing? Please, clarify.

2. In Fig. 1 the measurement setup ist presented. Here, it is obvious that the samples need to be measured in reflectance geometry due to the opaque metal collector foil. Thus, it is not clear, how this relates to the angle dependent measurements shown in Fig. 6, in which clearly a geometry in transmission is required. This change in experimental geometry is not mentioned in the main text - or I have overlooked it. Especially in reflection geometry angles as large as the stated 45° will be prohibitive. Thus, the whole section on the angle dependent measurement needs clarification. Furthermore, the authors nicely demonstrate that they can measure under different angles and they identify the resulting variation in thickness. For inspection, the effective thickness is not important but the real thickness. How can the real thickness be retrieved without knowing (and measuring) the angle, under which the data have been taken? In Fig. 6 the caption stops after figure part (c). The angle dependent parts seem to have been added later. They are thus not found in the caption and need to be included.

3. In Fig. 1 f, the notion (a.u.) needs to be removed as normalized data are shown. Due to the normalization all units are normalized out so that indeed only numbers without units are left. This applies for all other normalized plots also.

4. In Fig. 2 the Allan deviation is plotted, very convincingly showing the improvement due the frequency comb stabilization. However, the true benefit starts from integration times higher than several seconds, which - to our experience - is way too long for industrial inspection. Additionally, what is the tolerance requested by industry for the electrode thickness? A few nanometers really asked for?

5. Fig. 3: The idea to use the same coloration in (d) than in (e) is very nice, but in this case the color chosen for t_SEM=99 µm needs to be adjusted either in (d) or in (e) to really match the image. In figure (e) the legend for t_THz would then also need an adjustment, e.g. include all threee colors into the box. It would also help to state the accquisition time needed to retrieve the data shown in (a) and (b) (same for Fig. 4) already in the figure caption. In (b) the long term stability is demonstrated, showing several measurements over a larger stretch of time, correct?

6. Fig. 6 (a) and (b): The camera image appears in my printout as a solid grey box with no features whatsoever. Either choose a larger depiction or use different contrast, if this image is important for the story. Figure parts (d) and (e) are completely missing in the caption.

7. As already mentioned above, the part on page 22 on "miaslignement-tolerant, dynamic thickness measurements" is misleading as it is measure in transmission geometry and thus cannot be properly related to the reflection geometry shown in the other figures.

In conclusion, the manuscript presents a interesting and novel measurement method for characterizing total layer thicknesses with THz radiation using frequency comb stabilization. With the points above clarified the manuscript will be acceptable for publication in Nature Communications.

Reviewer #3

(Remarks to the Author)
I co-reviewed this manuscript with one of the reviewers who provided the listed reports. This is part of the Nature Communications initiative to facilitate training in peer review and to provide appropriate recognition for Early Career Researchers who co-review manuscripts.

The paper should not be published before the below concerns are addressed:

The authors report on an optical frequency-comb stabilized FDS THz spectrometer for inspection of lithium-ion battery electrodes. Through the frequency comb, which is stabilized to a Rb-clock, they achieve a high frequency-precision allowing for precise determination of Fabry-Perot-resonances of the electrode coatings, from which they extract the thicknesses and dielectric properties of the layers.
The topic of the manuscript is timely as battery production is increasingly important for various industries, especially electric cars. Here, THz non-destructive testing can provide significant advantages over other measurement techniques, such as X-ray or optical inspection.
The concept of measuring layer-thickness and material properties with a comb-stabilized THz FDS system as presented here is promising, however, the novelty of the work is limited, as various THz FDS measurement schemes for measurement of layer thickness and material-properties have been investigated and comb-stabilized spectroscopic THz-FDS measurements have also been demonstrated recently in the literature [1].
Furthermore, some of the claims made in the paper are questionable and the comparison to the literature could be stronger and more quantitative.
For the paper to be published in nature communications, the advantages of the system presented here should be made clearer and supported by quantitative comparisons. The following remarks should be addressed:

Introduction:
1. The authors claim that TDS systems cannot reach below micrometre precision. However, in ref. 27 sub-micrometre precision has been shown for a TDS system. Ref. 17, which is cited to support this claim is rather old and not representative of the state of the art.

2. Then, the authors argue that FDS systems also rely on time-delay measurements implying similar limitations as claimed for TDS systems. However, in ref. 18, the measurements are taken in the frequency domain and are only transformed into the time domain and evaluated there, which should not have a strong impact or follow the same limitations as the TDS systems, which rely on mechanical time delays. Furthermore, the precision reported in 18 is very similar to that reported by the authors in this work.

3. Furthermore, the authors claim, that other FDS systems require higher complexity due to calibration and look-up tables and are limited in measurement reliability. However, the use of a Rb-clock stabilized comb adds a significant amount of cost and complexity to the proposed system compared to the ones presented in refs. 18 and 27, which show a similar precision (down to below 50nm) and a strong stability over a long period of time. Additionally, there are works, achieving similar precision using only a few select frequencies, thus enabling very efficient systems with low complexity [2].

4. Therefore, it seems questionable if the results achieved in this work justify the use of this complex comb stabilization, which would make such a system rather big and expensive especially considering the use-case in industrial battery manufacturing. The authors should try to convince the reader of this. For example, are there any quantitative requirements regarding the precision in electrode measurements to maintain a certain yield or safety, etc, that only this system can provide? Or are there any direct relations between precision and yield that could justify the use of this system?

5. In general, a quantitative comparison to reported values in the literature (and possibly industry requirements) should be presented to support the claims made in this paper and strengthen the case for this approach.

6. The authors claim a two-orders-of-magnitude improvement over conventional methods; however, no quantitative numbers or references are provided to support this claim.

Measurement Setup:
1. Does the AOM introduce amplitude modulation as well? From experience, this could be imprinted on the PCA output as noise...

Results:
1. In the thickness measurements:
a. The anode shows higher precision despite the stronger absorption, which should reduce SNR and thus precision. The authors explain this by the thicker sample, which shifts the resonance towards lower frequencies where higher SNR is available, and by the reduced thickness deviations in the high-index materials. Regarding the former, this is not very intuitive

and a formula/simulation showing the SNR at the FP resonance as a function of thickness would help. Regarding the former, should the thickness deviations across samples not be independent of the precision of the measurement? And is this reduced deviation a direct consequence of the high index or just a coincidence? I think this finding should be discussed in more detail.

b. Regarding the SEM cross-sections inf Fig 3d, the authors state that the higher uncertainty in the SEM measurements stems from non-optimized sample preparation. Is that a common thing in industrial contexts for this type of measurement? Otherwise, the comparison of the two methods here would not be entirely fair, if better preparation is possible.

c. The authors claim sub-micrometre precision on their samples while admitting several hundreds of microns of roughness on the samples. This sounds contradictive. Unless the THz spot size is much smaller than the roughness, I do not see a way to make a measurement more precise than the roughness as the measurements would always be an average over the THz spot on the sample (which is later in the manuscript determined to be on the order of millimetres). Please explain.

d. In figure 3 and 4, there are some strong deviations between the thicknesses determined through THz and SEM, sometimes on the order of 20% and with the error-bars of the two measurements not overlapping at all. How do the authors explain this and what would be the ground truth then? A more detailed explanation on how the thickness is extracted from the SEM images would also be helpful.
2. In the material characterization section

a. The authors mention that the position of the dominant spectral dip changes with k. In figure 5, however, the dip appears to stay at the same frequency and only change depth and width over k. Please explain.

b. In figure 5d and S1d, which sample was used, A, B or C? Consider showing these plots for all three samples for the reader to get a better feeling of how these plots vary for different samples.

c. In figure 5c and s1c, consider plotting measurements and simulation in the same plot so the reader can evaluate the fit between measurement and simulation better.

d. Additionally, in fig 5c and s1c, the authors claim strong agreement between measurement and simulation. However, there are strong differences in the depth and width of the dips, and the FP frequencies also show deviations which could be on the order of 10% or more (difficult to tell exactly as the curves are in separate plots). This is particularly pronounced for fig. s1c. The origin of these deviations should be discussed in more detail.

3. In the thickness-profiling section:
a. The authors mention the extinction coefficient of the PLA is approximately 0.13. Where does this value come from? Is it measured as like the refractive index and if not, why not?

b. In figure 6e the authors claim excellent agreement between theory and measurement. While this is valid for the 1 Hz case, the 5 Hz measurements show some strong deviations in the oscillation amplitude. This could be discussed in more detail.

Discussion:
1. The authors claim unprecedented precision for the 68µm anode. This is hard to verify for the reader as no reference values are given from other works to compare this against. A table comparing the results of this work against the literature would be important to strengthen the case for this work. This table should include the measurement type, electrode type, material and thickness as well as the measurement precision. Furthermore, the authors only claim unprecedented precision for one specific sample. What about the others? If the precision is only strong for one sample, this doesn't make a strong case for the overall performance of the proposed system.

Figures
1. In Fig 2c the caption mentions a histogram which could not be found in the figure
2. In the caption of fig 6, the description of subfigures d and e is missing.
3. In figure S3a-c, consider unifying the styles and colors of the line. Are the samples considered for a, b and c the same?
4. In figure S3d, consider choosing a different unit for the entries with more than 2 or three decimals to improve readability.

[1] Krause, B., Müller, S., Puppe, T. et al. Terahertz photonic heterodyne spectral analysis with (sub-) kHz resolution and 6.5 THz frequency coverage. Nat Commun 16, 10594 (2025).
[2] Schwenson, L., Walter, F., Jäckel, A. et al. Sparse Terahertz Frequency-Domain Sensing with Kilohertz Measurement Rate. J Infrared Milli Terahz Waves 46, 52 (2025).

Reviewer #4

(Remarks to the Author)
I co-reviewed this manuscript with one of the reviewers who provided the listed reports. This is part of the Nature Communications initiative to facilitate training in peer review and to provide appropriate recognition for Early Career Researchers who co-review manuscripts.

Version 1:

Reviewer comments:

Reviewer #1

(Remarks to the Author)
I am satisfied with the authors' revisions. They have addressed all of my previous concerns thoroughly and thoughtfully. The manuscript is now clear, well-argued, and scientifically robust. I recommend acceptance.

Reviewer #2

(Remarks to the Author)
The authors provide a very nicely revised version of the initial manuscript. All the points raised during the first round of review have sufficiently been addressed and many important details have been added to the manuscript, especially a proper comparison to the state of the art with related technologies. Thus, I see the manuscript now fir for publication and congratulate the authors on this beautiful work.

Reviewer #3

(Remarks to the Author)
The authors have addressed all my comments and I appreciate the extensive revisions made. Only one point remains regarding the comparison with SEM-based measurements:
In their response to my comment #11, the authors describe that both methods, THz and SEM, are fundamentally different and that the differences in measurement stem from the sample preparation, porosity and measurement location, which was different between THz and SEM.
This raises several questions. What is important to be measured in this use-case? Only the layer thickness, or is the porosity also important to be monitored? If porosity is important, too, can it be extracted from THz measurements?
And for the thickness, is the absolute thickness including porosity important or only the solid thickness? And can the porosity be considered in the THz measurements to get the correct thickness?
Lastly, regarding the methodology. Although SEM can of course only access cross-sections, while THz averages over an area, I think it would be better for the comparison to have both measurements taken on the same site. Why was it not possible to do the THz measurement first and then perform the destructive SEM analysis?
I think, discussing these points would clarify the benefits of the proposed method and strengthen the paper.
Upon addressing these points, I can recommend the paper for publication.

Reviewer #4

(Remarks to the Author)
I co-reviewed this manuscript with one of the reviewers who provided the listed reports. This is part of the Nature Communications initiative to facilitate training in peer review and to provide appropriate recognition for Early Career Researchers who co-review manuscripts.

Version 2:

Reviewer comments:

Reviewer #3

(Remarks to the Author)
All my concerns have now been addressed and I can recommend acceptance

Reviewer #4

(Remarks to the Author)
I co-reviewed this manuscript with one of the reviewers who provided the listed reports. This is part of the Nature Communications initiative to facilitate training in peer review and to provide appropriate recognition for Early Career Researchers who co-review manuscripts.

Open Access This Peer Review File is licensed under a Creative Commons Attribution 4.0 International License, which permits use, sharing, adaptation, distribution and reproduction in any medium or format, as long as you give appropriate credit to the original author(s) and the source, provide a link to the Creative Commons license, and indicate if changes were made.
In cases where reviewers are anonymous, credit should be given to 'Anonymous Referee' and the source.
The images or other third party material in this Peer Review File are included in the article's Creative Commons license, unless indicated otherwise in a credit line to the material. If material is not included in the article's Creative Commons license and your intended use is not permitted by statutory regulation or exceeds the permitted use, you will need to obtain permission directly from the copyright holder.
To view a copy of this license, visit https://creativecommons.org/licenses/by/4.0/

Young-Jin KIM
Professor of Department of Mechanical Engineering,
Korea Advanced Institute of Science and Technology (KAIST)
291 Daehark-ro, Yuseong-gu, Daejeon 34141, Republic of Korea
E-mail: yj.kim@kaist.ac.kr, Tel: +82 42-350-3017, Mobile: +82 10-7613-2893

24th February 2026

Dear Reviewers,

We are submitting our manuscript entitled "**Nanometre-precision terahertz interferometry for battery electrode metrology**" that has been revised to address all the comments raised by reviewers.

The authors appreciate the reviewer's valuable time and efforts for going through the manuscript and making constructive comments on it. The manuscript has been carefully revised according to the reviewer's comments and the point-to-point responses for all the comments are discussed below (Reviewers' remarks are reproduced in black while our responses are summarized in blue). We hope that the revised manuscript successfully addresses all the comments and concerns of the editor and reviewers.

Thank you for your time and consideration.

Best regards,

Young-Jin Kim, Ph.D.

Professor of Department of Mechanical Engineering,

Korea Advanced Institute of Science and Technology (KAIST),

291 Daehark-ro, Yuseong-gu, Daejeon 34141, Republic of Korea

E-mail: yj.kim@kaist.ac.kr
Tel: +82-42-350-3017

[Author's responses to Reviewer #1]

This manuscript presents a novel and highly impactful approach to non-contact, nanometre-precision thickness measurement of lithium-ion battery (LIB) electrodes using frequency-comb-referenced terahertz (THz) Fabry-Perot (FP) interferometry. **The work is timely, well-executed, and addresses a critical metrology gap in advanced battery manufacturing, particularly for solid-state and high-throughput production lines.** The integration of an optical frequency comb for SI-traceable frequency calibration, combined with the inherent penetration and scattering resistance of THz radiation, enables unprecedented precision (e.g., 7.8 nm for anodes at 25.6 s integration) and high measurement speeds (>12 THz/s). The study is thorough, with systematic validation across different electrode materials, thicknesses, 3D profiling, and dynamic measurements. **The manuscript is well-organized, the figures are clear, and the writing is generally fluent.**

I recommend acceptance after minor revisions.

While the manuscript is strong, the following points could enhance its clarity, depth, and impact:

Comment 1. Material property generalizability:
The complex refractive indices used for cathodes (2.9+0.5i) and anodes (6.0+2.0i) are derived from specific samples. Please comment on how these values may vary with electrode composition, porosity, or manufacturing process, and how such variations could affect measurement accuracy in industrial settings.

Response 1: We appreciate the reviewer's comment.

The complex refractive index is expressed as $n+i\kappa$, where n is the refractive index and κ is the extinction coefficient. Electrode composition primarily affects κ—higher conductive filler content (e.g., carbon black, graphite) increases κ, explaining why anodes exhibit larger κ values than cathodes. Electrode porosity reduces the effective refractive index n due to increased air volume fraction, following effective medium theories [Ref. A]. The manufacturing process determines the resultant porosity: conventional wet coating yields 30–50% porosity [Ref. 1], whereas emerging dry electrode processes for solid-state batteries produce denser, more uniform structures.

Regarding measurement accuracy, since $t \propto 1/n$ in **Eq. (2)**, the relative uncertainty in refractive index n directly translates to the relative uncertainty in thickness t. This challenge can be addressed by (1) establishing material-specific calibration databases, or (2) utilizing our simultaneous material characterization capability (as demonstrated in **Fig. 5**) to determine the complex refractive index in situ without prior calibration.

Following the reviewer's comment, we have added a brief discussion in the revised manuscript:

(on page 19, line 415)

> *"... However, XCORR values remain broadly high across a range—for example, from 2.5+0.2i to 3.3+1.0i—indicating limitations in resolving unique optical constants (**Fig. 5d**). Since the complex refractive index n+iκ varies with electrode composition, porosity, and manufacturing process, in situ characterization offers significant advantages over relying on pre-established values. To address this, polarization-resolved reflection spectroscopy can be employed. ..."*

Reference:

[Ref. A] G. A. Niklasson, C. G. Granqvist, and O. Hunderi, "Effective medium models for the optical properties of inhomogeneous materials," Appl. Opt. 20, 26-30 (1981)

Comment 2. Performance limits for ultra-thin electrodes (<50 μm):
The study notes reduced precision for thinner samples due to broader FP modes. Could the authors briefly (1) suggest potential strategies to improve fringe visibility in such cases (e.g., enhanced reflection layers, multi-mode analysis) or (2) define the practical lower thickness limit of the method?

Response 2: We appreciate the reviewer's comment.

Following the reviewer's comment, we have supplemented in-depth discussion about the visibility improvement strategy and lower thickness limit as following.

(1) As sample thickness decreases, the number of observable FP modes reduces and the FP dips become broader, limiting the precision in determining the center frequency. To address this limitation, introducing additional reflecting layers can strengthen interference contrast. As illustrated in **Fig. R1** (using ultra-thin silicon sample as an example), placing the thin sample on a carrier substrate (e.g., a silicon wafer with well-defined refractive index and thickness) with an air gap generates rich spectral content with multiple FP modes. The envelope of the combined spectrum carries thickness information of the thin sample. To extract thickness from such multi-layer configurations, spectral fitting methods combining the transfer-matrix method (TMM) with our frequency-comb-referenced measurements can be employed—a strategy we plan to explore in our forthcoming investigations.

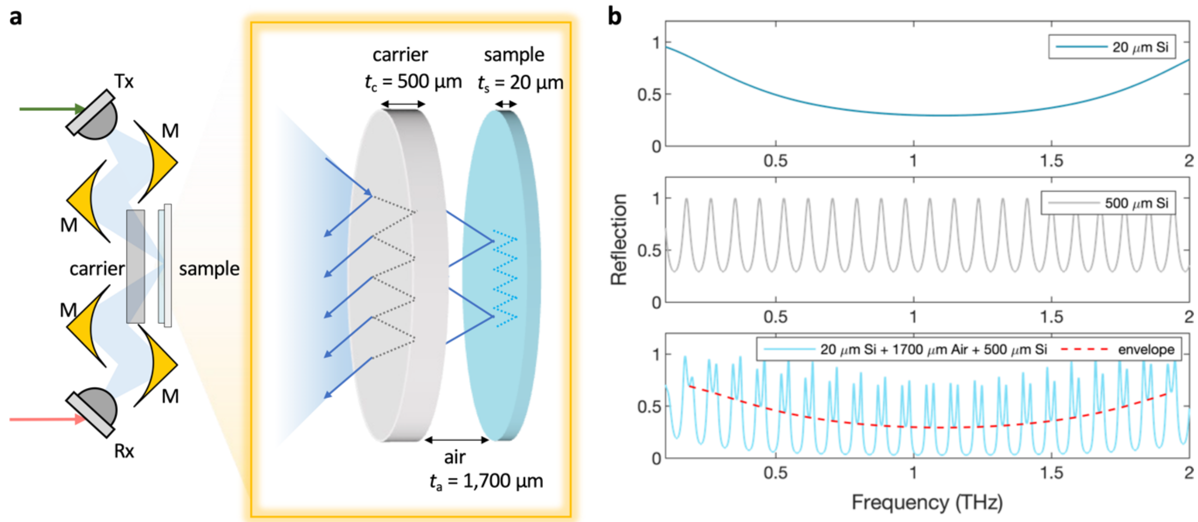

Fig. R1. Enhanced interference contrast through additional reflecting layers for thin sample thickness determination. a Schematic of the reflection measurement setup with multi-layer configuration. The right inset illustrates the carrier-air-sample assembly: a carrier substrate (t_c = 500 μm), an air gap (t_a = 1,700 μm), and a thin sample (t_s = 20 μm). **b** Simulated reflection spectra for three configurations: a 20 μm thin Si sample alone (top), a 500 μm Si carrier alone (middle), and the combined multi-layer structure (bottom). The combined configuration generates rich spectral content with well-defined interference fringes (cyan curve), and the envelope (red dashed curve) carries thickness information of the thin sample, enabling thickness determination of ultrathin electrodes.

Following the reviewer's comment, we have supplemented this discussion in the revised manuscript:

(on page 14, line 294)

"... by introducing additional reflecting layers—such as a well-defined silicon substrate and air gap above the thin sample—to generate supplementary spectral content and strengthen interference contrast ..."

(2) The practical lower thickness limit is determined by the requirement that the Fabry-Pérot (FP) dip must be sufficiently observable within the measurement bandwidth (0.1–1.0 THz) for accurate center frequency determination. Reliable Lorentzian fitting requires at least half of the full width at half maximum (FWHM) to be visible within the measurement range.

The FWHM is governed by the free spectral range (FSR) and finesse F: FWHM = FSR/F = $c/(2ntF)$, where the finesse is determined by Fresnel reflectance as $F = \pi R^{1/2}/(1-R)$. For the first destructive mode $f_{m=0.5}$ and half of its FWHM to remain below f_{max} = 1.0 THz: $t_{min} > c/(4nf_{max}) * (1+1/F)$.

Using the refractive indices determined in this study, the minimum measurable thicknesses are calculated as **Table R1**. The anode exhibits a lower thickness limit due to its higher refractive index, which shifts FP modes to lower frequencies, and higher finesse, which produces sharper dips with narrower FWHM.

Table R1. Practical lower thickness limits for LIB electrodes determined by FP mode observability within the 0.1–1.0 THz measurement bandwidth.

Parameter	Refractive index n	Fresnel reflectance R	Finesse F	Minimum thickness t_{min}
**Cathode**	2.9	0.25	2.1	~38 μm
**Anode**	6.0	0.55	5.2	~15 μm

Comment 3. Lateral resolution in imaging applications:
The lateral resolution is limited to ~2 mm due to the long THz wavelength. Although deep-learning super-resolution is mentioned as future work, a short discussion on the implications of this limitation for in-line inspection of fine electrode features (e.g., coating edges, micro-defects) would be helpful.

Response 3: We appreciate the reviewer's suggestion.

The ~2 mm lateral resolution presents different implications depending on the inspection target. For coating edge profiling, our spectral analysis approach can effectively characterize multi-layer thickness transitions encoded in FP mode frequencies, as demonstrated in **Fig. 5**. However, for micrometre-scale defects, the millimetre-scale beam diameter provides spatially averaged measurements, limiting sensitivity to localized micro-defects. Future advances in deep-learning-based super-resolution and structured illumination using THz-compatible spatial light modulators (SLMs) could extend applicability to micro-defect inspection.

Following the reviewer's suggestion, we have included further discussion about the future potential of the deep-learning in our work as follows:

(on page 22, line 486)

" This approach also demonstrated lateral resolution of ~2 mm, consistent with the Rayleigh diffraction limit. This resolution is well-suited for characterizing coating edge profiles, where multi-layer thickness transitions are encoded in spectral FP modes. However, detection of micrometre-scale lateral defects remains challenging due to spatial averaging over the

millimetre-scale beam diameter. Future work may explore deep-learning-based super-resolution techniques, which have demonstrated sub-diffraction-limit imaging[45,46] (~λ/4), as well as structured illumination approaches using THz-compatible spatial light modulators[47], to surpass this limit and extend applicability to micro-defect inspection."

Comment 4. Clarity in uncertainty reporting:
The uncertainty analysis is detailed in Supplementary Fig. 3 but only briefly summarized in the main text. I recommend adding a concise summary of the key uncertainty sources (sample geometry, refractive index variation, SEM preparation artifacts) and their magnitudes in the Results or Methods section.

Response 4: We appreciate the reviewer's constructive suggestion.

We agree that a concise summary of key uncertainty sources would improve clarity for readers.

Following the reviewer's recommendation, we have made the following revisions:

(on page 15, line 322)

*"... A detailed uncertainty analysis (**Supplementary Fig. 4**) reveals three key uncertainty sources: sample geometry (~3.8 μm from surface roughness, flatness, and edge rounding), refractive index variation for THz interferometry, and ion milling artifacts for SEM imaging. The overall measurement uncertainties are estimated to be ~0.1 μm for THz interferometry—dominated by variations in the complex refractive index—and ~4.3 μm for cross-sectional SEM imaging—primarily due to sample damage and delamination during ion milling (see **Methods** for details)."*

Comment 5. Comparative table with existing techniques:
A concise table comparing the proposed method with XCT, SAM, LDS, and conventional THz-TDS/FDS in terms of resolution, speed, penetration, material compatibility, and cost would provide readers with a quick overview of the advantages. The conclusion states that the precision is improved by two orders of magnitude compared with similar methods relying on temporal analysis, but the following are not clarified. The specific type of "traditional method" used for comparison (e.g., a certain brand of laser displacement sensor, a specific X-ray system) and its experimental conditions (e.g., integration time, sample thickness).

Response 5: We appreciate the reviewer's constructive suggestion.

Following the reviewer's comment, we have prepared a comprehensive comparison table (**Table R2**) summarizing the key performance parameters of existing non-destructive inspection techniques for battery electrode metrology, including representative commercial equipment and literature references. As shown in **Table R2**, our THz-FP-FDS achieves 70 nm precision for anodes—approximately one order of magnitude improvement over conventional THz-FDS (~180 nm) and LDS (~760 nm). Additionally, our approach offers unique advantages of SI-traceable accuracy and calibration-free operation, which are essential for reliable process development.

Table R2. Comparative analysis of thickness measurement techniques: XCT, SAM, LDS, and THz-based methods for LIB electrode inspection.

Parameter	XCT	SAM	LDS	THz-TDS	THz-FDS	THz-FP-FDS (This work)
**Thickness precision (single)**	N/A	N/A	1,270 nm (anode) 760 nm (cathode)	~200 nm (dielectrics)	270 nm (anode) 180 nm (cathode)	70 nm (anode) 466 nm (cathode)
**Lateral resolution**	0.5-50 μm	40-150 μm	N/A	N/A	N/A	~2 mm
**Measurement rate**	0.0003-0.03 scans/s	1,000-2,000 points/s	83 mm/s (In-line)	8~15 spectra/s	68-200 spectra/s	5 spectra/s
**Penetration depth**	mm-cm (Full cell)	Sub-mm (4 layers)	N/A (Surface only)	Several-mm (dependent on material composition)		
**Key advantages**	Full 3D internal structure, Defect detection	Layer-by-layer imaging, Delamination detection	High-speed inline, Simple operation	Broadband, Material characterization	Compact, Fast sweep, Lower cost	Calibration-free, SI-traceable, nm-precision
**Key limitations**	Slow acquisition, High cost, Radiation safety	Limited penetration, Coupling medium required	Surface-only, Scattering on rough surfaces	Limited lateral-resolution (mm-scale)		
**Representative equipment**	ZEISS, Xradia 620 Versa	PVA TePla, SAM 500 HD	KEYENCE, laser caliper	Toptica, TeraFlash	TOPTICA, T-Sweeper	N/A
**Key references**	Pietsch & Wood (2017) [Ref. 7]	Bauermann (2023) [Ref. 8]	Mohanty (2014) [Ref. 9]	Liebermeister (2021) [Ref. 19]	Mohammadzadeh (2023) [Ref. 22]	This work

Following the reviewer's comment, we have revised the Introduction to include quantitative performance parameters for each conventional technique.

(on page 3, line 46)

> *"X-ray computed tomography (XCT) offers deep penetration and sub-micrometre voxel sizes capable of resolving internal structures and defects such as voids or misalignments, but its slow acquisition speed of minutes and high cost hinder practical deployment in high-throughput production lines[7]. Scanning acoustic microscopy (SAM) enables non-destructive subsurface probing by detecting internal acoustic reflections, but its lateral resolution is limited to tens of micrometres by long acoustic wavelengths, thickness precision typically remains at the micrometre level[8,10]. …"*

Regarding the precision improvement claim, we have revised the manuscript to provide specific quantitative comparisons with references. The original "two orders of magnitude improvement" has been clarified as "one-to-two orders of magnitude improvement" with explicit context for battery electrode metrology:

(on page 2, line 25)

> *"...confirming one-to-two orders of magnitude improvement over the counterparts relying on temporal analysis for battery electrode metrology."*

(on page 5, line 105)

> *"...demonstrating an order-of-magnitude enhancement in single-shot measurements and up to two orders of magnitude with averaging compared to conventional temporal-analysis-based methods for battery electrode metrology."*

Comment 6. Verification in Actual Electrode Production Environments:
While the manuscript compellingly demonstrates the technical capabilities of THz FP interferometry in a controlled laboratory setting, its practical impact would be significantly strengthened by addressing real-world manufacturing challenges more directly (e.g., continuous operation stability and environmental factors (vibration, dust, humidity)). It is recommended to include a dedicated section discussing the path toward industrial deployment.

Response 6: We appreciate the reviewer's constructive suggestion.

We have addressed industrial deployment through two complementary scenarios: (1) as a stand-alone, reference-grade metrology tool for initial process development, where throughput constraints are not a primary concern, and (2) for in-line quality control, where the Rb-clock-stabilized frequency comb ensures long-term reproducibility, and the long THz wavelength with common-path configuration provides inherent robustness against vibrations and misalignments. Dust is inherently controlled in battery manufacturing environments to prevent internal short circuits. Regarding humidity, water vapor absorption lines (557, 752, 988 GHz) can affect measurement precision. For high-precision measurements with extended integration times, humidity suppression via dry-air purging (RH $< 5\%$) is recommended, whereas typical ambient humidity levels are acceptable for routine single-shot measurements.

Following the reviewer's suggestion, we have revised the manuscript as follows:

(on page 19, line 419)

> *"This capability to simultaneously determine thickness and material properties positions the system as a stand-alone, reference-grade metrology tool for initial process development, where establishing optimal electrode coating conditions requires precise characterization as functions of process parameters such as slurry composition, coating speed, and drying temperature—without the throughput constraints imposed by production-line conveyor speeds."*

(on page 23, line 505)

> *"These results highlight the capability of our THz FP interferometry to perform precise, real-time thickness measurements even under rapid, dynamic conditions. Combined with the Rb-clock-stabilized frequency comb ensuring long-term measurement reproducibility without periodic recalibration, and the inherent robustness of long THz wavelengths and common-path configuration against mechanical vibrations and angular misalignments, our system reduces the need for elaborate vibration isolation setups and precise optical alignment typically required in NIR-based interferometric systems[48]. These features collectively fulfill key requirements for next-generation solid-state battery manufacturing and position our THz FP interferometry as a practical solution for future in-line quality control."*

(on page 26, line 590)

> *"In order to minimize spectral artifacts from water vapor absorption lines, all measurements were performed inside a dry-air purge enclosure maintained at a relative humidity below 5%."*

Minor editorial suggestions:

Comment 7. P. 5-6, line 110-111: The transition "This capability establishes…" feels slightly abrupt. Consider a smoother link to the preceding sentence.

Response 7: We appreciate the reviewer's suggestion.

Following the reviewer's comment, we have revised the manuscript to provide a smoother transition:

(on page 6, line 111)

> *"... This combination of nanometre-precision metrology, dynamic monitoring, and material characterization establishes a unified, calibration-free platform for high-throughput, high-precision LIB electrode metrology, directly addressing the emerging demands of battery manufacturing."*

Comment 8. Some figure references in the text (e.g., description of Fig. 2a) could be more precisely aligned with the visual elements.

Response 8: We appreciate the reviewer's suggestion.

Following the reviewer's comment, we have revised the description of **Fig. 2a** to more precisely align the text with the visual elements shown in the figure.

(on page 9, line 182)

> ***" Frequency-sweeping THz nonlinearity compensation. Figure 2a*** *illustrates the concept of a comb-based calibration scheme employed to ensure absolute frequency accuracy and precision in the THz spectroscopy. An Er-doped fibre frequency comb, referenced to an SI-traceable Rb atomic clock, provides the absolute frequency grid for calibration. The fixed laser* v_1 *is set at 1565 nm, while the swept laser* $v_2(t)$ *sweeps from 1564 nm to 1556 nm over time. The frequency difference* $v_2(t)$-v_1 *generates a continuously sweeping THz output* $f_{THz}(t)$ *in the range of 0.05–1.05 THz within 0.2 s via optical-to-THz down-conversion."*

Comment 9. Ensure consistent use of "LIB" vs. "Li-ion battery" throughout.

Response 9: We appreciate the reviewer's suggestion.

Following the reviewer's comment, we have ensured consistent use of "LIB" throughout the manuscript.

[Author's responses to Reviewer #2]

The authors report on an optical-frequency-comb stabilized THz measurement technique to determine the layer thickness of battery electrodes with a precision down into the few nanometer range. This increased precision is reached due to the frequency stabilization which allows for the exact calculation of the layer thickness using the absolute position of the Fabry-Perot fringes generated due to multiple reflections inside the layer. Thus, this method works reliably only if the materials have high enough transparency in comparison to the skin depth. For the battery materials under investigation this is true for the low THz frequency range up to 1 THz.

While in TDS systems interferometric stabilization has already been introduced some time ago, using a frequency comb for stabilization is a novel approach, especially allowing for very fast frequency sweeps as demonstrated in this manuscript. The precision reached for 0.2s integration time is 70.1 nm for the anode, which is usually the more challenging electrode to be characterized. **Increasing the integration time to 25.6 s, the precision is reported ot fall down to 7.8 nm - a truly impressive value compared to the commonly reached precision of a few 100 nm using commercial TDS systems.**

In conclusion, the manuscript presents a interesting and novel measurement method for characterizing total layer thicknesses with THz radiation using frequency comb stabilization. With the points above clarified the manuscript will be acceptable for publication in Nature Communications.

A few points need further clarification prior to a possible publication:

Comment 1. For industrial inspection, (1) 25.6 s integration time per measurement point will be prohibitive, as fabrication speeds of battery electrodes reach meters/second but even 70 nm are perfectly in range of the industrial requirements. (2) What is the reason that the anode data surpass the cathode data although the anode usually is more absorbing? Please, clarify.

Response 1: We appreciate the reviewer's insightful comment.

(1) We agree that 25.6 s integration time would be prohibitive for high-speed in-line inspection. However, as the reviewer noted, 70-nm precision at 0.2 s integration is well within industrial requirements. Our system is particularly suited as a stand-alone, reference-grade metrology tool for initial process development, where establishing optimal coating conditions requires precise characterization without production-line throughput constraints. In this context, extended integration times can be fully utilized for process optimization before transitioning to conventional in-line sensors for mass production.

Following the reviewer's comment, we have supplemented detailed discussion about the measurement speed requirement in the initial process optimization in the revised manuscript as follows:

(on page 14, line 296)

> *"...cases B and C successfully achieved sub-100 nm precision at 8.4 s and 3.8 s, respectively, and further improved to 63.4 nm and 25.9 nm at 25.6 s integration—demonstrating capability as a reference-grade metrology tool for initial process optimization—while sub-micrometre*

precision at 0.2 s supports applications requiring higher throughput. These results confirm the robustness of the system for monitoring the thickness of LIB electrodes."

(2) The higher precision observed in anodes, despite their stronger absorption, can be explained by the relationship between optical path difference (OPD) and FP mode frequency position. As shown in **Table R3**, anodes have a higher refractive index ($n \approx 6.0$) compared to cathodes ($n \approx 2.9$), resulting in a larger OPD ($= 2nt\cos\theta_t$) for the same physical thickness. This shifts the FP modes to lower frequencies, where our THz source provides higher SNR and thus better frequency precision.

Table R3. Comparison of optical path difference and FP mode frequency for cathode and anode samples.

Sample type	Norm. thickness t_{nom} (μm)	Refractive index n	Meas. thickness t_{THz} (μm)	Meas. OPD OPD (μm)	FP mode $f_{m=0.5}$ (THz)	Thickness precision δt_{THz} (nm) at 0.2-s
Cathode	A (50 μm)	2.9	45.1 μm	130.8 μm	0.591 THz	602.7 nm
	B (70 μm)		50.9 μm	147.6 μm	0.523 THz	465.5 nm
	C (90 μm)		105.2 μm	305.1 μm	0.253 THz	286.7 nm
Anode	A (50 μm)	6	47.0 μm	282.0 μm	0.264 THz	171.6 nm
	B (100 μm)		103.4 μm	620.4 μm	0.120 THz	70.1 nm
	C (150 μm)		170.3 μm	1021.8 μm	0.073 THz	174.5 nm

According to the precision analysis presented in **Fig. S3**, FP modes at lower frequencies yield superior frequency precision owing to the higher SNR in this spectral region. This directly translates to improved thickness precision for anodes. The outlier behavior of Anode C (degraded precision despite the lowest frequency) is attributed to the extremely low FP mode frequency (~0.09 THz), where the swept laser exhibits large acceleration (as shown in **Fig. 2b**, **c**) and optical power transmission through the aperture is reduced, as discussed in the manuscript.

Following the reviewer's comment, we have revised the manuscript and added a detailed precision analysis:

(on page 13, line 282)

*"... The standard deviation σ_t decreases with increasing thickness, reflecting enhanced frequency stability at lower FP mode frequencies (see **Methods** for details). ..."*

(on page 16, line 350)

*"... Second, the laser frequency sweep accelerates in this low-frequency region, and although frequency comb calibration is applied, residual nonlinearity can adversely affect the accuracy of FP mode detection (see **Methods** for details)."*

(on page 26, line 595)

*"**Precision analysis of each FP mode.** To evaluate the precision characteristics of each FP mode, we analysed the frequency and thickness precision as a function of mode number (i.e., frequency position) based on measurements of a silicon wafer. **Supplementary Fig. 3a** shows the transmission amplitude spectrum of a 525-μm thick silicon wafer, where eleven destructive FP modes ($f_{m=1.5}$ to $f_{m=11.5}$) are observed across the 0.1–1.0 THz range. As frequency increases, the SNR decreases, leading to degraded frequency precision (**Supplementary Fig. 3b**). ..."*

(on page 35, line 818)

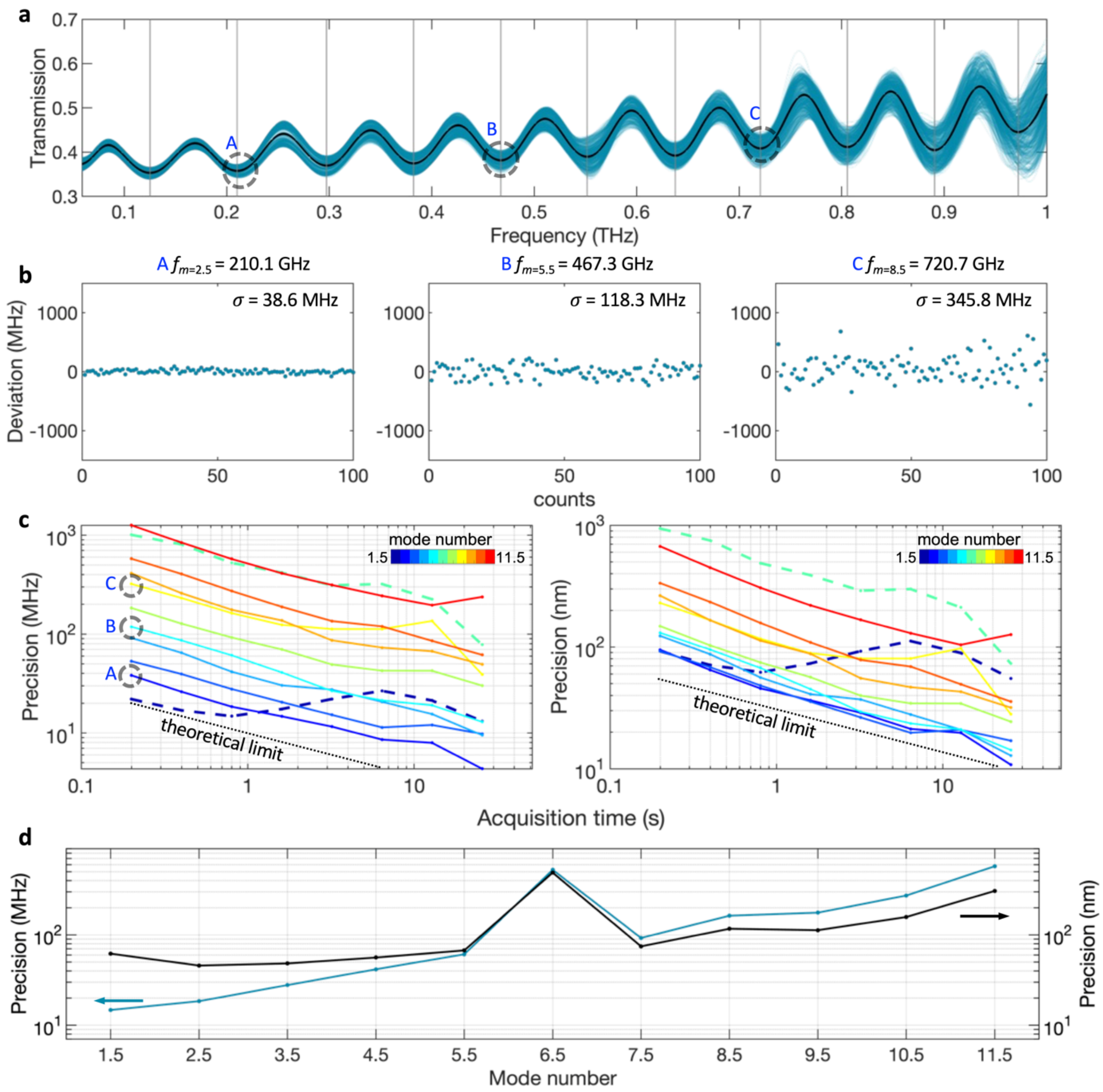

Fig. S3. Precision analysis of frequency and thickness measurements as a function of FP mode number. a *Transmission amplitude spectra of a 525-µm thick silicon wafer measured over 1,000 repeated acquisitions across the 0.1–1.0 THz range. The black solid line represents the averaged spectrum, and grey vertical lines indicate the centre positions of destructive FP modes.* ***b*** *Deviation of FP mode centre frequencies for three different FP modes (Case A: $f_{m=2.5}$ = 210.1 GHz, Case B: $f_{m=5.5}$ = 467.3 GHz, Case C: $f_{m=8.5}$ = 720.7 GHz).* ***c*** *Allan deviation of FP mode frequency precision (left panel) and the retrieved thickness precision (right panel).* ***d*** *Frequency precision (blue, left y-axis) and thickness precision (black, right y-axis) as a function of mode number at 0.8-s acquisition time. The thickness precision shows a different behaviour compared to frequency precision due to the inverse dependence on mode number m.*

Comment 2. In Fig. 1 the measurement setup ist presented. Here, <u>(1) it is obvious that the samples need to be measured in reflectance geometry due to the opaque metal collector foil. Thus, it is not clear, how this relates to the angle dependent measurements shown in Fig. 6, in which clearly a geometry in transmission is required.</u> This change in experimental geometry is not mentioned in the main text - or I have overlooked it. Especially in reflection geometry angles as large as the stated 45° will be prohibitive. Thus, the whole section on the angle dependent measurement needs clarification. Furthermore, the

authors nicely demonstrate that they can measure under different angles and they identify the resulting variation in thickness. (2) For inspection, the effective thickness is not important but the real thickness. How can the real thickness be retrieved without knowing (and measuring) the angle, under which the data have been taken? (3) In Fig. 6 the caption stops after figure part (c). The angle dependent parts seem to have been added later. They are thus not found in the caption and need to be included.

Response 2: We appreciate the reviewer's careful observation.

(1) The transmission geometry with the tilted quartz wafer in **Fig. 6d–e** was chosen as the simplest configuration to demonstrate fast and precise dynamic thickness measurements enabled by our comb-referenced approach.

The primary contribution of this work is the frequency comb referencing technique for high-precision thickness determination, rather than a specific optical configuration. The THz optical system can be adapted to various geometries—transmission, reflection at normal incidence, or oblique incidence (e.g., 8° or 45°)—depending on the application requirements. The governing equations, **Equations (1) and (2)**, describe the universal relationship between FP mode frequencies and sample thickness, which remains valid across all these configurations.

For practical in-line inspection of LIB electrodes, a compact reflection head with co-aligned emitter and receiver (~8° incidence angle) would be preferable. Our stand-alone system employs a 45° incidence reflection geometry, which offers a distinct advantage: it enables polarization-resolved spectroscopy. At oblique incidence, s- and p-polarized light exhibit different Fresnel transmission and reflection coefficients, which manifest as distinct spectral features in the measured spectra. By analyzing both polarization channels, additional constraints can be imposed on the determination of complex refractive index, improving the accuracy of material characterization beyond what is achievable with normal-incidence measurements alone. This capability also opens pathways toward THz ellipsometry for comprehensive, non-destructive material analysis in battery electrode metrology. Following the reviewer's comment, we have revised the manuscript to clarify the applicability of our approach to various optical configurations:

(on page 23, line 496)

> *"... This robustness ensures reliable thickness measurements despite significant angular deviations, making the technique suitable for integration into high-speed manufacturing environments where perfect alignment cannot always be maintained. Our frequency-comb-referenced THz source can be combined with various optical configurations, with **Equations (1)** and **(2)** remaining universally applicable. To demonstrate the fast and precise dynamic measurement capability, we employed a transmission geometry with a tilted quartz wafer as the simplest experimental configuration. We implemented a setup using a fast-steering mirror (FSM; OIM102, Optics in Motion) to periodically modulate the incidence angle θ_i, as depicted in **Fig. 6d**. ..."*

(2) The incidence angle is defined during system configuration and validated through periodic calibration with reference samples, enabling reliable real thickness extraction via **Eq. (2)**.

(3) Following the reviewer's comment, we have revised the Fig. 6 title and caption to include descriptions of parts **d** and **e**:

(on page 21, line 448)

*"**Fig. 6. THz FP mode monitoring for thickness profiling and dynamic measurements.** ... **d** Schematic of the dynamic thickness measurement setup, with the right-top inset illustrating angle modulation of the fast steering mirror for effective optical path length modulation. **e** Measured effective thickness modulations fitted with a sinusoidal curve, along with its corresponding Fourier transform. M: off-axis parabolic mirror, FSM: fast-steering mirror, VCO: voltage-controlled oscillator."*

Comment 3. In Fig. 1 f, the notion (a.u.) needs to be removed as normalized data are shown. Due to the normalization all units are normalized out so that indeed only numbers without units are left. This applies for all other normalized plots also.

Response 3: We appreciate the reviewer's comment.

Following the reviewer's suggestion, we have removed "(a.u.)" from all normalized plots. The revised figures are: **Fig. 1f**, **Fig. 2d**, **Fig. 3a**, **Fig. 4a**, **Fig. 5a–c**, **Fig. 6a** and **Fig. S1a-c**.

Comment 4. In Fig. 2 the Allan deviation is plotted, very convincingly showing the improvement due the frequency comb stabilization. However, (1) the true benefit starts from integration times higher than several seconds, which - to our experience - is way too long for industrial inspection. Additionally, (2) what is the tolerance requested by industry for the electrode thickness? A few nanometers really asked for?

Response 4: We appreciate the reviewer's practical perspective on industrial requirements.

(1) We envision our system as a stand-alone, reference-grade metrology tool for initial process development, where extended integration times are acceptable to establish robust process-thickness correlations. Once the process is stabilized, thickness monitoring under controlled conditions can be performed with single-shot measurements at 0.2 s, achieving sub-micrometre precision. Furthermore, by narrowing the frequency sweep range (e.g., 0.1–0.3 THz), even faster acquisition is achievable for high-throughput applications.

(2) As standardized metrology methods for electrode thickness have not yet been established, sub-micrometre precision is generally required for electrodes with thicknesses of several tens of micrometres. In this work, we aimed to explore and demonstrate the precision limits achievable through frequency comb referencing. Beyond precision improvement, the comb-referenced reproducibility [Ref. 24]—achieved without periodic recalibration—is essential for establishing reliable process-thickness correlations in industrial process development.

Following the reviewer's comment, we have added a clarification in the revised manuscript:

(on page 11, line 247)

"... This demonstrates the superior long-term stability and precision provided by the OFC stabilized to an Rb atomic clock, enabling nanometre-scale thickness precision in transparent materials such as silicon[36]. Furthermore, such comb-referenced reproducibility[24], achieved

without periodic recalibration, is essential for establishing reliable process-thickness correlations in industrial process development.

Comment 5. <u>(1) Fig. 3: The idea to use the same coloration in (d) than in (e) is very nice, but in this case the color chosen for t_SEM=99 µm needs to be adjusted either in (d) or in (e) to really match the image.</u> In figure (e) the legend for t_THz would then also need an adjustment, e.g. include threee colors into the box. It would also help to <u>(2) state the accquisition time needed to retrieve the data shown in (a) and (b)</u> (same for Fig. 4) already in the figure caption. <u>(3) In (b) the long term stability is demonstrated, showing several measurements over a larger stretch of time, correct?</u>

Response 5: We appreciate the reviewer's careful observation and constructive suggestions.

(1) Following the reviewer's suggestion, we have adjusted the color for Case C in **Fig. 3d** to match the color scheme used in **Fig. 3e**, ensuring visual consistency across the figure.

(on page 13, line 269)

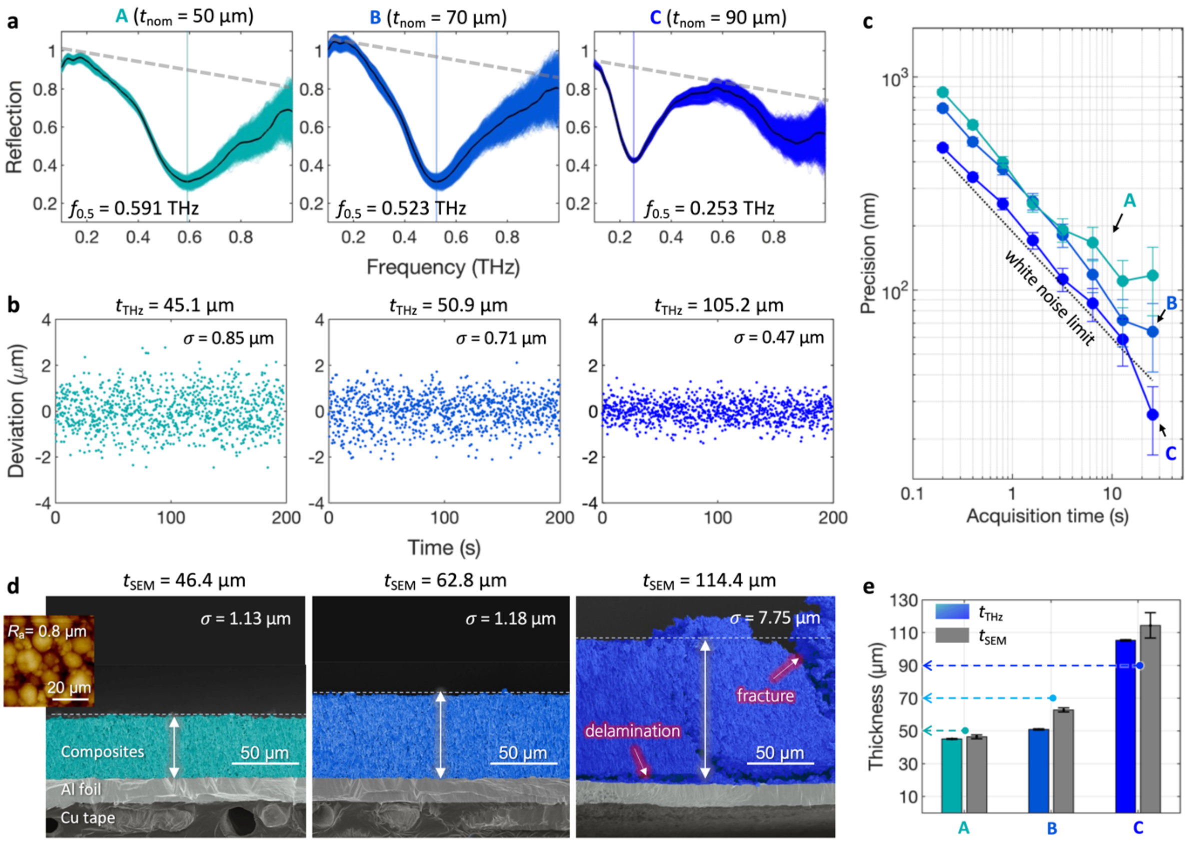

Fig. 3. Measurement precision analysis of LIB cathodes. …

(2) Following the reviewer's suggestion, we have added the acquisition time information to the captions of **Fig. 3** and **Fig. 4**:

(on page 13, line 269)

*"**Fig. 3**. **Measurement precision analysis of LIB cathodes. a** Measured reflection amplitude spectra for three nominal thicknesses (Case A, 50 μm; Case B, 70 μm; Case C, 90 μm), acquired with a period of 0.2 s over 200 s, with the FP mode $f_{m=0.5}$ indicated by vertical lines. …"*

(on page 16, line 353)

*"**Fig. 4**. **Measurement precision analysis of LIB anodes. a** Measured reflection amplitude spectra for three nominal thicknesses (Case A, 50 μm; Case B, 100 μm; Case C, 150 μm), acquired with a period of 0.2 s over 200 s, with the FP mode $f_{m=0.5}$ indicated by vertical lines. …"*

(3) The reviewer's understanding is correct. **Figure 3b** shows thickness deviations retrieved from 1,000 repeated measurements acquired with a period of 0.2 s over 200 s, demonstrating the long-term measurement stability. The corresponding measurement precision is evaluated in **Fig. 3c** using Allan deviation analysis. The Allan deviation σ(τ) quantifies the fractional frequency (or thickness) fluctuations as a function of averaging time τ, defined as:

$$\sigma(\tau) = \sqrt{\frac{1}{2(M-1)}\sum_{i=1}^{M-1}(y_{i+1}-y_i)^2}$$

where y_i represents the i-th averaged measurement over averaging time τ, and M is the number of averaged samples. For white noise-dominated systems, the Allan deviation decreases as $\sigma(\tau) \propto \tau^{-1/2}$, indicating that precision improves with longer averaging times—a behavior clearly observed in our results.

Comment 6. Fig. 6 (a) and (b): The camera image appears in my printout as a solid grey box with no features whatsoever. Either choose a larger depiction or use different contrast, if this image is important for the story. Figure parts (d) and (e) are completely missing in the caption.

Response 6: We appreciate the reviewer's careful observation.

Following the reviewer's comment, we have removed the optical camera image inset from **Fig. 6a**, as the K-shaped step-height sample geometry is already described in the main text and clearly visualized in the 3D thickness map (**Fig. 6b**). We have also revised the **Fig. 6** caption to include descriptions for parts **d** and **e**:

(on page 21, line 447)

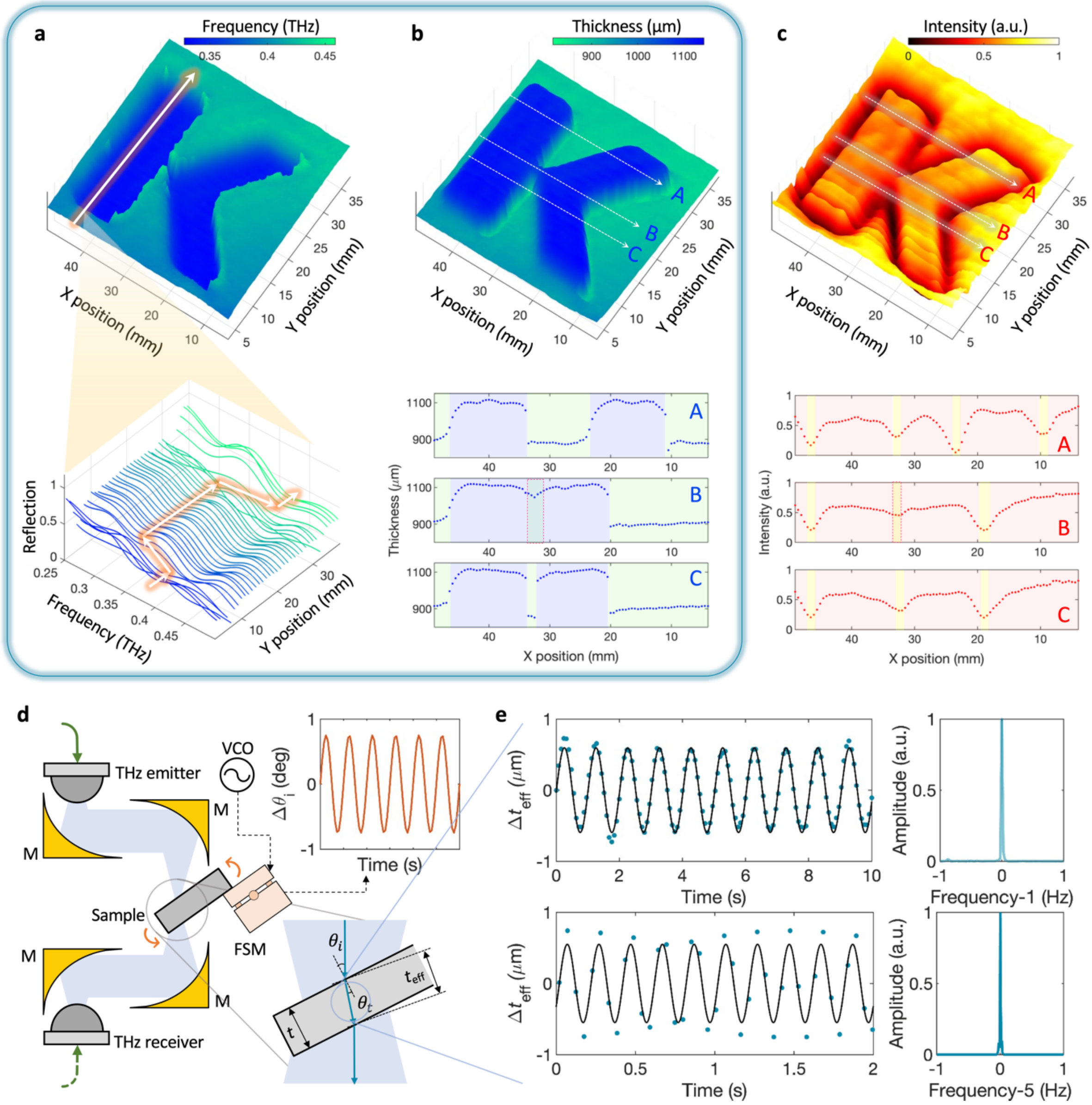

*Fig. 6. THz FP mode monitoring for thickness profiling and dynamic measurements. … **d** Schematic of the dynamic thickness measurement setup, with the right-top inset illustrating angle modulation of the fast steering mirror for effective optical path length modulation. **e** Measured effective thickness modulations fitted with a sinusoidal curve, along with its corresponding Fourier transform. M: off-axis parabolic mirror, FSM: fast-steering mirror, VCO: voltage-controlled oscillator.*

Comment 7. As already mentioned above, the part on page 22 on "miaslignement-tolerant, dynamic thickness measurements" is misleading as it is measure in transmission geometry and thus cannot be properly related to the reflection geometry shown in the other figures.

Response 7: We appreciate the reviewer's comment.

As detailed in our **Response 2** to **Reviewer #2**, the primary contribution of this work is the frequency comb referencing technique, which can be combined with various optical configurations with **Equations (1)** and **(2)** remaining universally applicable. The transmission geometry in **Fig. 6d–e** was

chosen as the simplest configuration to demonstrate the fast and precise dynamic measurement capability.

Following the reviewer's suggestion, we have revised the manuscript to clarify this purpose.

(on page 23, line 496)

> *"... This robustness ensures reliable thickness measurements despite significant angular deviations, making the technique suitable for integration into high-speed manufacturing environments where perfect alignment cannot always be maintained. Our frequency-comb-referenced THz source can be combined with various optical configurations, with* ***Equations (1)*** *and* ***(2)*** *remaining universally applicable. To demonstrate the fast and precise dynamic measurement capability, we employed a transmission geometry with a tilted quartz wafer as the simplest experimental configuration. We implemented a setup using a fast-steering mirror (FSM; OIM102, Optics in Motion) to periodically modulate the incidence angle θi, as depicted in* ***Fig. 6d****. ..."*

[Author's responses to Reviewer #3]

The authors report on an optical frequency-comb stabilized FDS THz spectrometer for inspection of lithium-ion battery electrodes. Through the frequency comb, which is stabilized to a Rb-clock, they achieve a high frequency-precision allowing for precise determination of Fabry-Perot-resonances of the electrode coatings, from which they extract the thicknesses and dielectric properties of the layers.
The topic of the manuscript is timely as battery production is increasingly important for various industries, especially electric cars. Here, THz non-destructive testing can provide significant advantages over other measurement techniques, such as X-ray or optical inspection.
The concept of measuring layer-thickness and material properties with a comb-stabilized THz FDS system as presented here is promising, however, the novelty of the work is limited, as various THz FDS measurement schemes for measurement of layer thickness and material-properties have been investigated and comb-stabilized spectroscopic THz-FDS measurements have also been demonstrated recently in the literature [1].
Furthermore, some of the claims made in the paper are questionable and the comparison to the literature could be stronger and more quantitative.
For the paper to be published in nature communications, the advantages of the system presented here should be made clearer and supported by quantitative comparisons. The following remarks should be addressed:

[1] Krause, B., Müller, S., Puppe, T. et al. Terahertz photonic heterodyne spectral analysis with (sub-) kHz resolution and 6.5 THz frequency coverage. Nat Commun 16, 10594 (2025).

Comment 1. The authors claim that TDS systems cannot reach below micrometre precision. However, in ref. 27 sub-micrometre precision has been shown for a TDS system. Ref. 17, which is cited to support this claim is rather old and not representative of the state of the art.

Response 1: We agree with the reviewer.

The reviewer is correct that recent TDS systems have demonstrated sub-micrometre precision, and we acknowledge that our original statement was overly broad.

Following the reviewer's comment, we have revised the 'Introduction' part of our manuscript to properly recognize the state-of-the-art performance of TDS systems:

(on page 5, line 83)

> *"... In TDS systems[17], femtosecond lasers have been widely used to generate broadband THz pulses, with recent advances achieving ~30–40 nm precision[18,19]. ..."*

Comment 2. Then, the authors argue that FDS systems also rely on time-delay measurements implying similar limitations as claimed for TDS systems. However, in ref. 18, the measurements are taken in the frequency domain and are only transformed into the time domain and evaluated there, which should not have a strong impact or follow the same limitations as the TDS systems, which rely on mechanical time delays. Furthermore, the precision reported in 18 is very similar to that reported by the authors in this work.

Response 2: We appreciate the reviewer's comment.

We agree that swept-laser-based FDS systems operate fundamentally differently from TDS systems that rely on mechanical time delays, and that comparable precision levels have been achieved. Rather than claiming superior precision, we have refocused our argument on the practical advantage of frequency-comb-referenced systems: the inherent reproducibility and elimination of periodic recalibration requirements, which are particularly relevant for industrial deployment.

Following the reviewer's comment, we have revised the manuscript to accurately acknowledge the performance of FDS systems:

(on page 5, line 85)

> *"... Swept-laser-based FDS systems have also achieved sub-micrometre precision[20], and sparse-frequency FDS methods have demonstrated remarkably fast acquisition times down to sub-milliseconds or ~60 nm precision with averaging[21]. ..."*

Comment 3. Furthermore, the authors claim, that other FDS systems require higher complexity due to calibration and look-up tables and are limited in measurement reliability. However, the use of a Rb-clock stabilized comb adds a significant amount of cost and complexity to the proposed system compared to the ones presented in refs. 18 and 27, which show a similar precision (down to below 50nm) and a strong stability over a long period of time. Additionally, there are works, achieving similar precision using only a few select frequencies, thus enabling very efficient systems with low complexity [2].

[2] Schwensonee, L., Walter, F., Jäckel, A. et al. Sparse Terahertz Frequency-Domain Sensing with Kilohertz Measurement Rate. J Infrared Milli Terahz Waves 46, 52 (2025).

Response 3: We appreciate the reviewer's comment.

We acknowledge that Rb-clock stabilized frequency combs do add system complexity compared to conventional swept-laser systems.

Regarding cost considerations, it is worth noting that the most widely used THz-TDS systems require at least one OFC as a femtosecond laser source, and dual-comb configurations are often employed for enhanced measurement accuracy. In contrast, swept-laser FDS systems utilize two CW lasers, offering a cost advantage. Our comb-referenced FDS system is constructed with one OFC and two CW lasers, placing its cost between conventional FDS and TDS systems. The relative cost hierarchy can be summarized as: FDS < TDS ≈ comb-FDS < dual-comb TDS. Therefore, while a frequency comb adds complexity, the overall system cost remains comparable to or lower than conventional TDS approaches. The added complexity of a frequency comb is justified not by achieving higher precision, but by providing SI-traceable, calibration-free operation that ensures long-term measurement consistency without periodic maintenance—a critical requirement for continuous industrial monitoring.

Following the reviewer's comment, we have revised the 'Introduction' to recognize sparse-frequency FDS methods and to clarify the specific advantage of our approach:

(on page 5, line 85)

> *"... Swept-laser-based FDS systems have also achieved sub-micrometre precision[20], and sparse-frequency FDS methods have demonstrated remarkably fast acquisition times down to*

sub-milliseconds or ~60 nm precision with averaging[21]. However, for battery electrodes, reported precision remains at the hundreds of nanometres level[22], and reliable industrial deployment demands SI-traceable frequency accuracy with long-term reproducibility. Stable and accurate THz sources offer a promising pathway to address these requirements, providing absolute frequency accuracy that enables consistent measurement reliability. ..."

Comment 4. Therefore, it seems questionable if the results achieved in this work justify the use of this complex comb stabilization, which would make such a system rather big and expensive especially considering the use-case in industrial battery manufacturing. The authors should try to convince the reader of this. For example, are there any quantitative requirements regarding the precision in electrode measurements to maintain a certain yield or safety, etc, that only this system can provide? Or are there any direct relations between precision and yield that could justify the use of this system?

Response 4: We appreciate the reviewer's comment.

We acknowledge that the frequency-comb-stabilized system is more complex than conventional swept-laser-based approaches. However, the frequency comb provides two critical advantages: (i) SI-traceable absolute frequency accuracy, enabling direct and consistent thickness determination, and (ii) long-term reproducibility [Ref. 24] without periodic recalibration.

We envision this system as a stand-alone, reference-grade metrology tool for initial process development, where establishing robust correlations between process parameters (slurry composition, coating speed, drying temperature) and electrode thickness requires highly precise and reproducible measurements. Conventional inspection methods, such as confocal microscopy-based destructive testing, suffer from measurement uncertainties of several micrometres, making it difficult to isolate the effect of individual process variables. In this context, the added complexity is justified by the superior measurement reliability.

Regarding the precision requirements and yield relationship, sub-micrometre precision is generally required for electrodes with thicknesses of several tens of micrometres, though standardized metrology methods have not yet been established. The general principle is well-established: tighter thickness control reduces defects such as non-uniform lithiation and capacity variations that compromise cell performance and safety. As electrode designs trend toward thinner layers for higher energy density and solid-state architectures, the demand for sub-micrometre precision will continue to increase. Our work demonstrates that such precision levels are achievable through frequency comb referencing, providing a pathway for future metrology standards in battery manufacturing.

Following the reviewer's comment, we have revised the manuscript as follows:

(on page 17, line 381)

"... Such precision, combined with the long-term reproducibility of comb-referenced measurements, enables reliable characterization of process-thickness correlations essential for electrode manufacturing optimization."

Comment 5. In general, a quantitative comparison to reported values in the literature (and possibly industry requirements) should be presented to support the claims made in this paper and strengthen the case for this approach.

Response 5: We appreciate the reviewer's constructive suggestion.

We agree that quantitative comparison is essential to support our claims and properly position our work within the existing literature (**Table R4**).

Table R4. Summary of thickness precision achieved by various terahertz metrology techniques. Precision values are categorized into single-shot and averaged measurements. References correspond to the data points shown in **Fig. S5**. The hyphen (-) indicates that averaged precision data is not available in the literature.

Reference	Method	Sample type	Sample	Precision (single)	Precision (averaged)	Acq. time
(i) This work	Comb-FDS	Battery	Cathode	465.5 nm	25.2 nm at 25.6 s	0.2 s
			Anode	70.1 nm	7.8 nm at 25.6 s	0.2 s
		Semiconductor	Silicon	106.3 nm	8.8 nm at 51.6 s	0.2 s
(ii) Mohammadzadeh, EuRAD (2023) [Ref. 22]	Swept-FDS	Battery	Cathode	~180 nm	-	0.005 s
			Anode	~270 nm	-	0.005 s
(iii) Liebermeister, Nat. Comm (2021) [Ref. 20]	Swept-FDS	Dielectric	PET	~300 nm	-	0.2 s
(iv) Schwenson, JIMTW (2025) [Ref. 21]	Sparse-FDS	Dielectric	Ceramic	~2000 nm	~60 nm at 0.8 s	0.0008 s
(v) Romero, Nat. Comm (2021) [Ref. 52]	FDS	Semiconductor	Silicon	289 nm	-	24 s
(vi) Molter, APR (2021) [Ref. 18]	TDS	Dielectric	Coating	40 nm	-	0.025 s
(vii) Liebermeister, JIMTW (2021) [Ref. 19]	TDS	Dielectric	PET	~30 nm	-	0.127 s

Regarding industry requirements, standardized metrology methods for electrode thickness have not yet been established. However, sub-micrometre precision is generally required for electrodes with thicknesses of several tens of micrometres. As electrode designs trend toward thinner layers for higher energy density and solid-state architectures, the demand for sub-micrometre precision will continue to increase. Our work demonstrates that such precision levels are achievable through frequency comb referencing.

Following the reviewer's comments, we have added a new subsection "Precision benchmarking with terahertz metrology" in the **Methods** section, along with **Supplementary Fig. 5**.

(on page 28, line 657)

*"**Precision benchmarking with terahertz metrology**. To benchmark our comb-referenced THz FP interferometry against existing techniques, we compiled thickness precision data from state-of-the-art THz metrology systems reported in the literature (**Supplementary Fig. 5**). Precision values were categorized into single-shot measurements and averaged measurements where available. For battery electrodes (**Supplementary Fig. 5a**), our system achieves 70.1-nm precision at 0.2-s acquisition for anodes, improving to 7.8-nm with 25.6-s integration—representing approximately one order of magnitude improvement in single-shot and two orders of magnitude with averaging compared to swept-laser-based FDS approaches[22]. For low-loss dielectrics and semiconductors (**Supplementary Fig. 5b**), TDS systems have demonstrated impressive single-shot precision down to several tens of nanometres[18], while swept-laser-based FDS[20] and sparse-frequency FDS[21] systems achieve comparable precision at rapid acquisition times down to sub-milliseconds. Our comb-FDS system matches this single-shot performance while surpassing these results through extended averaging, reaching sub-10 nm precision—a regime previously unexplored in THz metrology. A key advantage of our comb-referenced approach is its SI-traceable frequency accuracy, which suppresses long-term drift and enables continued precision improvement following*

the theoretical white-noise limit even with extended integration. This inherent frequency accuracy eliminates the need for periodic recalibration or look-up table maintenance, providing consistent and reproducible measurements over extended operation periods—a critical requirement for industrial deployment."

(on page 37, line 839)

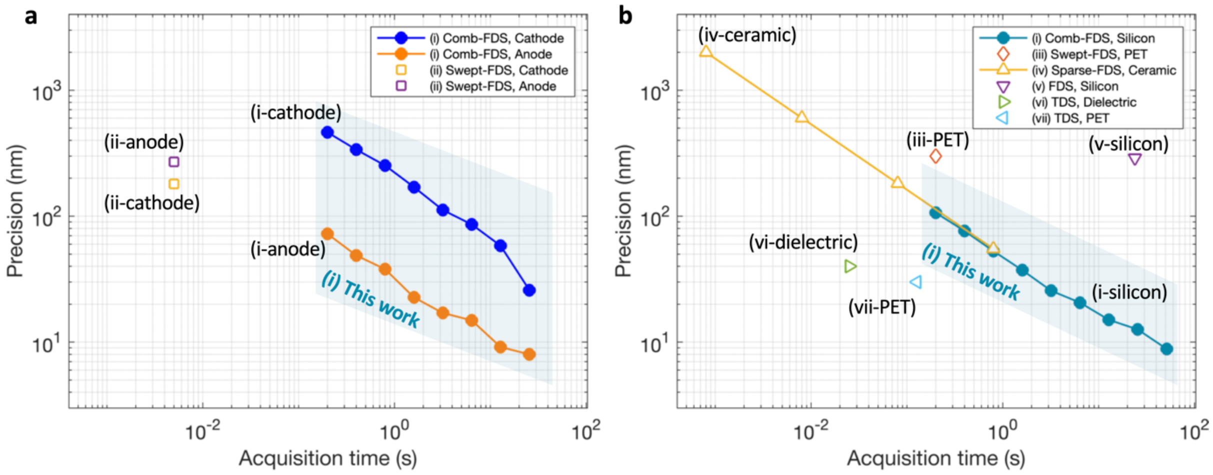

Fig. S5. Performance comparison with state-of-the-art terahertz thickness metrology in terms of thickness precision vs. acquisition time. a *Battery electrode measurements; (i) this work, comb-referenced FDS (cathode and anode), (ii) swept-laser-based FDS (cathode and anode)[22]. This work (shaded region) achieves 70.1-nm precision at 0.2-s for anodes, improving to sub-10 nm at 25.6-s integration—demonstrating one-to-two orders of magnitude improvement over swept-laser-based FDS for battery electrode metrology.* ***b*** *Low-loss dielectric and semiconductor measurements; (i) this work, comb-referenced FDS (silicon), (iii) swept-laser-based FDS (PET)[20], (iv) sparse-frequency FDS (ceramic)[21], (v) FDS (silicon)[52], (vi) TDS (dielectric coating)[18], (vii) TDS (PET)[19]. Our system achieves comparable precision of several tens of nanometres in single-shot measurements, while reaching sub-10 nm precision with averaging—a regime previously unexplored in terahertz metrology.*

Comment 6. The authors claim a two-orders-of-magnitude improvement over conventional methods; however, no quantitative numbers or references are provided to support this claim.

Response 6: We appreciate the reviewer's comment.

We acknowledge that our original claim lacked specific quantitative comparisons. Based on our literature review (**Table R4** and **Fig. S5**), the state-of-the-art precision for battery electrode thickness measurement using THz methods is ~180–270 nm (swept-laser-based FDS [Ref. 22]). Our comb-referenced system achieves 70 nm (anode) and 466 nm (cathode) precision at 0.2-s single-shot acquisition—comparable to or one order of magnitude better than conventional methods. With 25.6-s averaging, precision improves to 7.8 nm (anode) and 25.2 nm (cathode), representing one-to-two orders of magnitude improvement.

Following the reviewer's comment, we have revised the manuscript to clarify the improvement with specific context:

(on page 2, line 25)

"... confirming one-to-two orders of magnitude improvement over the counterparts relying on temporal analysis for battery electrode metrology."

(on page 5, line 105)

"... demonstrating an order-of-magnitude enhancement in single-shot measurements and up to two orders of magnitude with averaging compared to conventional temporal-analysis-based methods for battery electrode metrology."

Comment 7. Does the AOM introduce amplitude modulation as well? From experience, this could be imprinted on the PCA output as noise...

Response 7: We appreciate the reviewer's insightful question.

In our system, the AOMs function purely as frequency shifters for coherent heterodyne detection, producing a net intermediate frequency of f_h = 80 kHz. The VCOs driving the AOMs are referenced to the same Rb atomic clock that stabilizes the frequency comb, ensuring negligible phase noise contribution.

This noise-free AOM operation has been validated in our previous work [Ref. 27], where comprehensive noise analysis identified only TIA intrinsic noise, PCA shot noise, and PCA thermal noise as dominant sources—with no detectable AOM-related contribution.

Following the reviewer's comment, we have clarified the AOM configuration in the Methods section:

(on page 25, line 571)

"To enable coherent detection of THz amplitude and phase spectra, a heterodyne frequency f_h = 80 kHz is introduced using a frequency shifter (FS) comprising a pair of acousto-optic modulators (AOMs), with their RF drive frequencies referenced to the Rb atomic clock, which shift the fixed laser frequency v_1 by f_{a1} = 40.04 MHz and f_{a2} = 39.96 MHz, respectively, such that $f_h = f_{a1} - f_{a2}$."

Comment 8. The anode shows higher precision despite the stronger absorption, which should reduce SNR and thus precision. The authors explain this by the thicker sample, which shifts the resonance towards lower frequencies where higher SNR is available, and by the reduced thickness deviations in the high-index materials. Regarding the former, this is not very intuitive and a formula/simulation showing the SNR at the FP resonance as a function of thickness would help. Regarding the former, should the thickness deviations across samples not be independent of the precision of the measurement? And is this reduced deviation a direct consequence of the high index or just a coincidence? I think this finding should be discussed in more detail.

Response 8: We appreciate the reviewer's comment.

The higher precision observed in anodes, despite their stronger absorption, can be explained by the relationship between FP mode frequency position and measurement precision. Anodes have a higher refractive index (n = 6.0) compared to cathodes (n = 2.9), resulting in a larger optical path difference (OPD = $2nt$). This shifts the FP modes to lower frequencies, where our THz source provides higher SNR.

To clarify this relationship quantitatively, we performed a systematic analysis using a silicon wafer with multiple observable FP modes (**Supplementary Fig. 3**). The analysis reveals two key findings:

(1) Frequency precision degrades at higher frequencies due to reduced SNR of the THz source (left panel of **Supplementary Fig. 3c**). This confirms that FP modes at lower frequencies yield better frequency precision.

(2) Thickness precision exhibits a different trend compared to frequency precision. By differentiating **Eq. (2)**, the thickness precision δt is expressed as: $\delta t = (c / 2nf_{FSR}^2)\times(1/m)\times\delta f_m$. This relation reveals a division effect: while thickness precision is proportional to frequency precision δf_m, it is inversely proportional to the mode number m. Consequently, even though frequency precision degrades at higher-order modes, the factor $(1/m)$ partially compensates for this degradation (**Supplementary Fig. 3d**). For anodes, the higher refractive index ($n = 6.0$) places the $f_{m=0.5}$ mode at a lower frequency than for cathodes ($n = 2.9$), where the THz source provides higher SNR, directly resulting in superior thickness precision.

Following the reviewer's comment, we have added a new **Methods** section with detailed analysis:

(On page 26, line 595)

> *"**Precision analysis of each FP mode.** To evaluate the precision characteristics of each FP mode, we analysed the frequency and thickness precision as a function of mode number (i.e., frequency position) based on measurements of a silicon wafer. **Supplementary Fig. 3a** shows the transmission amplitude spectrum of a 525-µm thick silicon wafer, where eleven destructive FP modes ($f_{m=1.5}$ to $f_{m=11.5}$) are observed across the 0.1–1.0 THz range. As frequency increases, the SNR decreases, leading to degraded frequency precision (**Supplementary Fig. 3b**). …"*

(On page 35, line 818)

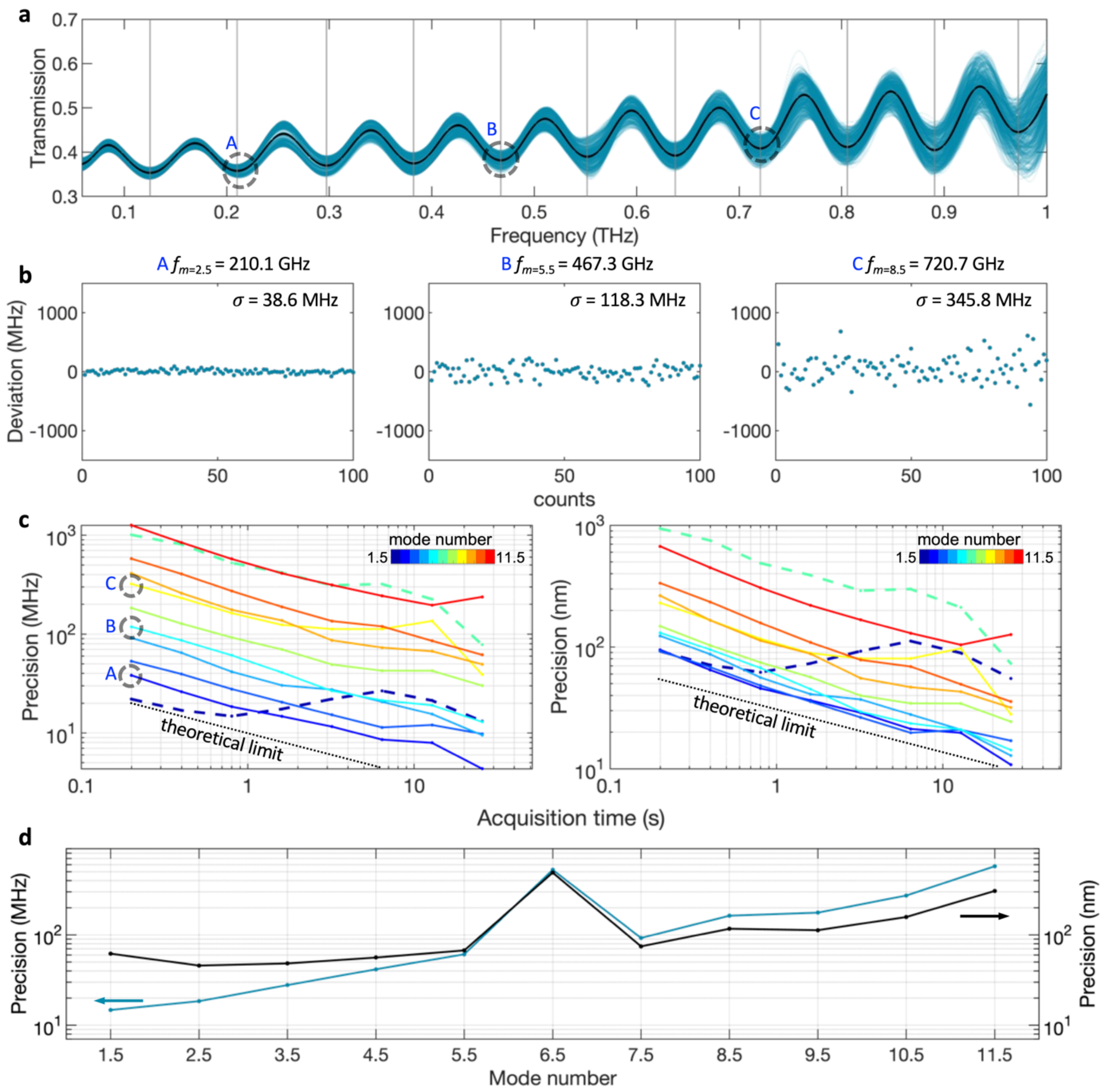

Fig. S3. Precision analysis of frequency and thickness measurements as a function of FP mode number. ***a*** *Transmission amplitude spectra of a 525-μm thick silicon wafer measured over 1,000 repeated acquisitions across the 0.1–1.0 THz range. The black solid line represents the averaged spectrum, and grey vertical lines indicate the centre positions of destructive FP modes.* ***b*** *Deviation of FP mode centre frequencies for three different FP modes (Case A: $f_{m=2.5}$ = 210.1 GHz, Case B: $f_{m=5.5}$ = 467.3 GHz, Case C: $f_{m=8.5}$ = 720.7 GHz).* ***c*** *Allan deviation of FP mode frequency precision (left panel) and the retrieved thickness precision (right panel).* ***d*** *Frequency precision (blue, left y-axis) and thickness precision (black, right y-axis) as a function of mode number at 0.8-s acquisition time. The thickness precision shows a different behaviour compared to frequency precision due to the inverse dependence on mode number m.*

Comment 9. Regarding the SEM cross-sections inf Fig 3d, the authors state that the higher uncertainty in the SEM measurements stems from non-optimized sample preparation. Is that a common thing in industrial contexts for this type of measurement? Otherwise, the comparison of the two methods here would not be entirely fair, if better preparation is possible.

Response 9: We appreciate the reviewer's comment.

With optimized ion-milling conditions, it is possible to obtain clean cross-sections without significant sample damage. However, the optimal milling parameters (ion beam energy, milling time, incident angle) vary depending on electrode characteristics such as composition, porosity, and thickness. Consequently, achieving reliable cross-sectional SEM measurements requires iterative optimization of ion-milling conditions for each electrode type, which is time-consuming and resource-intensive. In industrial contexts, this optimization burden is a practical limitation that adds to the overall measurement uncertainty.

In contrast, our THz FP interferometry operates in a non-contact, non-destructive manner, eliminating the need for sample preparation and the associated optimization efforts. This advantage is particularly relevant for industrial applications where rapid and consistent measurements across diverse electrode types are required.

Following the reviewer's comment, we have clarified this point in the revised manuscript:

(on page 14, line 312)

"... This necessity highlights the practical limitations of destructive methods and underscores the value of our non-destructive THz interferometric approach, which enables nanometre-level precision without the need for invasive sample preparation—such as iterative optimization of ion-milling conditions for each electrode type—and is inherently immune to surface scattering."

Comment 10. The authors claim sub-micrometre precision on their samples while admitting several hundreds of microns of roughness on the samples. This sounds contradictive. Unless the THz spot size is much smaller than the roughness, I do not see a way to make a measurement more precise than the roughness as the measurements would always be an average over the THz spot on the sample (which is later in the manuscript determined to be on the order of millimetres). Please explain.

Response 10: We appreciate the reviewer's comment.

We would like to note that the surface roughness of our electrode samples is on the sub-micrometre to micrometre scale (R_a ~0.8 μm for cathodes, ~1.3 μm for anodes), not several hundreds of micrometres.

The sub-micrometre precision reported in our work refers to the repeatability of measurements at a single point—quantified by the Allan deviation of repeated thickness retrievals from the same location. This precision characterizes the measurement system's ability to reproduce consistent results, independent of the thickness uniformity across the sample.

As the reviewer correctly notes, the THz beam (defined by a ~5 mm diameter aperture) averages the thickness over the illuminated spot. The measured value thus represents a spatially averaged thickness within this region. Thickness variations across different positions can be characterized through thickness profiling, as demonstrated in **Fig. 6a–c**.

Regarding surface roughness, the long THz wavelengths (300–3000 μm in our measurement range) are significantly larger than the micrometre-scale surface roughness, making THz radiation inherently immune to surface scattering effects that would degrade optical or NIR measurements. This surface roughness immunity has been demonstrated in our recent work on silicon wafer thickness measurements [Ref. 36], confirming that sub-micrometre precision is achievable even on samples with micrometre-scale surface roughness.

Following the reviewer's comment, we have clarified the meaning of precision in the revised manuscript:

(on page 13, line 280)

*"**Figure 3b** shows the resulting deviations in retrieved thickness t_{THz}, demonstrating sub-micrometre-level precision—defined as single-point measurement repeatability—for all three cases."*

Comment 11. In figure 3 and 4, there are some strong deviations between the thicknesses determined through THz and SEM, sometimes on the order of 20% and with the error-bars of the two measurements not overlapping at all. How do the authors explain this and what would be the ground truth then? A more detailed explanation on how the thickness is extracted from the SEM images would also be helpful.

Response 11: We appreciate the reviewer's comment.

To provide a more reliable SEM thickness determination, we have improved the extraction methodology and updated the relevant figures and discussion in the revised manuscript.

In the original manuscript, t_{SEM} was determined by manually measuring the coating thickness at only 2–3 discrete points. To improve statistical reliability, we implemented an image processing approach: (1) binarization of cross-sectional SEM images to segment the coating layer, and (2) pixel-column-by-pixel-column thickness extraction across ~1,280 columns (**Figures R2** and **R3**). The mean t_{SEM} and spatial non-uniformity σ are obtained from these profiles.

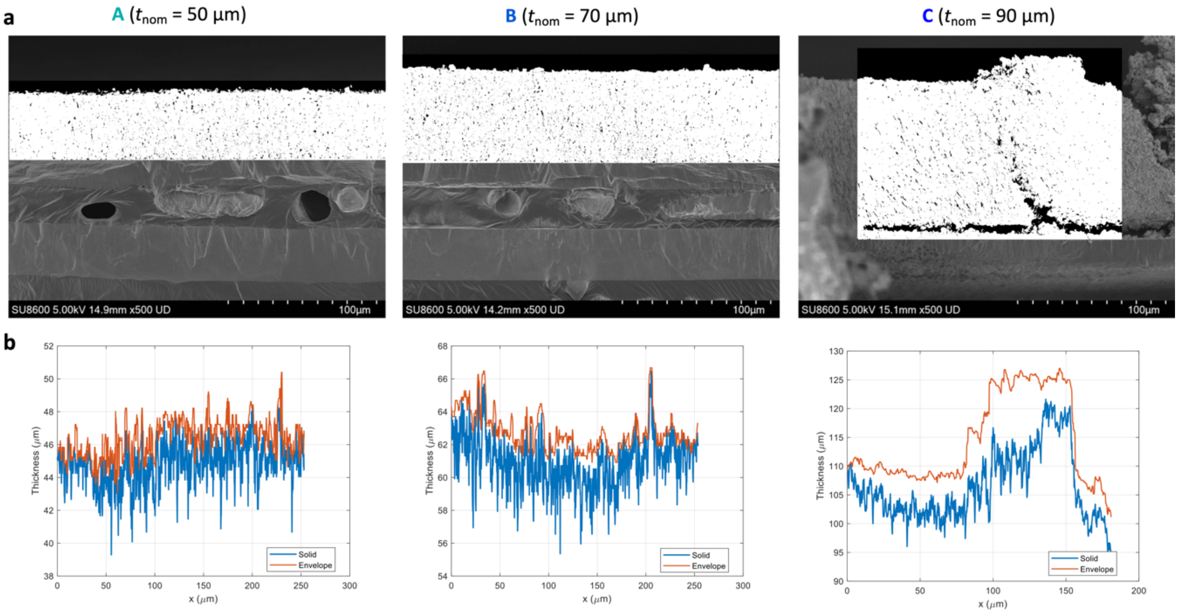

Fig. R2. Thickness extraction from cross-sectional SEM images of LIB cathodes. a Cross-sectional SEM images and their binarized representations used to segment the electrode coating layer (Case A, t_{nom} = 50 µm; Case B, t_{nom} = 70 µm; Case C, t_{nom} = 90 µm). **b** Thickness profiles determined from pixel-column analysis of the binarized images, with (orange) and without (blue) porosity inclusion. The mean thickness t_{SEM} is obtained by averaging the local thickness values with porosity inclusion (orange) across ~1,280 pixel columns, and the spatial non-uniformity σ is defined as the standard deviation of these values.

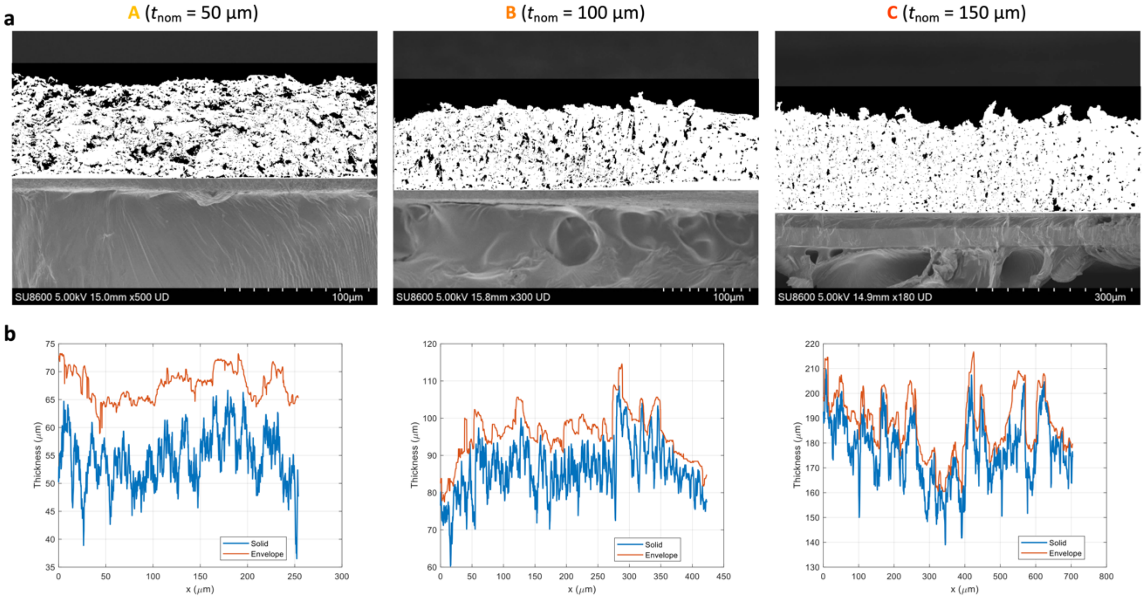

Fig. R3. Thickness extraction from cross-sectional SEM images of LIB anodes. a Cross-sectional SEM images and their binarized representations used to segment the electrode coating layer (Case A, t_{nom} = 50 μm; Case B, t_{nom} = 100 μm; Case C, t_{nom} = 150 μm). **b** Thickness profiles determined from pixel-column analysis of the binarized images, with (orange) and without (blue) porosity inclusion. The mean thickness t_{SEM} is obtained by averaging the local thickness values with porosity inclusion (orange), and the spatial non-uniformity σ is defined as the standard deviation of these values.

Accordingly, the SEM-related values in **Figures 3d, 3e, 4d, and 4e** have been updated.

(on page 13, line 275)

> *"**Fig. 3.** … Error bars indicate the measurement precision for t_{THz} and the spatial non-uniformity for t_{SEM}, respectively."*

(on page 15, line 320)

> *"… The error bars indicate the measurement precisionfor t_{THz} and the spatial non-uniformity for t_{SEM}, respectively. The t_{SEM} values were determined by averaging the thickness across the entire cross-sectional SEM images after binarization processing. …"*

(on page 16, line 354)

> *"**Fig. 4.** … Error bars indicate the measurement precision for t_{THz} and the spatial non-uniformity for t_{SEM}, respectively."*

(on page 17, line 367)

> *"… However, SEM imaging still exhibits larger error bars due to the inherent spatial non-uniformity of the electrode coating, as well as artifacts introduced during the ion milling process. …"*

As shown in **Table R5**, the deviations between t_{THz} and t_{SEM} range from 1.3 to 20.6 μm (2.8–36.0%). These are primarily attributed to three factors. First, the two methods do not measure the same location—the THz beam spot (~5mm diameter) provides a spatially averaged thickness, whereas SEM captures only a ~300 μm-wide cross-section at a different position. Given the large spatial non-uniformity of LIB electrodes (e.g., σ = 7.75 μm for cathode C, σ = 12.53 μm for anode C), discrepancies

between different measurement locations are inevitable. Second, the ion milling process for SEM sample preparation can introduce artifacts such as curtain effects, delamination, and deviations from perpendicular cross-sectioning, systematically biasing t_{SEM}, as described in **Fig. S4**. Third, THz interferometry measures optical path length and converts it using the estimated refractive index, while SEM directly images the physical cross-section, so local variations in refractive index due to compositional or porosity gradients can also contribute to the deviation.

Table R5. Comparison of thickness values obtained by THz interferometry (t_{THz}) and cross-sectional SEM imaging (t_{SEM}) for LIB cathode and anode samples. The deviation of t_{THz} represents the single-measurement precision (1σ) at 0.2-s acquisition time, while the deviation of t_{SEM} represents the spatial non-uniformity (1σ) obtained from binarized cross-sectional SEM images (**Figures R2** and **R3**). $\Delta t = |t_{THz} - t_{SEM}|$, and the relative deviation is calculated as $\Delta t/((t_{THz} + t_{SEM})/2) \times 100\%$.

Sample	t_{nom} (μm)	t_{THz} (μm)	t_{SEM} (μm)	Δt (μm)	$\Delta t/((t_{THz}+t_{SEM})/2)$ (%)
Cathode	A (50 μm)	45.1 ± 0.85	46.4 ± 1.13	1.3	2.8%
	B (70 μm)	50.9 ± 0.71	62.8 ± 1.18	11.9	20.9%
	C (90 μm)	105.2 ± 0.47	114.4 ± 7.75	9.2	8.4%
Anode	A (50 μm)	47.0 ± 0.17	67.6 ± 2.78	20.6	36.0%
	B (100 μm)	103.4 ± 0.07	95.9 ± 6.44	7.5	7.5%
	C (150 μm)	170.3 ± 0.17	187.9 ± 12.53	17.6	9.8%

Therefore, neither t_{THz} nor t_{SEM} serves as an absolute ground truth for these non-uniform electrode samples. The observed deviations reflect the inherent challenges of comparing two fundamentally different methods on spatially non-uniform samples, rather than systematic errors in either technique.

In the material characterization section

Comment 12. The authors mention that the position of the dominant spectral dip changes with k. In figure 5, however, the dip appears to stay at the same frequency and only change depth and width over k. Please explain.

Response 12: We appreciate the reviewer's comment.

The reviewer is correct that the frequency positions of individual FP dips ($f_{m=0.5}$, $f_{m=1.5}$, etc.) remain constant regardless of κ. What changes with κ is which dip becomes dominant (i.e., the deepest). In **Fig. 5a**, varying the refractive index n induces systematic redshifts in all FP mode frequencies, as the resonance condition directly depends on n via **Eq. (1)**. On the other hand, **Fig. 5b**, shows the dominant dip transitions from $f_{m=1.5}$ at $\kappa < 0.3$ to $f_{m=0.5}$ at $\kappa \geq 0.3$ as κ increases. The white dotted lines track this transition, highlighting which dip is dominant at each κ value—not a shift in the dip frequency itself.

We acknowledge that the original wording "alters the position of dominant spectral dips" was misleading. Following the reviewer's comment, we have revised the manuscript for clarity:

(on page 17, line 390)

> *"... As n increases, systematic redshifts in FP modes are observed (**Fig. 5a**). In contrast, increasing κ causes the dominant (deepest) dip to transition from $f_{m=1.5}$ at $\kappa < 0.3$ to $f_{m=0.5}$ at κ*

*≥ 0.3 (**Fig. 5b**). This transition, highlighted by white dotted lines in **Fig. 5b**, serves as a useful spectral feature for constraining the complex refractive index of arbitrary LIB cathodes through model-based fitting.”*

Comment 13. In figure 5d and S1d, which sample was used, A, B or C? Consider showing these plots for all three samples for the reader to get a better feeling of how these plots vary for different samples.

Response 13: We appreciate the reviewer's suggestion.

Following the reviewer's comment, we have prepared cross-correlation analysis results for all cathode and anode samples (**Fig. R4**). The complex refractive index was estimated by comparing measured spectra with a simulated spectra database, where n and κ were swept in 0.05 increments, and selecting the n+iκ values yielding the maximum cross-correlation coefficient (XCORR).

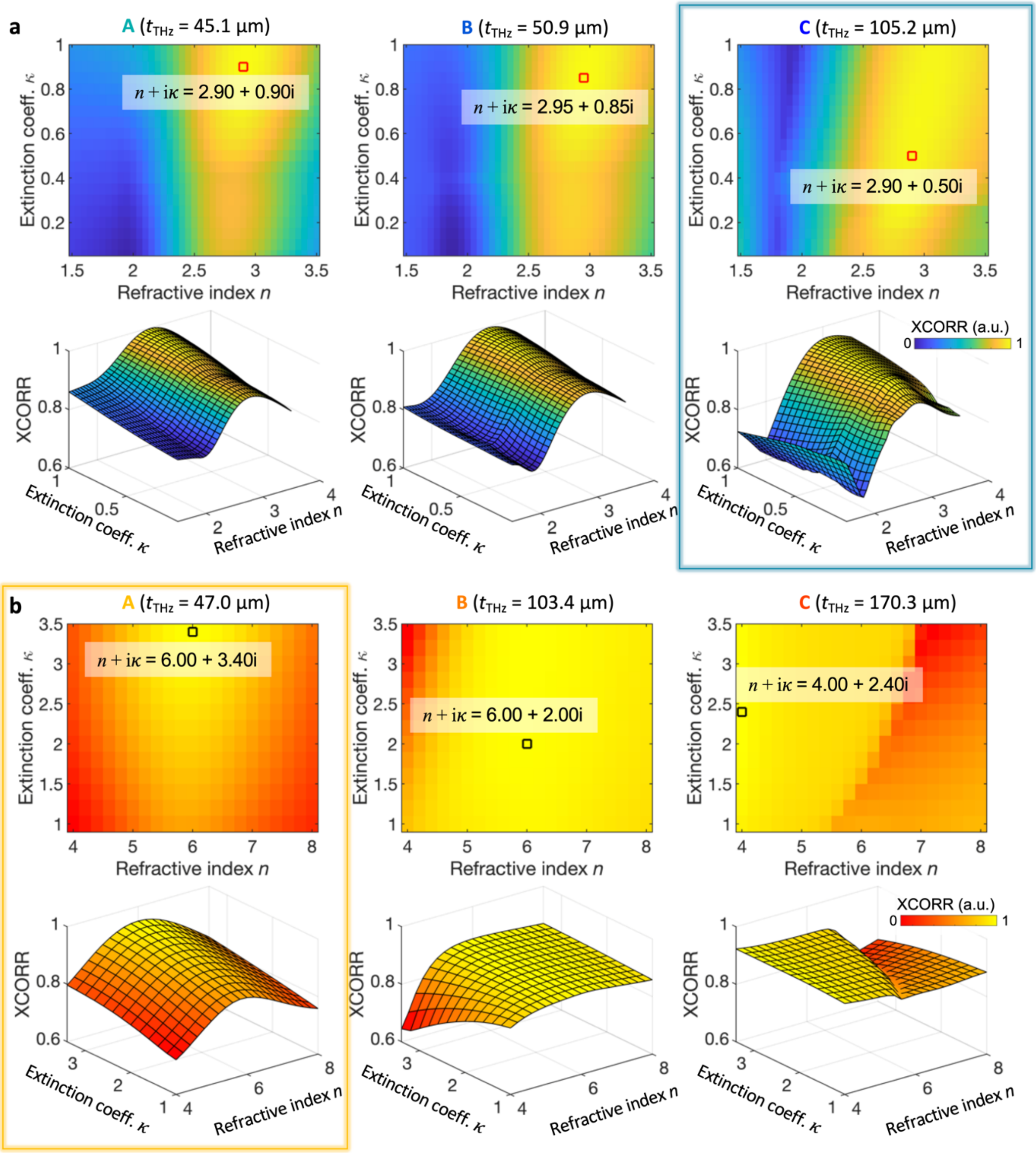

Fig. R4. Cross-correlation analysis for determining the complex refractive index of LIB electrodes. Each panel shows the cross-correlation coefficient (XCORR) between measured and simulated spectra as a function of refractive index *n* and extinction coefficient *κ*: 2D map (top) and 3D surface (bottom). The estimated n+i$κ$ values corresponding to the maximum XCORR are indicated by markers. **a** LIB cathodes for three nominal thicknesses (Case A, 50 μm; Case B, 70 μm; Case C, 90 μm) with measured t_{THz} = 45.1, 50.9, and 105.2 μm, respectively. **b** LIB anodes for three nominal thicknesses (Case A, 50 μm; Case B, 100 μm; Case C, 150 μm) with measured t_{THz} = 47.0, 103.4, and 170.3 μm, respectively.

For cathodes (**Fig. R4a**), the XCORR surfaces exhibit consistent trends across different sample thicknesses. The estimated refractive index *n* ranges from 2.90 to 2.95 across Cases A–C, showing good consistency. However, the extinction coefficient *κ* varies more broadly between 0.5 and 0.9, exhibiting larger deviation. This variation can be attributed to several factors: (1) the limited spectral bandwidth constraining the uniqueness of the estimation, (2) the relatively coarse step size (0.05) in the *n*–*κ* parameter sweep introducing discretization ambiguity, and (3) the assumption of frequency-independent optical constants, whereas actual electrode materials may exhibit frequency-dependent behaviour. The final complex refractive index for cathodes was determined as n+i$κ$ = 2.9+0.5i, derived from Case C which exhibits the highest XCORR among the three samples. This higher XCORR is attributed to the presence of two FP modes ($f_{m=0.5}$ and $f_{m=1.5}$) within the measurement bandwidth (<1 THz), whereas Cases A and B show only a single mode ($f_{m=0.5}$). **Figure 5c** demonstrates that simulations using this estimated refractive index show reasonable agreement with the measured spectra for all three cases.

For anodes (**Fig. R4b**), the estimated complex refractive index exhibits larger variation due to the higher absorption limiting the effective spectral bandwidth. Case A, with the broadest effective bandwidth (<0.4 THz), yields the most reliable estimation, while Cases B and C have progressively narrower bandwidths (<0.2 THz and <0.15 THz, respectively), resulting in less distinct XCORR peaks. Based on this analysis, we have revised the anode complex refractive index in the manuscript from the original value of n+i$κ$ = 6.0+2.0i (previously derived from Case B) to n+i$κ$ = 6.0+3.4i (derived from Case A). All relevant values in the manuscript and **Figure S1c** have been updated accordingly.

These results provide practical guidelines for material characterization of battery electrodes. For lower-absorption cathodes, thicker samples are preferable as they generate multiple FP modes within the measurement bandwidth, providing stronger constraints for the cross-correlation fitting. Additionally, broadband measurements with extended acquisition time and improved SNR can further enhance the accuracy of refractive index estimation. For higher-absorption anodes, thinner coatings are advantageous as they reduce absorption losses and enable observation of FP modes over a broader spectral region, thereby improving the reliability of material characterization.

Following the reviewer's comment, we have revised the manuscript to clarify the sample selection for refractive index estimation and to provide practical guidelines:

(on page 18, line 404)

> *"Samples with multiple FP modes (i.e., case C of* ***Fig. 5c****) improved the accuracy of the XCORR calculation by providing stronger constraints for the cross-correlation fitting."*

(on page 19, line 408)

> *"For the anode, the estimated complex refractive index was n+i$κ$ = 6.0+3.4i, derived from Case A which provides the broadest effective spectral bandwidth among the anode samples, …"*

(on page 19, line 410)

"For reliable material characterization, lower-absorption materials such as cathodes benefit from thicker samples that generate multiple FP modes, while higher-absorption materials such as anodes benefit from thinner coatings that enable broader spectral observation."

Comment 14. In figure 5c and s1c, consider plotting measurements and simulation in the same plot so the reader can evaluate the fit between measurement and simulation better.

Comment 15. Additionally, in fig 5c and s1c, the authors claim strong agreement between measurement and simulation. However, there are strong differences in the depth and width of the dips, and the FP frequencies also show deviations which could be on the order of 10% or more (difficult to tell exactly as the curves are in separate plots). This is particularly pronounced for fig. s1c. The origin of these deviations should be discussed in more detail.

Response 14&15: We appreciate the reviewer's suggestions.

Following the reviewer's comment, we have prepared an overlay plot (**Fig. R5**) for direct comparison. For both cathodes (**Fig. R5a**) and anodes (**Fig. R5b**), the positions of the first FP mode $f_{m=0.5}$ show reasonable agreement between measurement and simulation. However, deviations in dip depth, width, and higher-order mode positions are evident. These deviations originate from: (1) the inherently limited resolution of cross-correlation-based estimation, where XCORR values remain broadly high across a wide range of $n+i\kappa$ values (**Fig. 5d**), (2) the assumption of frequency-independent optical constants, whereas actual electrode materials may exhibit frequency-dependent behaviour, and (3) stronger absorption in anodes, which amplifies sensitivity to inaccuracies in the assumed optical constants.

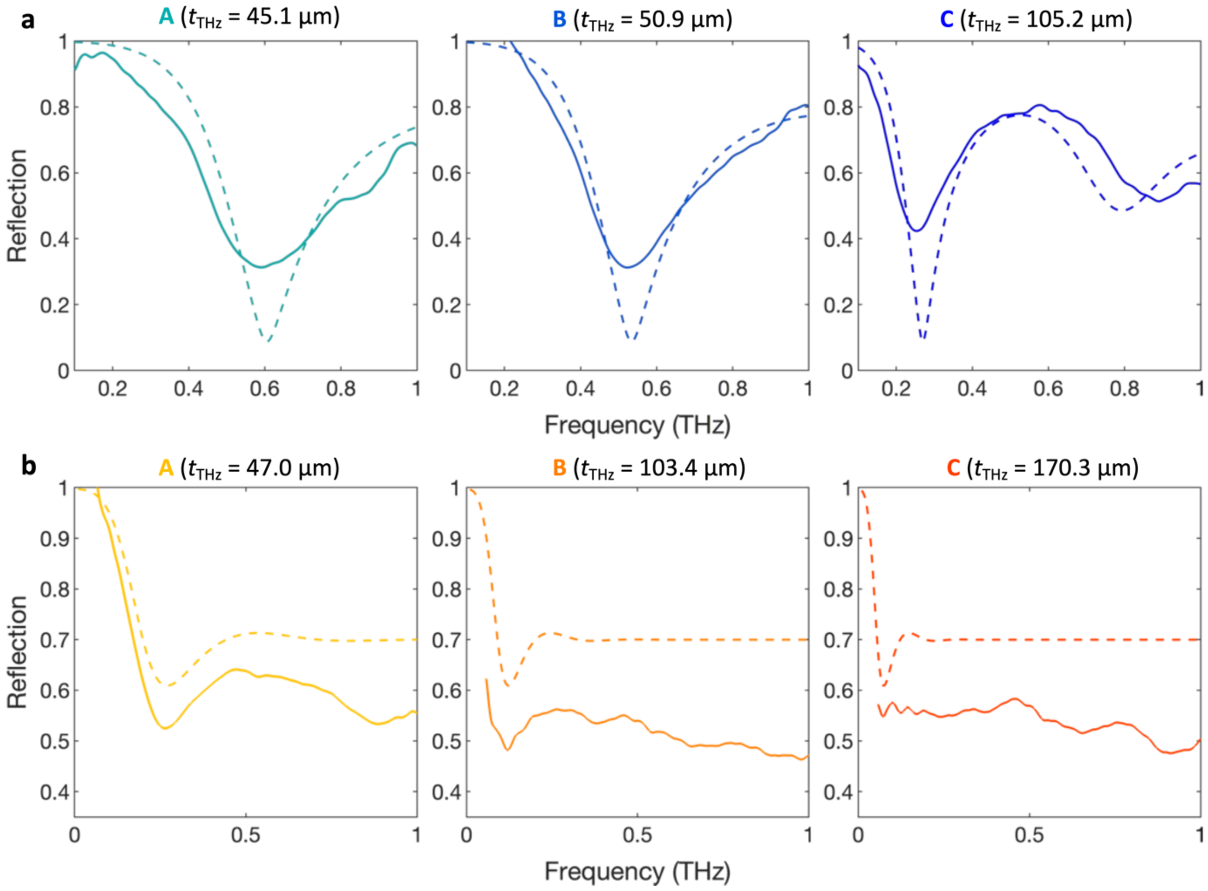

Fig. R5. Overlay of measured (solid lines) and simulated (dashed lines) reflection amplitude spectra. Simulated spectra were calculated using the measured thickness t_{THz} and the complex refractive index estimated

via cross-correlation for each electrode type. **a** LIB cathodes: Case A (t_{THz} = 45.1 μm), Case B (t_{THz} = 50.9 μm), and Case C (t_{THz} = 105.2 μm), simulated with n+iκ = 2.9+0.5i. **b** LIB anodes: Case A (t_{THz} = 47.0 μm), Case B (t_{THz} = 103.4 μm), and Case C (t_{THz} = 170.3 μm), simulated with n+iκ = 6.0+3.4i.

Despite these limitations, the estimated complex refractive indices successfully reproduce the overall spectral shape and FP mode positions across multiple samples, confirming their validity for thickness determination.

Following the reviewer's comment, we have revised the manuscript as following:

(on page 18, line 403)

> *"**Figure 5c, d** present the comparison between measured and simulated reflection amplitude spectra and the corresponding cross-correlation coefficients (XCORR), respectively. Samples with multiple FP modes (i.e., case C of **Fig. 5c**) improved the accuracy of the XCORR calculation by providing stronger constraints for the cross-correlation fitting. Consequently, the complex refractive index for the cathode was estimated to be approximately 2.9+0.5i, and simulations based on this value reproduced the measured spectra with reasonable agreement, as seen in **Fig. 5c**."*

Comment 16. The authors mention the extinction coefficient of the PLA is approximately 0.13. Where does this value come from? Is it measured as like the refractive index and if not, why not?

Response 16: We appreciate the reviewer's comment.

Although the complex refractive index (n+iκ) was presented in the original manuscript, thickness determination via **Eq. (2)** primarily depends on the refractive index n. As shown in **Fig. R6**, we prepared three 3D-printed PLA slabs with varying thickness and fill density (f.d.): (i) 1 mm with 15 f.d., (ii) 1 mm with 100 f.d., and (iii) 3 mm with 100 f.d. The refractive index n = 1.57 was determined by measuring the FP mode frequencies of samples with known printed thickness. Due to the absorptive nature of PLA, FP modes are predominantly observed below 0.5 THz, as indicated by the grey-shaded region where high absorption makes FP modes less distinguishable. We also confirmed that the refractive index varies with fill density, consistent with the literature [Ref. 42].

The extinction coefficient $\kappa \approx 0.13$ was adopted from Ref. 42. However, once the appropriate FP mode is selected for thickness monitoring in the low-absorption spectral region, κ does not significantly affect the thickness determination. Therefore, we removed the extinction coefficient from the revised manuscript for clarity.

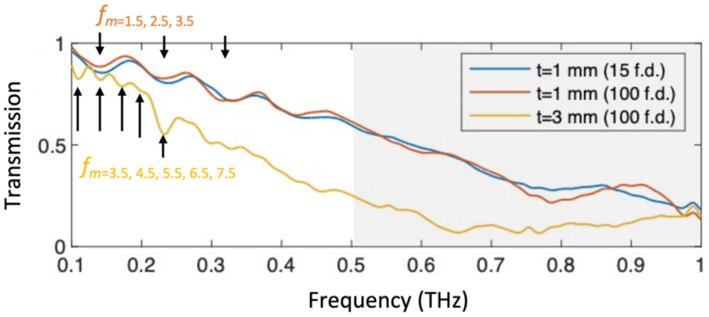

Fig. R6. Measured transmission spectra of 3D-printed PLA samples with varying thickness and fill density. Three configurations are shown: t = 1 mm with 15% f.d. (blue), t = 1 mm with 100% f.d. (orange), and t = 3 mm

with 100% f.d. (yellow). FP modes are indicated by arrows, with $f_{m=1.5,\ 2.5,\ 3.5}$ for the 1 mm samples and $f_{m=3.5,\ 4.5,\ 5.5,\ 6.5,\ 7.5}$ for the 3 mm sample. The grey-shaded region (>0.5 THz) indicates high absorption where FP modes become less distinguishable.

Following the reviewer's comment, we have revised the manuscript for better clarity:

(on page 20, line 444)

> *"... The sample was printed using a 3D printer (K13D, Creality), featuring a base thickness of 880 µm and a K-shaped protrusion reaching 1,100 µm. The refractive index n = 1.57 was used for converting FP mode frequencies into thickness values via* ***Eq. (2)****."*

Comment 17. In figure 6e the authors claim excellent agreement between theory and measurement. While this is valid for the 1 Hz case, the 5 Hz measurements show some strong deviations in the oscillation amplitude. This could be discussed in more detail.

Response 17: We appreciate the reviewer's comment.

As illustrated in **Fig. 6d**, the quartz wafer sample is mounted at the end of the fast-steering mirror (FSM) arm. At higher modulation frequencies such as 5 Hz, the mechanical configuration can induce additional vibrations beyond the intended angular modulation, resulting in larger oscillation amplitudes compared to the 1 Hz case. Despite this mechanical artifact, the measured modulation frequencies (confirmed by Fourier transform analysis) accurately match the commanded frequencies, demonstrating that our system successfully tracks dynamic thickness variations at rates up to 5 Hz.

Following the reviewer's comment, we have added a clarification in the revised manuscript:

(on page 23, line 509)

> *"****Figure 6e*** *shows the measured effective thickness variations Δt_{eff} at modulation frequencies of 1 Hz (upper panel) and 5 Hz (lower panel), exhibiting peak-to-peak amplitudes of ~2 µm, in excellent agreement with theoretical predictions. The slightly larger amplitude deviation at 5 Hz is attributed to additional mechanical vibrations induced by the FSM-mounted sample configuration at higher modulation frequencies. ..."*

Discussion:

Comment 18. The authors claim unprecedented precision for the 68µm anode. This is hard to verify for the reader as no reference values are given from other works to compare this against. (1) A table comparing the results of this work against the literature would be important to strengthen the case for this work. This table should include the measurement type, electrode type, material and thickness as well as the measurement precision. (2) Furthermore, the authors only claim unprecedented precision for one specific sample. What about the others? If the precision is only strong for one sample, this doesn't make a strong case for the overall performance of the proposed system.

Response 18: We appreciate the reviewer's comment.

(1) We fully agree that quantitative comparison with existing literature is essential. To address this, we have prepared a comprehensive literature survey (**Table R4** and **Fig. S5**) summarizing thickness precision achieved by various terahertz metrology techniques.

For battery electrode measurements (**Fig. S5a**), our comb-referenced FDS achieves 70.1 nm precision for 103-µm-thick anode and 465.5 nm for 105-µm-thick cathode at 0.2 s acquisition, compared to ~270 nm (anode) and ~180 nm (cathode) reported by [Ref. 22] using swept-laser-based FDS. With averaging, our precision further improves to 7.8 nm (anode) and 25.2 nm (cathode) at 25.6 s—a regime not explored by conventional swept-FDS systems, where averaged precision data is unavailable in the literature. This demonstrates a one-to-two orders of magnitude improvement over the existing THz-FDS approach for battery electrodes.

For low-loss dielectric and semiconductor samples (**Fig. S5b**), our system achieves comparable single-shot precision of ~106 nm (silicon) relative to TDS-based methods (~30–40 nm for dielectric coatings), while uniquely reaching sub-10 nm precision with averaging—a regime previously unexplored in terahertz metrology.

Table R4. Summary of thickness precision achieved by various terahertz metrology techniques. Precision values are categorized into single-shot and averaged measurements. References correspond to the data points shown in **Fig. S5**. The hyphen (-) indicates that averaged precision data is not available in the literature.

Reference	Method	Sample type	Sample	Precision (single)	Precision (averaged)	Acq. time
(i) This work	Comb-FDS	Battery	Cathode	465.5 nm	25.2 nm at 25.6 s	0.2 s
			Anode	70.1 nm	7.8 nm at 25.6 s	0.2 s
		Semiconductor	Silicon	106.3 nm	8.8 nm at 51.6 s	0.2 s
(ii) Mohammadzadeh, EuRAD (2023) [Ref. 22]	Swept-FDS	Battery	Cathode	~180 nm	-	0.005 s
			Anode	~270 nm	-	0.005 s
(iii) Liebermeister, Nat. Comm (2021) [Ref. 20]	Swept-FDS	Dielectric	PET	~300 nm	-	0.2 s
(iv) Schwenson, JIMTW (2025) [Ref. 21]	Sparse-FDS	Dielectric	Ceramic	~2000 nm	~60 nm at 0.8 s	0.0008 s
(v) Romero, Nat. Comm (2021) [Ref. 52]	FDS	Semiconductor	Silicon	289 nm	-	24 s
(vi) Molter, APR (2021) [Ref. 18]	TDS	Dielectric	Coating	40 nm	-	0.025 s
(vii) Liebermeister, JIMTW (2021) [Ref. 19]	TDS	Dielectric	PET	~30 nm	-	0.127 s

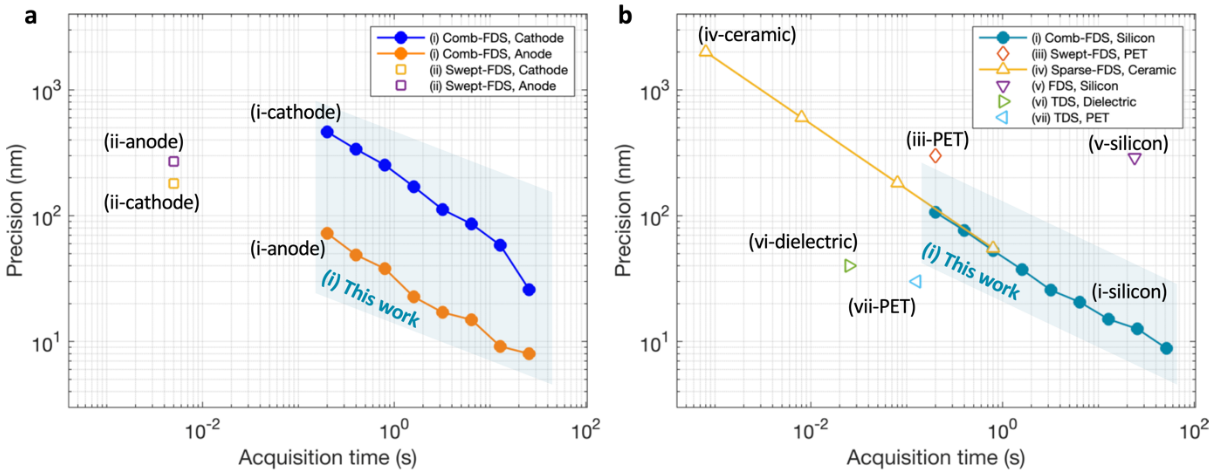

Fig. S5. Performance comparison with state-of-the-art terahertz thickness metrology in terms of thickness precision vs. acquisition time. a Battery electrode measurements; (i) this work, comb-referenced FDS (cathode and anode), (ii) swept-laser-based FDS (cathode and anode)[22]. This work (shaded region) achieves 70.1-nm precision at 0.2-s for anodes, improving to sub-10 nm at 25.6-s integration—demonstrating one-to-two orders of magnitude improvement over swept-laser-based FDS for battery electrode metrology. **b** Low-loss dielectric and

semiconductor measurements; (i) this work, comb-referenced FDS (silicon), (iii) swept-laser-based FDS (PET)[20], (iv) sparse-frequency FDS (ceramic)[21], (v) FDS (silicon)[52], (vi) TDS (dielectric coating)[18], (vii) TDS (PET)[19]. Our system achieves comparable precision of several tens of nanometres in single-shot measurements, while reaching sub-10 nm precision with averaging—a regime previously unexplored in terahertz metrology.

Additionally, we noted that the anode thickness in the original manuscript was incorrectly stated as 68 µm. The correct THz-measured thickness is 103 µm (Case B in **Fig. 4**). We have corrected this throughout the manuscript. The relevant sentences have been revised as follows:

(on page 5, line 103)

> *"... —for instance, 70.1 nm and 465.5 nm precision at 0.2 s integration for a 103-µm-thick anode and a 105-µm-thick cathode, respectively, improving to 7.8 nm and 25.2 nm with 25.6 s integration— ..."*

(on page 24, line 525)

> *"... precision of 70.1 nm within 0.2 s for a 103-µm-thick anode, which improves to 7.8 nm with 25.6 s averaging. Comparable performance was observed for a 105-µm-thick cathode, with precision reaching 465.5 nm at 0.2 s and refining to 25.2 nm ..."*

(2) Regarding the reviewer's concern about whether the precision is specific to one sample, as detailed in our **Response 8** to **Reviewer #3**, the measurement precision systematically depends on the FP mode frequency position governed by sample thickness and refractive index through **Eq. (1)** (**Fig.S4**). All measured samples—both cathodes and anodes across the 50–150 µm range—demonstrate sub-micrometre precision at 0.2 s acquisition, confirming that the performance follows predictable physical principles rather than being sample-specific.

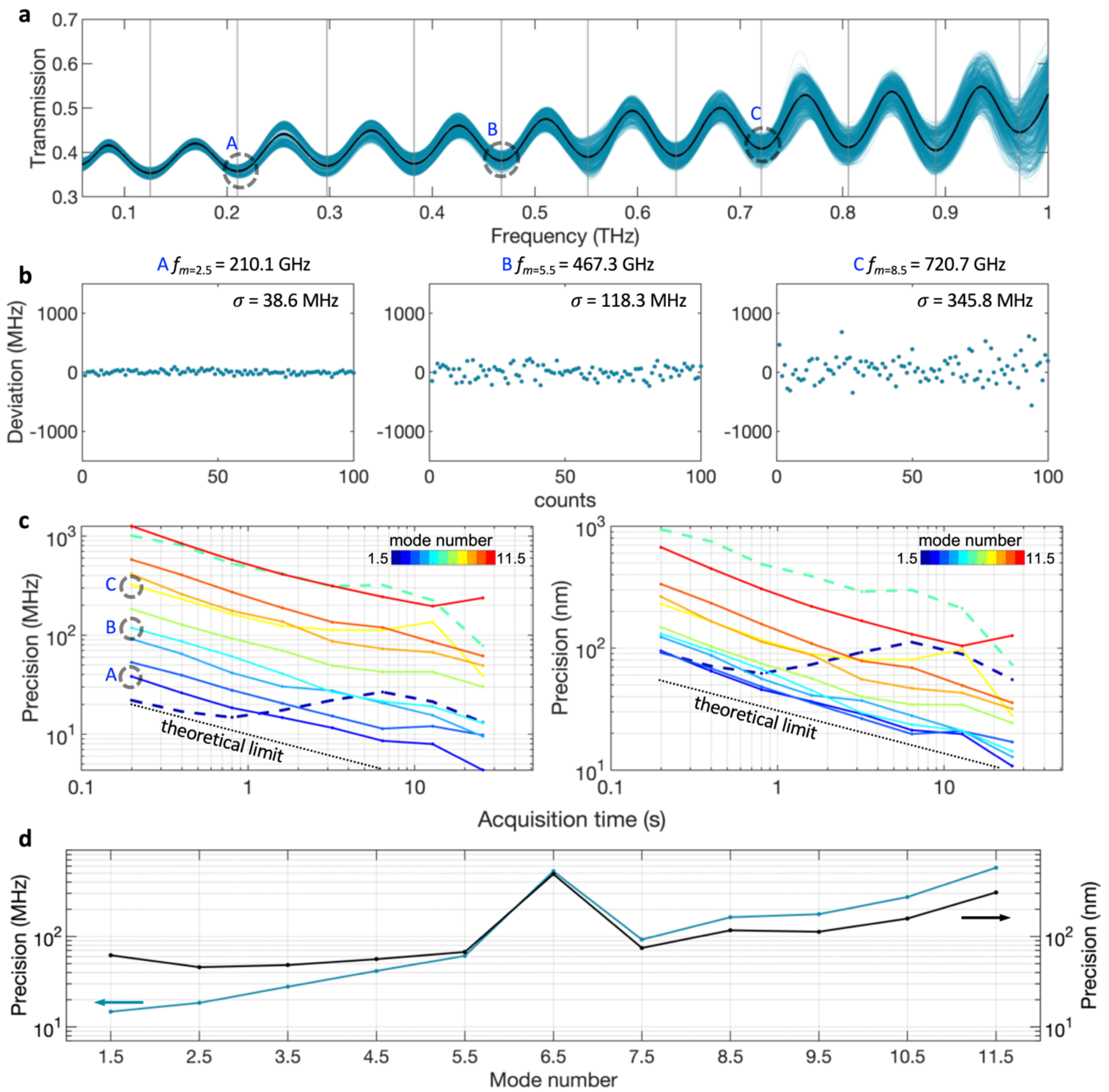

Fig. S3. Precision analysis of frequency and thickness measurements as a function of FP mode number. a Transmission amplitude spectra of a 525-µm thick silicon wafer measured over 1,000 repeated acquisitions across the 0.1–1.0 THz range. The black solid line represents the averaged spectrum, and grey vertical lines indicate the centre positions of destructive FP modes. **b** Deviation of FP mode centre frequencies for three different FP modes (Case A: $f_{m=2.5}$ = 210.1 GHz, Case B: $f_{m=5.5}$ = 467.3 GHz, Case C: $f_{m=8.5}$ = 720.7 GHz). **c** Allan deviation of FP mode frequency precision (left panel) and the retrieved thickness precision (right panel). **d** Frequency precision (blue, left y-axis) and thickness precision (black, right y-axis) as a function of mode number at 0.8-s acquisition time. The thickness precision shows a different behaviour compared to frequency precision due to the inverse dependence on mode number *m*.

Figures

Comment 19. In Fig 2c the caption mentions a histogram which could not be found in the figure

Response 19: We appreciate the reviewer's careful observation.

The histogram was removed during figure revision, but the caption was not updated accordingly.

Following the reviewer's comment, we have revised the **Fig. 2** caption as follows:

(on page 10, line 208)

> *"... **c** Instantaneous (blue) and nominal (black) sweep rate extracted from the data in panel **b**. ..."*

For reference, the original figure including the histogram is shown in **Fig. R7**.

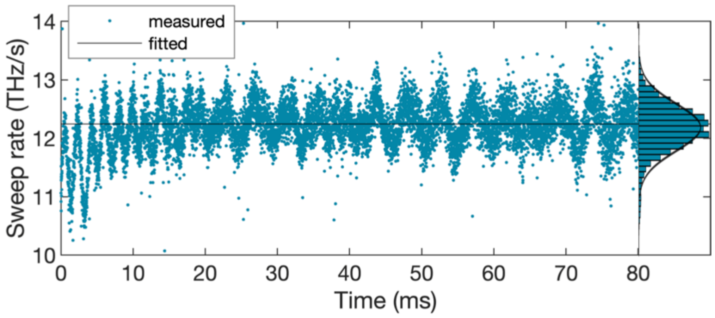

Fig. R7. Instantaneous and nominal sweep rate with Gaussian-fitted histogram. Instantaneous sweep rate (measured, blue dots) and nominal sweep rate (fitted, black line) over time, with a histogram fitted to a Gaussian distribution (black curve) shown on the right.

Comment 20. In the caption of fig 6, the description of subfigures d and e is missing.

Response 20: We appreciate the reviewer's careful observation.

Following the reviewer's comment, we have revised the **Fig. 6** title and caption to include descriptions of parts **d** and **e**:

(on page 21, line 448)

> *"**Fig. 6. THz FP mode monitoring for thickness profiling and dynamic measurements.** ... **d** Schematic of the dynamic thickness measurement setup, with the right-top inset illustrating angle modulation of the fast steering mirror for effective optical path length modulation. **e** Measured effective thickness modulations fitted with a sinusoidal curve, along with its corresponding Fourier transform. M: off-axis parabolic mirror, FSM: fast-steering mirror, VCO: voltage-controlled oscillator."*

Comment 21. In figure S3a-c, consider unifying the styles and colors of the line. Are the samples considered for a, b and c the same?
Comment 22. In figure S3d, consider choosing a different unit for the entries with more than 2 or three decimals to improve readability.

Response 21&22: We appreciate the reviewer's suggestions.

Following the reviewer's comments, we have revised **Fig. S4**. The line styles across **Fig. S4a-c** have been unified: pink lines consistently represent uncontrolled samples (as-fabricated electrodes), while the other colored lines represent controlled samples (calibration-grade specimens) for each respective measurement method. The samples in panels **a-c** are not the same specific specimens, but rather

illustrative cases comparing general electrode samples under uncontrolled and controlled conditions to demonstrate how sample quality affects overall measurement uncertainty.

Additionally, decimal values with many significant figures have been converted into scientific notation (e.g., 0.000001 μm → 1×10^{-6} μm) to improve readability.

(on page 36, line 829)

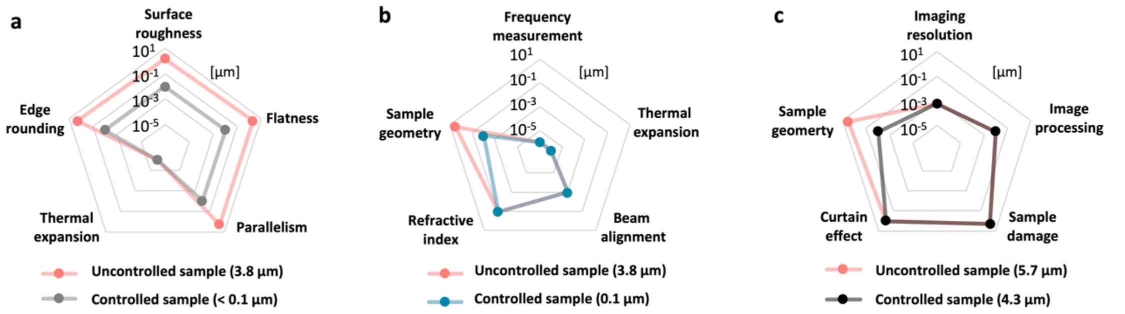

d

		Uncertainty errors	Value	Value for t = 100 μm
Sample		Uncertainty of surface roughness	1.5 μm	1.5 μm
		Uncertainty of flatness, parallelism, and edge rounding<br>Uncertainty of flatness = 1.5 μm<br>Uncertainty of parallelism = 1.5 μm<br>Uncertainty of edge rounding = 1.5 μm	3.5 μm	3.5 μm
		Uncertainty related to Thermal expansion of electrodes = $t \times 10^{-8}$<br>Thermal expansion coefficient of electrodes = 1×10^{-6} K^{-1}<br>Surface temperature = 15×10^{-3} K	$t \times 10^{-8}$	1×10^{-6} μm
Combined standard uncertainty (k=1)			$\{(t \times 10^{-8})^2+(3.8\ \mu m)^2\}^{1/2}$	3.8 μm
THz interferometer	Light source	Uncertainty related to frequency = t × 10^{-8}<br>Repetition rate = 10^{-12}<br>Carrier-envelope offset frequency = 10^{-13}<br>Beat frequency between swept laser and comb = 10^{-8}	$t \times 10^{-8}$	1×10^{-6} μm
	Interferometer	Uncertainty related to beam alignment = $t \times 10^{-5}$<br>Uncertainty of sample incidence angle = 1°	$t \times 10^{-5}$	0.001 μm
		Uncertainty related to thermal expansion of optical fiber = $t \times 10^{-9}$<br>Thermal expansion coefficient of optical fiber = 5.0×10^{-7} K^{-1}<br>Surface temperature = 15×10^{-3} K	$t \times 10^{-9}$	1×10^{-7} μm
		Uncertainty related to refractive index of sample = $t \times 10^{-3}$<br>Uncertainty of refractive index = 10^{-3}	$t \times 10^{-3}$	0.1 μm
Combined standard uncertainty (k=1)			$t \times 10^{-3}$	0.1 μm
SEM imaging	Imaging system	Uncertainty related to imaging resolution = 0.0006 μm	0.0006 μm	6×10^{-4} μm
		Uncertainty related to image processing and analysis<br>Uncertainty of magnification calibration = 1%	$t \times 10^{-4}$	0.01 μm
	Cross-sectioning	Uncertainty related to sample damage and delamination<br>Uncertainty of sample damage = 3.0 μm<br>Uncertainty of delamination = 3.0 μm	4.2 μm	4.2 μm
		Uncertainty related to curtain effect of multilayer sample = $t \times 10^{-2}$<br>Uncertainty of sample perpendicularity = 5°	$t \times 10^{-2}$	1 μm
Combined standard uncertainty (k=1)			$\{(t \times 10^{-2})^2+(4.2\ \mu m)^2\}^{1/2}$	4.3 μm

Fig. S4. Uncertainty analysis in LIB electrode thickness measurements. a *Sample-related uncertainties, where surface roughness, flatness, parallelism, and edge rounding contribute micrometre-scale errors, while thermal expansion is negligible. Sample-related uncertainties can be minimized using calibration-grade specimens, but LIB electrodes are not standardized by NMIs. Panels* ***b*** *and* ***c*** *show comparisons between uncontrolled and controlled samples (uncertainty < 0.1 μm).* ***b*** *THz interferometry uncertainties, dominated by variations in the sample's refractive index; errors from the light source and interferometer are minimal.* ***c*** *Cross-sectional SEM imaging, where uncertainty is primarily due to cross-sectioning accuracy, while imaging system noise is negligible.* ***d*** *Summary of uncertainty sources associated with the sample (**a**), THz interferometer (**b**), and cross-sectional SEM imaging (**c**).*

[Author's responses to Reviewer #4]

Reviewer #4 (Remarks to the Author):

I co-reviewed this manuscript with one of the reviewers who provided the listed reports. This is part of the Nature Communications initiative to facilitate training in peer review and to provide appropriate recognition for Early Career Researchers who co-review manuscripts.

Young-Jin KIM
Professor of Department of Mechanical Engineering,
Korea Advanced Institute of Science and Technology (KAIST)
291 Daehark-ro, Yuseong-gu, Daejeon 34141, Republic of Korea
E-mail: yj.kim@kaist.ac.kr, Tel: +82 42-350-3017, Mobile: +82 10-7613-2893

24th March 2026

RE: Submission of our revised manuscript - **NCOMMS-25-94790A**

Dear Reviewers,

We are submitting our second-revised manuscript entitled "**Nanometre-precision terahertz interferometry for battery electrode metrology**", in which modifications have been made to the first-revised manuscript to address the remaining reviewer's concerns.

We are grateful to all reviewers for their positive evaluations. Reviewers #1 and #2 have recommended acceptance, while Reviewer #3 has raised one additional point regarding the comparison between THz and SEM measurements, which has now been fully addressed in the revised manuscript.

Finally, enclosed is the summary of the author's responses (written in blue) point by point with regard to all the reviewers' comments and remarks (reproduced in black).

Thank you for your kind consideration.

Best regards,

Young-Jin Kim, Ph.D.
Professor of Department of Mechanical Engineering,
Korea Advanced Institute of Science and Technology (KAIST),
291 Daehark-ro, Yuseong-gu, Daejeon 34141, Republic of Korea
E-mail: yj.kim@kaist.ac.kr
Tel: +82-42-350-3017

[Author's responses to Reviewer #1]

I am satisfied with the authors' revisions. They have addressed all of my previous concerns thoroughly and thoughtfully. **The manuscript is now clear, well-argued, and scientifically robust. I recommend acceptance.**

The authors very much appreciate the reviewer's positive evaluation.

[Author's responses to Reviewer #2]

The authors provide a very nicely revised version of the initial manuscript. All the points raised during the first round of review have sufficiently been addressed and many important details have been added to the manuscript, especially a proper comparison to the state of the art with related technologies. Thus, **I see the manuscript now fit for publication and congratulate the authors on this beautiful work.**

The authors very much appreciate the reviewer's positive evaluation.

[Author's responses to Reviewer #3]

The authors have addressed all my comments and I appreciate the extensive revisions made.

Only one point remains regarding the comparison with SEM-based measurements: In their response to my comment #11, the authors describe that both methods, THz and SEM, are fundamentally different and that the differences in measurement stem from the sample preparation, porosity and measurement location, which was different between THz and SEM.

This raises several questions.

(Q1) What is important to be measured in this use-case? Only the layer thickness, or is the porosity also important to be monitored? If porosity is important, too, can it be extracted from THz measurements?

(Q2) And for the thickness, is the absolute thickness including porosity important or only the solid thickness? And can the porosity be considered in the THz measurements to get the correct thickness?

(Q3) Lastly, regarding the methodology. Although SEM can of course only access cross-sections, while THz averages over an area, I think it would be better for the comparison to have both measurements taken on the same site. Why was it not possible to do the THz measurement first and then perform the destructive SEM analysis?

I think, discussing these points would clarify the benefits of the proposed method and strengthen the paper. **Upon addressing these points, I can recommend the paper for publication.**

Response: We appreciate the reviewer's comment.

(Q1) What is important to be measured in this use-case? Only the layer thickness, or is the porosity also important to be monitored? If porosity is important, too, can it be extracted from THz measurements?

(A1) In battery electrode manufacturing, both coating thickness and porosity are critical quality parameters. Thickness directly governs stacking precision during cell assembly and energy density, while porosity determines ion transport kinetics and electrochemical performance.

Our THz measurements are inherently sensitive to porosity through the effective refractive index n. As shown in **Fig. 5** and **Supplementary Fig. 1**, the extracted refractive indices (n = 2.9 for the cathode, n = 6.0 for the anode) reflect the bulk composite properties including porosity, consistent with effective medium theories such as the Bruggeman model [Ref. A].

However, independently separating thickness and porosity from a single FP measurement remains challenging, because the FP resonance condition directly yields the optical thickness nt rather than n and t individually. To decouple these two parameters, additional information is required. The polarization-resolved spectroscopy approach discussed in our manuscript—exploiting the distinct Fresnel coefficients for s- and p-polarizations at oblique incidence—offers a promising pathway toward independent determination of n and t, and consequently, porosity extraction. We are planning to explore this capability in future work.

Reference:

[Ref. A] G. A. Niklasson, C. G. Granqvist, and O. Hunderi, "Effective medium models for the optical properties of inhomogeneous materials," Appl. Opt. 20, 26-30 (1981)

Following the reviewer's comment, we have added a discussion on porosity–refractive index coupling and the associated measurement constraints in the revised manuscript:

(on page 19, line 421)

> *"... In particular, porosity directly influences the effective refractive index through dielectric mixing, where increased porosity reduces n; however, because the FP resonance condition yields the optical thickness nt, independently separating n and t—and thus extracting porosity—requires additional measurement constraints. To address this, polarization-resolved reflection spectroscopy can be employed. Oblique incidence (e.g., at a 45° angle) induces different Fresnel transmission and reflection coefficients[38] for s- and p-polarized light, which manifest as distinct features in the polarization-resolved spectra. ..."*

<u>(Q2) And for the thickness, is the absolute thickness including porosity important or only the solid thickness? And can the porosity be considered in the THz measurements to get the correct thickness?</u>

(A2) From a process control perspective, the primary quantity of interest is the total physical thickness of the coating layer including porosity, as it directly determines cell stacking dimensions and serves as the input for calendaring. The THz measurement converts optical thickness *nt* to physical thickness *t* using the effective refractive index, which accounts for all constituents including pore space. Therefore, both THz and SEM measurements target the same physical quantity—the total coating thickness including porosity—although their measured values can differ due to differences in measurement location and spatial averaging.

Following the reviewer's comment, we have clarified that both measurement methods target the same physical quantity in the revised manuscript:

(on page 15, line 320)

> *"... The error bars indicate the measurement precision for t_{THz} and the spatial non-uniformity for t_{SEM}, respectively. Both t_{THz} and t_{SEM} represent the total coating thickness including porosity, as t_{THz} inherently accounts for pore space through the effective refractive index. The t_{SEM} values were determined by averaging the thickness across the entire cross-sectional SEM images after binarization processing. ..."*

<u>(Q3) Lastly, regarding the methodology. Although SEM can of course only access cross-sections, while THz averages over an area, I think it would be better for the comparison to have both measurements taken on the same site. Why was it not possible to do the THz measurement first and then perform the destructive SEM analysis?</u>

(A3) We agree that co-located measurements would provide the most rigorous comparison. In this study, two practical constraints prevented this: (i) the THz beam spot (~5 mm diameter) spatially averages over a much larger area than the SEM cross-section (tens of μm width), so a fundamental mismatch in spatial sampling persists even at the same nominal location; and (ii) sample cutting required for ion milling can introduce mechanical deformation or damage to the electrode, particularly for uncalendered films with low mechanical integrity, potentially altering the local thickness at the measurement site.

These factors, combined with the intrinsic thickness non-uniformity of the electrodes, account for the observed discrepancies between THz and SEM thickness values.

Following the reviewer's comment, we have clarified the spatial sampling mismatch between THz and SEM in the revised manuscript:

(on page 17, line 369)

> *"... However, SEM imaging still exhibits larger error bars due to the inherent spatial non-uniformity of the electrode coating, as well as artifacts introduced during the ion milling process and the mismatch in spatial sampling between the millimetre-scale THz spot and the micrometre-width SEM cross-section."*

[Author’s responses to Reviewer #4]

Reviewer #4 (Remarks to the Author):

I co-reviewed this manuscript with one of the reviewers who provided the listed reports. This is part of the Nature Communications initiative to facilitate training in peer review and to provide appropriate recognition for Early Career Researchers who co-review manuscripts.
